# Deciphering Evolution, Function, and Observation of Crystallization-Driven Self-Assembly

**DOI:** 10.1021/acs.chemrev.5c00298

**Published:** 2025-10-13

**Authors:** Tianlai Xia, Laihui Xiao, Yujie Xie, Andrew P. Dove, Rachel K. O’Reilly

**Affiliations:** † School of Chemistry, 1724 University of Birmingham, Edgbaston, Birmingham B15 2TT, U.K.; ‡ School of Medicine, 34747 Shanghai University, Shanghai 200444, China

## Abstract

Crystallization-driven self-assembly (CDSA) offers precise control over the size, shape, and hierarchical organization of polymeric nanostructures by harnessing the crystallization of a core-forming block. Unlike conventional self-assembly, CDSA favors the formation of low-curvature morphologies, such as fibers and platelets, with exceptional uniformity. This review highlights key CDSA strategies, including seeded growth, self-seeding, and polymerization-induced CDSA, along with factors influencing assembly, such as polymer composition, solvent, temperature, and additives. We summarize advanced characterization techniques—spanning light scattering, microscopy, spectroscopy and fluorescence imaging—and computational approaches, including Monte Carlo and Brownian dynamics simulations, for understanding assembly mechanisms and predicting morphologies. Finally, we discuss emerging applications in biomedicine, catalysis, optoelectronics, and functional materials, and outline future challenges in precision control, multitechnique characterization, and scalable synthesis. By integrating mechanistic insights, advanced characterization, and application-driven design, this review establishes a comprehensive foundation for future development of CDSA-based functional materials.

## Graphical abstract

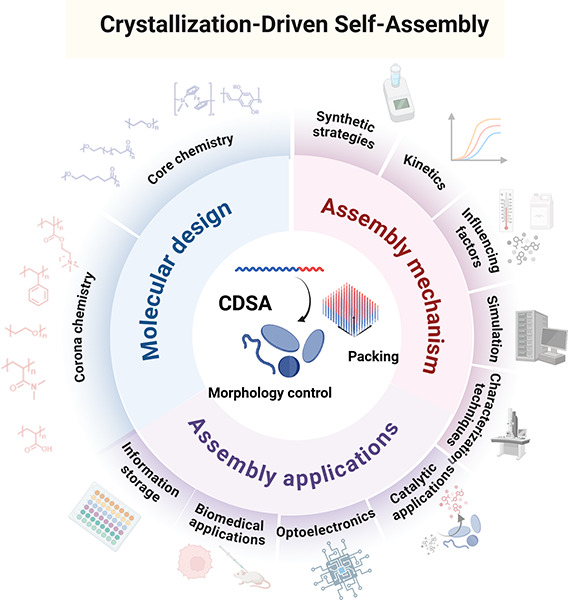

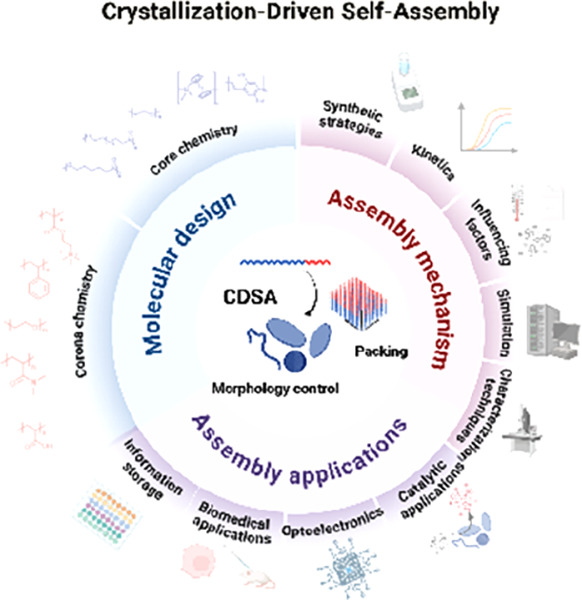

## 1 Introduction

Crystallization-driven self-assembly (CDSA) has emerged as a powerful strategy for constructing well-defined polymeric nanostructures with precise control over size, morphology, and hierarchical organization.^1–3^ Unlike conventional self-assembly driven by solvophilicity-solvophobicity balance, which typically results in dynamic, equilibrium-based structures with limited directional control, CDSA leverages polymer crystallization to guide the formation of highly ordered, anisotropic assemblies.^4^ This process enables the fabrication of diverse architectures, including one-dimensional (1D) cylindrical micelles and two-dimensional (2D) platelet structures, with tunable properties.^5^

Since its introduction by Manners and Winnik, CDSA has evolved into a versatile platform for designing functional nanomaterials.^6^ Recent advancements, including hierarchical self-assembly and controlled nanoparticle synthesis, have expanded its potential applications across various fields.^7^ The ability to precisely control nanoparticle shape opens up unprecedented opportunities for tailoring material properties based on morphology.

This review provides a critical and systematic discussion of the CDSA process, focusing on mechanistic understanding, advanced characterization, computational modeling, emerging applications, and future challenges. It first examines the fundamental mechanisms governing CDSA, including synthetic strategies, factors influencing self-assembly and assembly kinetics, offering a comprehensive view of how crystalline block copolymers form well-defined nanostructures under controlled conditions. To complement this, the review highlights post-assembly and characterization techniques, such as scattering techniques, microscopy, fluorescence imaging, and spectroscopy, which provide essential insights into structural evolution, morphological transitions, and kinetic processes during CDSA. In parallel, computational approaches, including Brownian dynamics and Monte Carlo simulations, are discussed to demonstrate how modeling contributes to understanding nanoscale assembly mechanisms and predicting morphology and assembly pathways. Beyond mechanistic and characterization insights, the review explores emerging applications of CDSA-derived assemblies in biomedicine, catalysis, optoelectronics, and functional additives, illustrating how morphological control and tunable functionality make these materials highly suitable for diverse technological applications. Finally, the review identifies key challenges and future directions, particularly in improving morphological precision, expanding the range of accessible structures, enhancing scalability, and developing functional, application-ready materials. By integrating mechanistic understanding, advanced characterization, computational modeling, and application-driven design, this review aims to bridge the gap between fundamental knowledge and practical implementation, establishing a comprehensive foundation for future advancements in CDSA-based nanomaterials (Scheme 1).

**Scheme 1:**
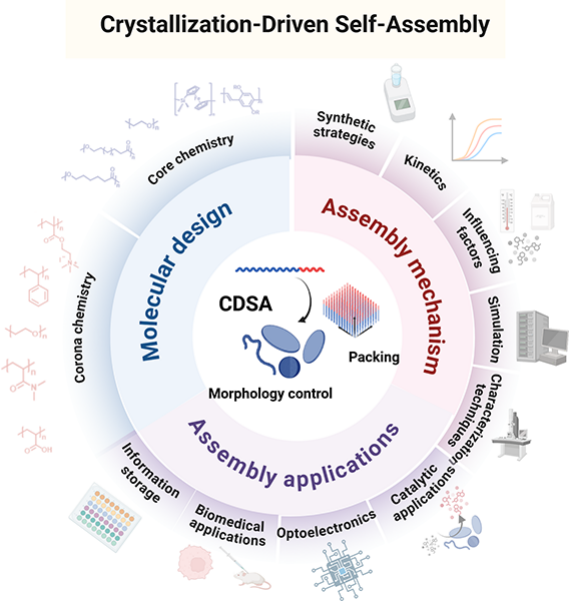
Overview of CDSA Highlighting Key Molecular Design Principles, Assembly Mechanism, and Assembly Applications; Created with BioRender

## 2 General Self-Assembly

### 2.1 Conventional Self-Assembly

Nature excels in constructing intricate structures across a wide range of scales, from the nanoscale to the macroscale. Inspired by these natural systems, block copolymer (BCP) self-assembly has emerged as a powerful strategy for fabricating well-defined nanostructures. BCPs possess the intrinsic ability to undergo microphase separation, enabling the formation of complex morphologies.^8–15^ Traditional amphiphilic BCP self-assembly typically yields spherical micelles, worm-like structures, or vesicles.^16–18^ However, the incorporation of a crystallizable polymer as the core-forming block fundamentally alters the assembly behavior, favoring low-interfacial-curvature morphologies such as cylinders and platelets.^1,19,20^ This transformation is primarily driven by the crystallization free energy (ΔG) of the core-forming block, a phenomenon known as CDSA.^2,5,21–26^ While CDSA shares key principles with conventional solution self-assembly—where morphology is influenced by factors such as block composition, solvophobicity, degree of polymerization (DP), and packing parameters—it is uniquely governed by the dominant crystallization force of the core-forming block (Table 1). This crystallization-driven mechanism enables better regulation of nanostructure dimensions, stability, and hierarchical organization, distinguishing CDSA as a versatile approach for designing functional polymeric assemblies.^3,27,28^

**Table 1:** Comparison between Self-Assembly of BCPs with Amorphous and Crystalline Core

**Core forming block**	**Amorphous core** ^a^	**Crystalline core**
Driving force	G_core_ + G_interface_ + G_corona_	G_core_ + G_interface_ + G_corona_ + G_crystallization_
Morphology	Spheres, worms, vesicles	Cylinders, lamellas
Interfacial curvature	High	Low
Factors	Dependent on core chain stretching, interfacial energy, and corona chain stretching	Mainly dependent on the crystallization ability of the core, also associated with core chain stretching, interfacial energy, and corona chain stretching

a G_core_ represents the energy of repulsion between the core and solvent. G_interface_ represents the energy of stretching between the core and corona. G_corona_ represents the energy of attraction between the corona and solvent. G_crystallization_ refers to the energy of core-forming block crystallization.

### 2.2 Crystallization-Driven Self-Assembly (CDSA)

In a typical CDSA process, BCPs containing at least one crystallizable segment are dissolved in a selective solvent that is favorable for the corona-forming block but poor for the crystalline core-forming segment.^2^ The solution is heated above the melting temperature (*T*_m_) of the crystalline block to remove any pre-existing crystallinity, ensuring uniform dissolution.^3^ Upon cooling below the crystallization temperature, the crystalline chains self-nucleate and fold into ordered micellar structures, forming well-defined core–shell assemblies such as cylindrical micelles and platelets.^2,5^

The first example of block copolymer crystallization in dilute solution was reported by Keller and Kovacs in 1966, in which poly­(ethylene oxide) (PEO)-based block copolymers were found to form crystals in dilute solution.^29^ Over three decades later, Manners and Winnik pioneered the use of crystallizable polyferrocenylsilane (PFS)-based block copolymers to form cylindrical micelles, formally establishing CDSA in 1998.^6^ In 2007, they introduced the concept of living CDSA, demonstrating controlled cylindrical micelle length by employing presonicated seeds as nuclei.^30^ This approach was later extended in 2009 to enable the formation of two-dimensional (2D) nanostructures.^31^ By 2017, Manners and Winnik further expanded CDSA through polymerization-induced CDSA (PI-CDSA), allowing scalable synthesis of cylindrical micelles.^32^ More recently in 2025, the O’Reilly and Dove groups developed a rapid seed preparation method using flash-freezing, followed by the integration of seed preparation and seeded growth within a continuous-flow cascade, which enables the high-throughput fabrication of platelets directly from polymers.^33^

#### 2.2.1 Core Chemistry

CDSA enables the fabrication of well-defined nanostructures by leveraging the crystallization of core-forming polymer blocks. The selection of the core-forming polymer is crucial, as its crystallization behavior dictates the assembly process, morphological transitions, and long-term stability of the nanostructures.^2,3^ Over the past three decades, five primary classes of core-forming materials have been extensively studied: metal-containing polymers,^34–36^ polyesters,^37–39^ conjugated polymers,^40–42^ liquid crystalline polymers,^43–45^ and other crystallizable polymers (Figure 1).^46–49^ Each of these classes exhibit distinct crystallization behaviors that significantly influence the final morphology and functionality of the assembled structures.

**Figure 1:**
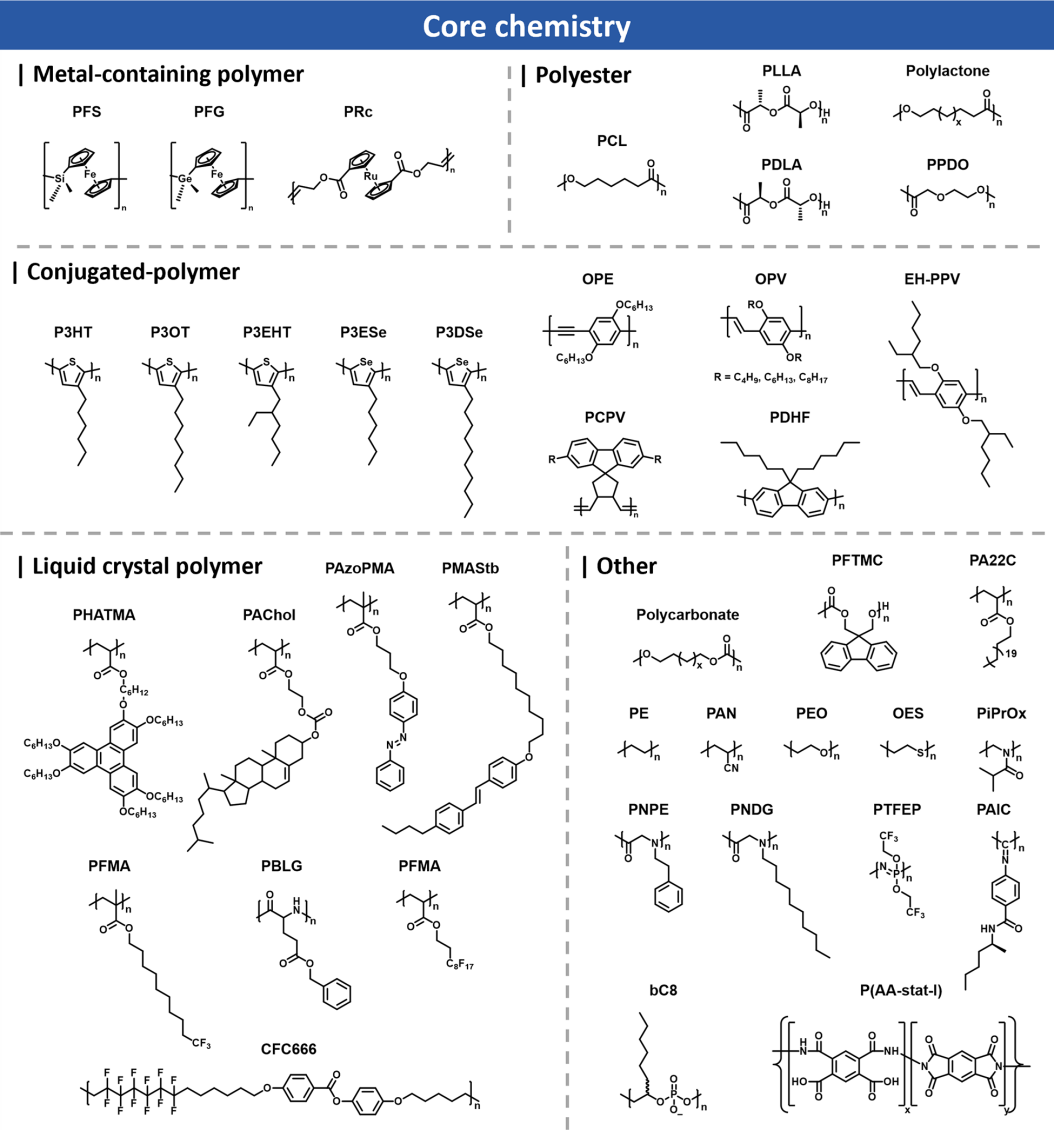
Overview of different core chemistries used in CDSA.

The self-assembled morphology in CDSA is largely determined by the crystallinity and lattice symmetry of the core-forming block. Polymers whose crystalline phases exhibit high symmetry often form regular structures, such as rectangular or hexagonal platelets, due to uniform molecular packing and isotropic crystal growth.^19,50^ In contrast, polymers with lower symmetry or directional intermolecular interactions tend to exhibit anisotropic growth, leading to branched, twisted, or hierarchical morphologies.^51–53^ A mechanistic understanding of these structure–morphology relationships is essential for the rational design of nanomaterials with tailored properties and for expanding the application scope of CDSA.^2^

##### 2.2.1.1 Metal-Containing Polymers

Metal-containing polymers represent the most extensively studied class of crystallizable core-forming materials in CDSA. Among them, PFS serves as the prototypical example.^54–57^ First introduced by Manners and Winnik, PFS has demonstrated exceptional capabilities in CDSA,^6^ allowing for the formation of highly uniform and monodisperse nanostructures. The ferrocene moieties within PFS enhance its crystallization tendency, enabling the formation of well-defined cylindrical micelles, 2D platelets, and hierarchical nanostructures.^58–69^ Further studies on PFS-based CDSA enabled good control over nanostructure size, morphology, and hierarchical organization, establishing PFS as a widely used core-forming polymer in CDSA.^30,70–90^

Building upon the success of PFS, structurally related metal-containing polymers have been explored to expand the functional scope of CDSA.^91,92^ One such example is poly­(ferrocenyldimethylgermane) (PFG), which replaces silicon (Si) in the PFS backbone with germanium (Ge).^93^ While maintaining similar self-assembly behavior, PFG exhibits a lower crystallization temperature, influencing the kinetics of self-assembly. Studies on block comicelles of PFS and PFG have demonstrated heteroepitaxial growth, where the distinct crystallization rates of these two core-forming polymers dictate the final morphology.^93^

A ruthenocene-based polymer (PRc), accessed by ring-opening metathesis polymerization represents another variation of metal-containing polymers for CDSA, incorporating ruthenocene (Rc) moieties.^94^ Compared to PFS and PFG, PRc exhibits unique redox and catalytic properties, making it a promising material for advanced functional applications. Studies exhibited that PRc-based assemblies display a different crystallization tendency due to stronger metal–ligand interactions, influencing their morphological evolution.^95^

The presence of metal centers within these polymers provides additional advantages, including enhanced thermal and oxidative stability, redox tunability, and magnetic or electronic functionality.^57^ PFS and its analogs exhibit excellent thermal resistance, enabling the fabrication of stable nanostructures under varying environmental conditions.^62^ The redox activity of the ferrocene units in PFS and PRc introduces opportunities for designing stimuli-responsive materials, where controlled oxidation and reduction can modulate assembly behavior or material properties. This tunability is particularly beneficial in catalysis, where metal-containing CDSA assemblies serve as nanoscale catalytic platforms with well-defined active sites.

##### 2.2.1.2 Polyesters

Polyesters are a key class of biodegradable and biocompatible core-forming polymers in CDSA.^38,96–102^ Among them, poly­(ϵ-caprolactone) (PCL) and polylactide (PLA) have been widely utilized due to their well-defined crystallinity, tunable chain folding behavior, and controlled degradation profiles.^14,103^

PCL is a semicrystalline polymer with a low glass transition temperature and moderate melting temperature, making it highly adaptable for CDSA.^104,105^ It forms a variety of nanostructures, including 1D fibers, 2D platelets, and hierarchical assemblies.^49,106–117^ By adjusting block composition, molecular weight, and processing conditions, the crystallization behavior of PCL can be finely tuned to control aspect ratios, platelet dimensions, and fiber lengths, which influence the stability and properties of the resulting assemblies.^39,118–129^ PCL-based CDSA has been widely explored in biomedical applications, particularly in controlled drug delivery and tissue engineering, where its biodegradability and tunable degradation rates offer significant advantages.^130–136^

PLA is another widely investigated polyester in CDSA, capable of forming cylindrical micelles, platelets, and complex hierarchical assemblies.^37,137–151^ Its chiral building block, lactic acid, allows stereocomplex crystallization when enantiomeric poly­(l-lactide) (PLLA) and poly­(d-lactide) (PDLA) are combined, resulting in enhanced mechanical strength and thermal stability compared to homochiral PLA.^152–162^ This property makes PLA-based assemblies ideal for structural reinforcement and phase-separated self-assembly and enhancement of mechanical properties of hydrogels.^163,164^ In biomedical applications, PLA’s biocompatibility and bioresorbability make it well-suited for drug delivery systems, nanocomposite scaffolds, and implantable medical devices.^152^ The ability to control PLA’s degradation behavior further expands its use in tissue engineering and regenerative medicine, where sustained release and structural integrity are critical.^165–169^ The continued development of PLA-based nanostructures will drive advancements in high-performance materials and biomedical applications.

##### 2.2.1.3 Conjugated Polymers

Conjugated polymers have attracted significant interest in CDSA due to their unique electronic properties, strong π–π interactions, and structural versatility.^170–174^ Unlike conventional crystallizable polymers, their structural and physicochemical properties are influenced not only by crystallization ability but also by electronic delocalization and charge transport characteristics.^50,175–178^ These features make them promising candidates for organic electronics, light-emitting devices, and energy storage applications.^41,179–186^

Poly­(3-hexylthiophene) (P3HT) is among the most widely studied conjugated polymers in CDSA, owing to its high charge mobility and well-defined crystalline packing.^187–193^ The self-assembly of P3HT-based block copolymers enables the formation of highly ordered 1D nanofibers and 2D nanosheets with tunable optoelectronic properties.^194–196^ These nanostructures exhibit enhanced charge transport behavior, making them highly relevant for organic photovoltaics, field-effect transistors, and photodetectors.^197–199^

Oligo­(*p*-phenylenevinylene) (OPV),^200–206^ oligo­(*p*-phenylene ethynylene) (OPE),^207–215^ and poly­(*p*-phenylenevinylene) (PPV),^216,217^ poly­(cycloparaphenylenevinylene) (PCPV)^42,218–221^ derivatives further broaden the scope of conjugated polymer-based CDSA.^222–225^ Their self-assembly is primarily governed by π–π stacking interactions, leading to the formation of crystalline nanostructures with well-defined electronic and optical properties.^226^ For example, PPV derivatives, known for their strong fluorescence emission, are widely used in light-emitting applications, while PCPV-based nanostructures show potential for flexible semiconducting materials with tailored electronic bandgaps.^218^

Polyfluorene (PF) and its derivatives also exhibit remarkable self-assembly behavior, forming highly ordered nanostructures with exceptional photophysical properties. These materials have been extensively utilized in organic light-emitting diodes (OLEDs) and other optoelectronic devices. Moreover, their unique structural and electronic characteristics have recently attracted interest in catalytic applications, expanding the functional potential of CDSA-derived conjugated polymer nanostructures.^170,227–232^

##### 2.2.1.4 Liquid Crystalline Polymers

Liquid crystalline polymers (LCPs) represent a distinct class of crystallizable materials in CDSA, characterized by their ability to maintain orientational or positional long-range order while retaining a high degree of molecular mobility.^45,233^ This unique combination arises from their rigid, rod-like molecular structures, which drive the formation of highly ordered nanostructures.^234^ The self-assembly behavior of LCPs is primarily dictated by their mesogenic cores, which promote directional crystallization and hierarchical organization.^235,236^ These properties make LCPs highly promising candidates for advanced optical materials, responsive nanostructures, and stimuli-responsive devices.^237^ Several kinds of LCPs have been investigated in CDSA, including poly­(γ-benzyl-l-glutamate) (PBLG),^238^ poly­(α-cholesteryl) (PAChol),^239^ poly­(perfluorooctyl) (PFO),^43^ and poly­(azobenzene) (PAzo).^240^

PBLG, a widely studied LCP in CDSA, adopts an α-helical conformation as a rod–coil block copolymer, which strongly influences its self-assembly.^241^ The rigidity of the polypeptide backbone promotes intermolecular interactions, facilitating the formation of well-ordered 2D platelets and fibrous structures. Hierarchical organization arises from a combination of hydrogen bonding and crystallization forces.^242^ Morphology and functionality can be finely tuned by adjusting polymerization degree or solvent conditions.^243^

PAChol has attracted considerable interest in CDSA due to its mesogenic properties. It consists of a cholesterol-based mesogenic core attached to a methacrylate backbone, imparting both liquid crystalline ordering and polymeric flexibility.^244^ This structural arrangement facilitates self-assembly into highly ordered nanostructures driven by directional crystallization and anisotropic interactions.^244,245^ Through controlled molecular alignment control, PAChol-based CDSA structures have been tailored for applications in liquid crystal displays, photonic materials, and optical sensors.^239^

PFO is another notable LCP explored in CDSA. Its highly fluorinated side chains impart unique physical and chemical properties, including strong hydrophobicity, low surface energy, and high chemical stability.^24^ The rigidity of perfluorinated segments promotes liquid crystalline ordering, which strongly influences its crystallization behavior.^237^ Phase-separation between fluorinated and nonfluorinated domains leads to well-defined nanostructures, making PFO-based assemblies particularly valuable for applications such as superhydrophobic coatings, low-friction surfaces, and optical materials.^43,246–250^

PAzo exhibits light-responsive behavior in CDSA, with the azobenzene moiety undergoing reversible trans–cis photoisomerization upon exposure to specific wavelengths, enabling dynamic control over molecular ordering and self-assembly.^251^ Polyazobenzene derivatives display anisotropic crystallization, forming well-aligned platelets and fibers with tunable dimensions. Incorporating azobenzene units into CDSA architectures allows for responsive shape transformations, reversible adhesion, and controlled molecular alignment, expanding the functional scope of these assemblies.^44,240,251,252^

##### 2.2.1.5 Other Crystallizable Polymers

Beyond the primary classes of core-forming materials, various crystallizable polymers have been investigated in CDSA, broadening the range of achievable nanostructures.^253–267^ These include polyethylene (PE),^46^ poly­(ethylene oxide) (PEO),^197^ polyacrylonitrile (PAN),^48^ polycarbonate (PC),^49^ and poly­(fluorenetrimethylenecarbonate) (PFTMC),^268^ each exhibiting unique crystallization behaviors and contributing to different self-assembly pathways.

PE has been widely studied for its semicrystalline nature, forming well-ordered fibrillar and platelet-like structures through CDSA. This behavior of PE-based BCPs is influenced by chain length, dispersity, and processing conditions, making it an attractive material for robust and mechanically durable nanostructures.^46,269–284^

PEO, known for its excellent solubility in aqueous and organic media, has been explored in CDSA for forming core–shell micelles and lamellar structures. The balance between crystallization and solvation effects allows control over morphology, making PEO-based assemblies suitable for drug delivery and bioactive materials.^285–291^

PAN exhibits strong crystallization tendencies due to its rigid nitrile groups, forming highly ordered nanostructures in CDSA, which is influenced by polymer molecular weight and processing temperature, leading to controlled fiber-like and platelet morphologies.^48,292^

PC has also been investigated in CDSA, with its crystallization behavior largely dependent on molecular composition.^293^ While conventional PC lacks strong crystallization tendencies, PFTMC, a fluorene-based polycarbonate derivative, exhibits excellent self-assembly behavior due to cooperative effects from carbonate chain folding and π–π stacking of fluorene groups.^268,294–304^ PFTMC has shown great promise in biomedical applications, including DNA/RNA delivery and magnetic resonance imaging.^305–307^ However, the underlying crystallization mechanism of PFTMC remains unclear and requires further investigation.

Peptide-based polymers introduce an additional dimension to CDSA, as they self-assemble through secondary structural motifs such as β-sheets, α-helices, and coiled coils.^308^ These ordered structures drive hierarchical assembly into fibrillar, sheet-like, and tubular morphologies, mimicking protein architectures. Their inherent biocompatibility and biodegradability enhance their potential in drug delivery, tissue engineering, and biomolecular sensing.^67,309^

Therefore, the selection of a core-forming block is a critical determinant in CDSA, influencing the resulting nanostructure morphology, crystallization kinetics, and functional properties. The diversity of crystallizable polymers—ranging from metal-containing polymers and polyesters to conjugated and liquid crystalline polymers—offers an expansive toolkit for designing tailored nanomaterials (Table 2). The ongoing exploration of novel core-forming blocks and their hierarchical organization will continue to advance the field.

**Table 2:** Summary of Core-Forming Chemistries in CDSA

**Core chemistry**	**Key features/advantages**	**Limitation**	**Potential applications**
Metal-containing polymers	High crystallinity; tunable redox, magnetic, electronic properties; thermal and oxidative stability (e.g., PFS, PFG, PRc)	Involves metals—biocompatibility and degradation may be limited; synthesis can be complex	Catalysis, electronic and magnetic materials, stimuli-responsive assemblies
Polyesters	Biodegradable, biocompatible; tunable crystallization and degradation; widely used (e.g., PCL, PLA); stereocomplexation possible	Mechanical strength may be lower than metal-containing polymers; degradation rate needs control	Drug delivery, tissue engineering, implantable devices, structural reinforcement
Conjugated polymers	π–π stacking; tunable optoelectronic and charge transport properties (e.g., P3HT, OPV, PCPV, PF); formation of highly ordered nanostructures	Crystallization and π–π interactions can compete; structural defects can affect charge transport	Organic electronics, photovoltaics, light-emitting devices, sensors, photocatalysis
Liquid crystalline polymers	Long-range order; anisotropic growth; stimuli-responsive (e.g., PBLG, PAChol, PFO, PAzo); highly tunable hierarchical organization	Sensitive to processing conditions; controlling long-range ordering in solution can be challenging	Optical materials, photonic devices, displays, responsive nanostructures, shape memory materials
Other crystallizable polymers	Broad material scope; diverse morphologies (e.g., PE, PEO, PAN, PFTMC, peptide-based); biologically relevant and industrially scalable options	Crystallization mechanisms can be complex and poorly understood in some systems	Biomedical applications, biomaterials, mechanical materials, nanocomposites, nanomedicine, protein mimetics

#### 2.2.2 Corona Chemistry

In CDSA, the corona-forming block plays a critical role in directing self-assembly, ensuring colloidal stability, and influencing morphological evolution.^310^ As the solvophilic segment, the corona prevents aggregation, enhances dispersion, and maintains the long-term stability of CDSA-derived nanostructures.^55^ Unlike conventional amphiphilic self-assembly, where solvent selectivity is the primary determinant of morphology, CDSA is predominantly controlled by the crystallization of the core-forming block, while the corona exerts a secondary yet essential influence on the assembly pathway, kinetics, and thermodynamics.^1^

The length, composition, and molecular architecture of the corona significantly affect the crystallization dynamics and morphology of CDSA assemblies.^271^ A longer corona-forming block sterically hinders unimer addition, slowing crystallization and favoring smaller, more stable platelets or fiber-like micelles. Conversely, a shorter corona reduces steric hindrance, accelerating crystallization and often leading to larger, more extended structures with enhanced core-chain packing.^150^ The corona also modulates dynamics of epitaxial growth, impacting nucleation efficiency and growth uniformity. Additionally, the corona-to-core ratio dictates morphology selection.^21^ A high corona-to-core ratio generally favors 1D fiber formation due to increased steric repulsion, while a lower ratio promotes 2D platelet structures by facilitating denser core packing.^311^ Tailoring this ratio allows controlled nanostructure formation, offering new avenues for designing hierarchical self-assemblies.

A broad spectrum of corona-forming polymers has been employed to tune interfacial interactions and functional properties in CDSA, including polysiloxane-based,^312–314^ polyacrylate-based,^130,139,315–317^ polyolefin-based,^318–321^ polyvinylarene-based,^48,113,322,323^ polyacrylamide-based,^124,324,325^ conjugated polymer-based,^40,190,228,326^ charged end-group,^327^ and other functional coronas^227,328,329^ (Figure 2). Their design influences not only colloidal stability but also responsiveness and compatibility with postassembly modifications.

**Figure 2:**
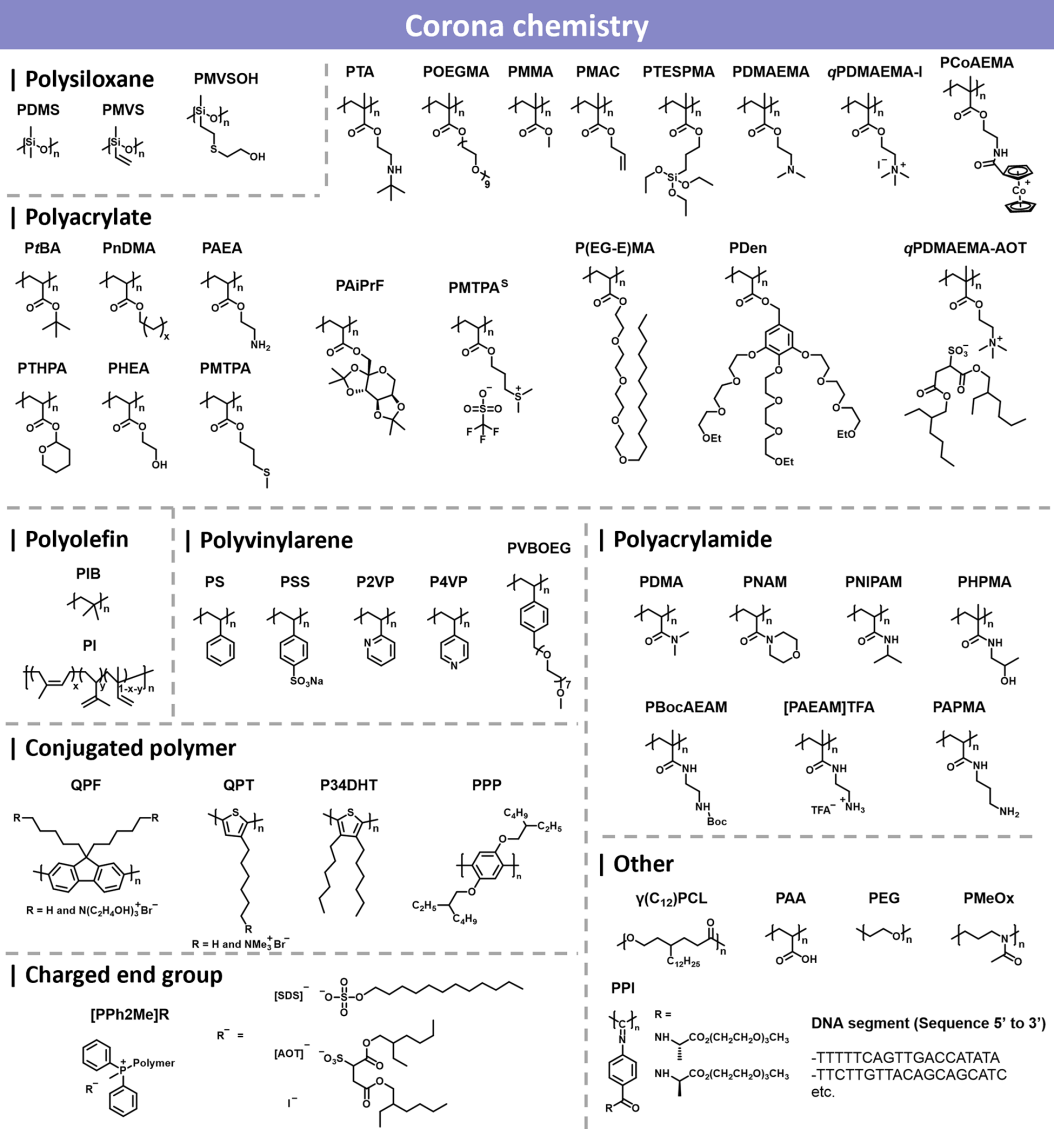
Overview of different corona chemistries used in CDSA.

The functional diversity of coronas enables postassembly modifications for tailored applications. For example, PEG-based coronas improve biocompatibility, while charged coronas facilitate electrostatic assembly of bioactive molecules. Stimuli-responsive coronas, such as PNIPAM, enable temperature-dependent assembly disassembly behavior, making them valuable for adaptive materials. The rational design of corona-forming blocks is crucial for advancing CDSA toward biomedical, electronic, and catalytic applications. Table 3 summarizes the typical advantages and limitations of commonly employed corona chemistries, serving as a practical guide for optimizing corona selection in CDSA systems. Moving forward, the strategic integration of functional coronas with precisely controlled corona-to-core ratios will further expand the utility of CDSA in creating responsive, hierarchical, and multifunctional nanomaterials.

**Table 3:** Summary of Typical Corona Chemistries in CDSA

**Corona chemistry**	**Advantages**	**Limitations**
Polysiloxane-based	High flexibility; low surface energy; thermal/chemical stability	Poor aqueous solubility; limited biocompatibility
Polyacrylate-based	Tunable hydrophilicity; good mechanical strength; versatile functionalization	Ionic sensitivity; may destabilize in high ionic strength environments
Polyolefin-based	Good chemical resistance; hydrophobic stabilization	Low corona dynamics; slow unimer exchange
Polyvinylarene-based	Good solvent compatibility; rigid corona for stable morphology	Limited water solubility; may reduce flexibility in dynamic systems
Polyacrylamide-based	Strong hydrogen bonding; structural rigidity; enhanced stability	May hinder dynamic assembly; lower solubility
Conjugated polymer-based	Optical/electronic functionality; π–π stacking for energy/electron transport	Potential aggregation; reduced colloidal stability without careful design
Charged end group	Electrostatic stabilization; functionalizable for bioconjugation	Highly sensitive to ionic strength and pH fluctuations
Other	PEG based: High biocompatibility; stealth behavior; flexible chain dynamics	PEG based: Limited stimuli-responsiveness; may lead to corona collapse at high temperature

#### 2.2.3 Morphology Control

CDSA provides controlled nanostructure morphology, enabling the fabrication of well-defined 1D, 2D, and hierarchical architectures.^17^ The final morphology is governed by multiple factors, including polymer composition, solvent conditions, crystallization kinetics, and seeded growth strategies.^5^ By fine-tuning these parameters, nanostructures with controlled dimensions, segmented structures, aspect ratios, and hierarchical complexity can be achieved, expanding the applicability of CDSA in functional materials.^330^

##### 2.2.3.1 1D Nanostructures

CDSA enables the formation of cylindrical micelles and nanofibers with tunable length and directional assembly. Through controlled growth, discussed in the following section, the preparation of uniform micelles with predictable dimensions can be achieved.^5^ This method has been widely employed to fabricate noncentrosymmetric cylindrical micelles exhibiting directional assembly and gradient compositions along the fiber axis.^34^ Furthermore, morphology control has been demonstrated through variation of polymer composition, seed concentration, crystallization temperature, and solvent selection, allowing for the modulation of micelle length and diameter. The length distribution of 1D micelles is typically described using the ratio of weight-average to number-average length (*L*_w_/*L*_n_), analogous to the dispersity (*Đ*) in living polymerization. The relationship between *L*_w_/*L*_n_ and the standard deviation (σ) of micelle length has been derived by the Manners and Winnik groups,^87^ where it is shown that the best achievable distribution follows a Poisson distribution ( $\frac{L_{w}}{L_{n}}-1={\left(\frac{\sigma }{L_{n}}\right)}^{2}$ ). Importantly, even for micelles with *L*_w_/*L*_n_ is around 1.1, the corresponding standard deviation remains significant (e.g., ∼32 nm for 100 nm rods), underscoring the inherent limitations in achieving extremely narrow length distributions. This awareness is critical when evaluating morphology control in living CDSA systems. Additionally, 1D nanostructures serve as fundamental building blocks for hierarchical assembly, leading to more complex morphologies such as dendritic micelles,^331^ bundled, branched, or isolated nanofibers,^332^ further broadening the versatility of CDSA.

##### 2.2.3.2 2D Nanostructures

Beyond 1D assemblies, CDSA facilitates the fabrication of 2D nanostructures with controlled lateral dimensions, aspect ratios, and layer organization.^333,334^ Optimizing crystallization parameters enables the formation of high-aspect-ratio platelets with well-defined edges,^327^ providing stable anisotropic assemblies suited for optical and electronic applications. Morphologically tunable square and rectangular nanosheets,^335^ and hexagonal platelets,^324^ further enhance the structural diversity of CDSA-derived materials. Advanced gradient and terraced nanostructures,^47^ and spirangle nanostructures,^336^ highlight the potential of CDSA to generate functionalized 2D nanostructures with tailored properties.

##### 2.2.3.3 Hierarchical Structures

The living nature of CDSA allows for epitaxial seeded growth, leading to hierarchical architectures with controlled organization. By directing growth dynamics, CDSA has enabled the construction of intricate morphologies such as multiarmed micelles,^337^ butterfly-like assemblies,^338^ and supermicelles with tailored architectures.^314,339,340^ Hierarchical self-assembly has also been demonstrated in carbon nanotube hybrid mesostructures,^341^ and multidimensional nanoporous superstructures.^31,342,343^ Furthermore, CDSA has enabled the fabrication of architecturally sophisticated nanostructures, including hollow rectangular platelets,^344^ barbed and branched mesostructures,^345^ and more exotic assemblies such as toroidal micelles,^346^ multi-tori mesostructures,^347^ and spherulite-like micelles.^348–351^ These advancements underscore the ability of CDSA to construct complex, functionalized nanomaterials, positioning it as a powerful platform for practical applications.

## 3 Mechanisms Understanding of the CDSA Process

### 3.1 General Synthetic Strategy of CDSA

If one of the blocks is crystallizable, such as PFS, PCL or PLA, crystallization can serve as a driving force that supersedes solvophilic and solvophobic interactions during self-assembly. This process is known as CDSA.^1,2,5,352^ Through CDSA, anisotropic structures such as cylinders and platelets—which are difficult to achieve through solvent-switching—can be easily obtained, as crystallization inherently favors structures with low curvature. In the following, we will review different CDSA strategies to prepare nanoparticles in terms of their mechanisms, operations, and controllability.

#### 3.1.1 Direct CDSA

CDSA can be categorized into two types based on the method and control over nanostructure size: direct CDSA and living CDSA.^2,5^ In direct CDSA, unimers first form and then undergo crystallization. A common approach involves heating a supersaturated polymer solution until all polymers dissolve, forming a unimer solution. Upon cooling, polymers precipitate, and CDSA occurs simultaneously (Figure 3a). Another method is solvent switching, where the polymer is dissolved in a good solvent, followed by the addition of a poor solvent to trigger CDSA. The Chen^110,117,353,354^ and Xu^116,355–357^ groups conducted a large volume of solvent-switching work on PCL-based block copolymers, where they normally used tetrahydrofuran (THF) or dimethylformamide (DMF) to dissolve polymers, and introduced methanol or water as the poor solvent to drive CDSA (Figure 3b). The choice and nature of the poor solvent significantly influence the resulting morphology.^357,358^ For instance, Wang et al. showed that 2D platelets formed from PCL-*b*-PDMAEMA in DMF converted to 1D cylinders upon the addition of just 0.74% v/v n-hexanol—a minor change in poor solvent composition.^357^ Similar solvent-driven morphological transitions were reported by Rizis, van de Ven, and Eisenberg.^358^ A more detailed discussion of solvent effects is provided in the following sections.

**Figure 3:**
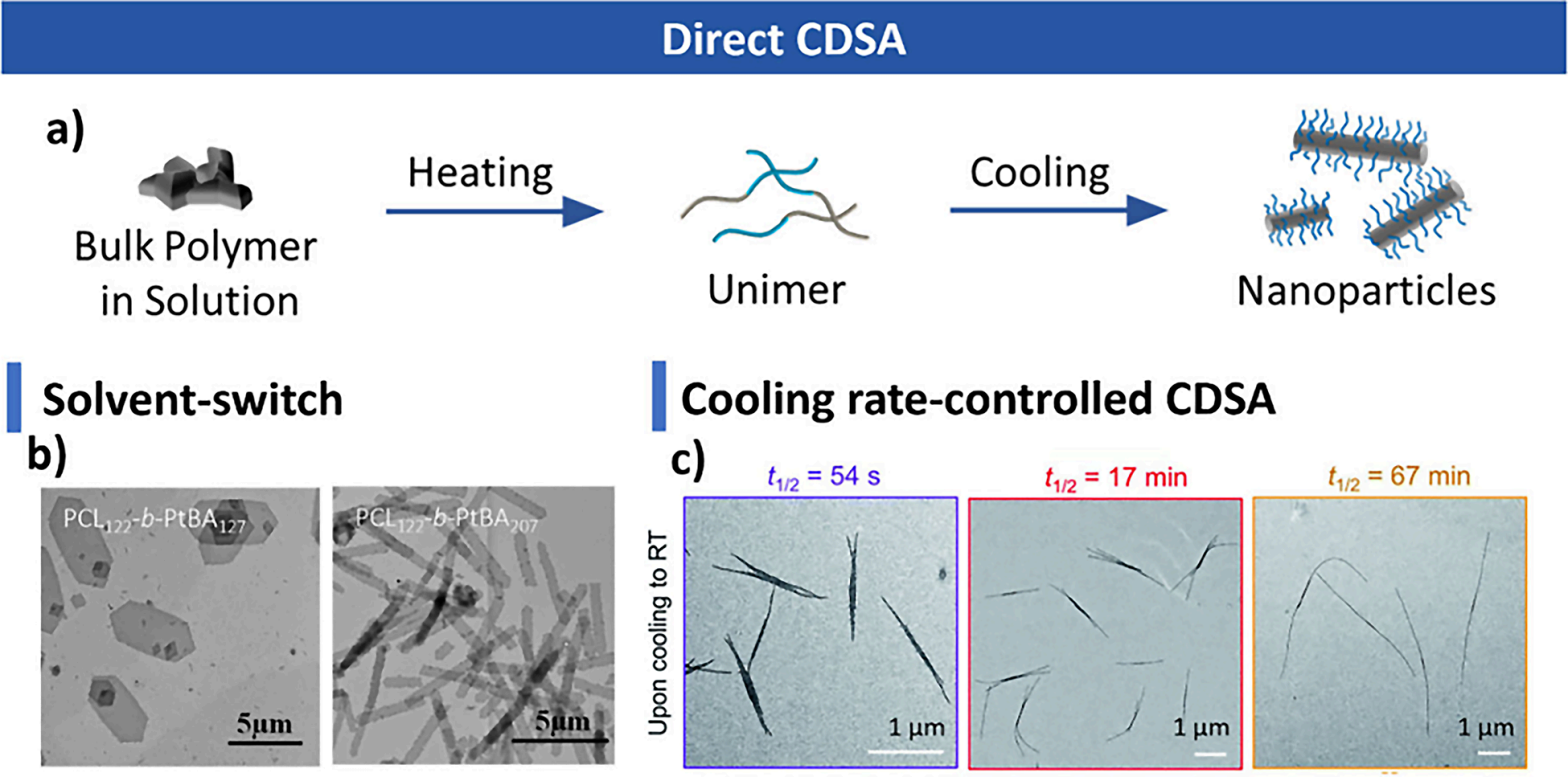
Direct CDSA. (a) Temperature-regulated direct CDSA process. (b) Nanostructures formed via solvent-switched direct CDSA. Reproduced with permission from ref 354. Copyright 2023 Elsevier. (c) Nanostructures obtained by direct CDSA under different cooling rates. Reproduced with permission from ref 359. Copyright 2022 Royal Society of Chemistry.

However, controlling the size and morphology of nanostructures using direct CDSA is challenging, as nucleation and epitaxial growth occur simultaneously, making it difficult to regulate both processes. Previous studies have shown that polymer crystallization rate, influenced by cooling rate or the solvophobic strength of the poor solvent, significantly impact the resulting nanostructures.^289,359^ For instance, Song et al. demonstrated that in PFS-based block polymers, rapid cooling induced instant supersaturation, favoring nucleation and resulting in short, branched cylinders, whereas slow cooling promoted epitaxial growth, yielding long, well-defined cylinders (Figure 3c).^359^ To leverage this, trace amounts of PFS homopolymer, which crystallizes more readily and nucleates earlier than the BCP, were added to a cylinder-forming PFS BCP, enabling the formation of monodisperse cylinders.^313^ Conversely, excessively fast cooling can cause rapid precipitation, even hindering crystallization and resulting in an amorphous core with reduced structural stability.^360^

#### 3.1.2 Seeded Growth

To achieve better control over the morphology and size of the nanostructures, living CDSA was developed to separate the phases of nucleation and epitaxial growth. To prepare nuclei seed particles, diblock copolymers are initially used to prepare dispersed cylinders through direct CDSA, which are then fragmented via sonication to generate uniform seeds (Figure 4a).^38,114,130^ Due to the low crystallinity of diblock polymers, the formation of these cylinders can be a time-consuming process, often taking several days. In addition, the quality and state of seed particles significantly influence epitaxial growth and, consequently, the morphology of the resulting nanostructures. For instance, Tong and co-workers found that seed particles can become deactivated when combined with metal ions, which inhibit the initiation of epitaxial growth.^361^ Moreover, Qiu and co-workers demonstrated that immobilizing seeds on a surface can lead to asymmetric growth, yielding unilateral platelets.^362^

**Figure 4:**
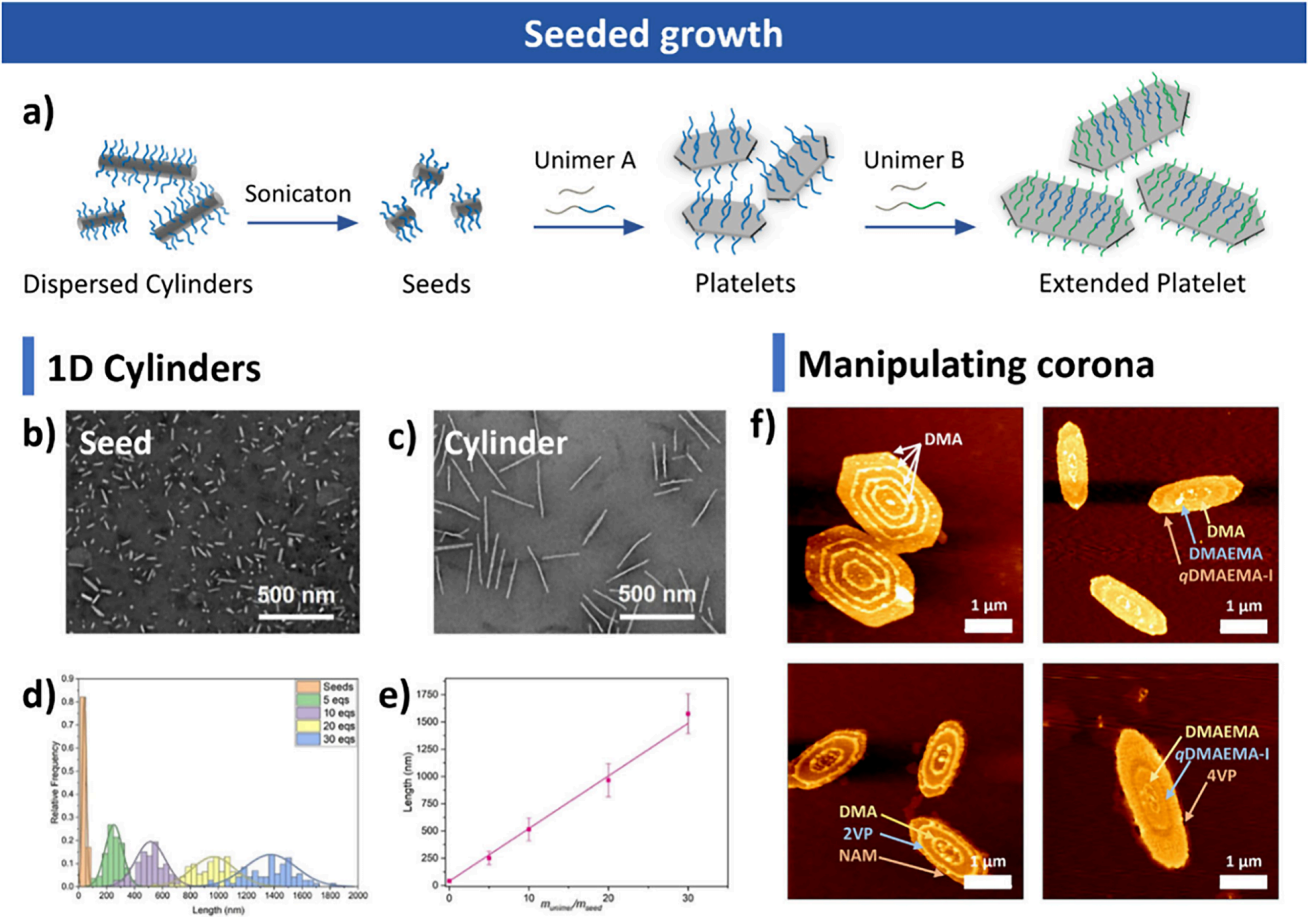
Seeded growth. (a) Schematic of platelet formation via seeded growth. (b, c) TEM images of seeds and cylinders formed by seeded growth. (d, e) Linear growth of cylinders with increasing unimer amount. Reproduced with permission from ref 305. Copyright 2022 American Chemical Society. (f) Segmented PCL-based platelets with different coronas. Reproduced with permission from ref 124. Copyright 2023 American Chemical Society.

Following the preparation of seeds, the growth process can be initiated through two primary methods: seeded growth and self-seeding. In the seeded growth approach, an additional unimer solution (polymer dissolved in a good solvent) is added to the seed solution to drive epitaxial growth. This method allows for the production of uniform nanostructures, with their size exhibiting a linear relationship with the amount of added unimer, making it practical to control size by adjusting the unimer-to-seed ratio. Besides, the final morphology of the nanostructures can be tuned according to the composition of the unimer solution. Reports indicate that 1D cylinders are formed when only diblock polymers are fed to the seeds, while the synthesis of 2D platelets necessitates a mixture of homopolymers and diblock polymers. Eisenberg and colleagues conducted a detailed study on the effects of homopolymer additives and found that increasing the ratio of homopolymer leads to a morphological transition from cylinders to platelets.^358^ In addition, seeded growth enables linear control of nanostructure size with the amount of added unimer, providing a basis for size regulation during epitaxial growth (Figure 4b–e).^305^ Furthermore, unimers with the same core block but different coronas or end groups can be used to prepare hybrid structures. For example, the O’Reilly and Dove groups synthesized PCL-based platelets by sequentially adding unimers with varying corona chemistries to seed solutions, resulting in diverse surface patterns on the platelets (Figure 4f).

#### 3.1.3 Self-Seeding

Another living CDSA strategy is self-seeding (Figure 5a). In this process, a seed solution is first annealed at an elevated temperature, where low-crystallinity, defective seeds melt to form unimers, while high-crystallinity seeds remain stable and survive. During subsequent cooling, the newly formed unimers epitaxially grow on the remaining seeds, leading to uniform extended nanostructures. A variation of this method, solvent-induced self-seeding, involves partially dissolving the seeds by adding a volatile good solvent. Upon solvent evaporation, the remaining seeds undergo regrowth, producing well-defined nanostructures.^363^ Although self-seeding is a practical method for producing uniform particles, its size control is less consistent compared to seeded growth. This is due to the many factors affecting seed survival after annealing, such as temperature, annealing time, and concentration, making it difficult to establish a linear relationship between the final size of the nanomaterials and any single factor. For example, Choi and co-workers used a π-conjugated polymer to prepare uniform nanofibers via self-seeding (Figure 5b), observing that while nanofiber length increased with temperature, the relationship was not linear (Figure 5c).^175^ While self-seeding is commonly used to prepare uniform cylinders, reports also demonstrate the preparation of uniform platelets using this method (Figure 5d).^102^

**Figure 5:**
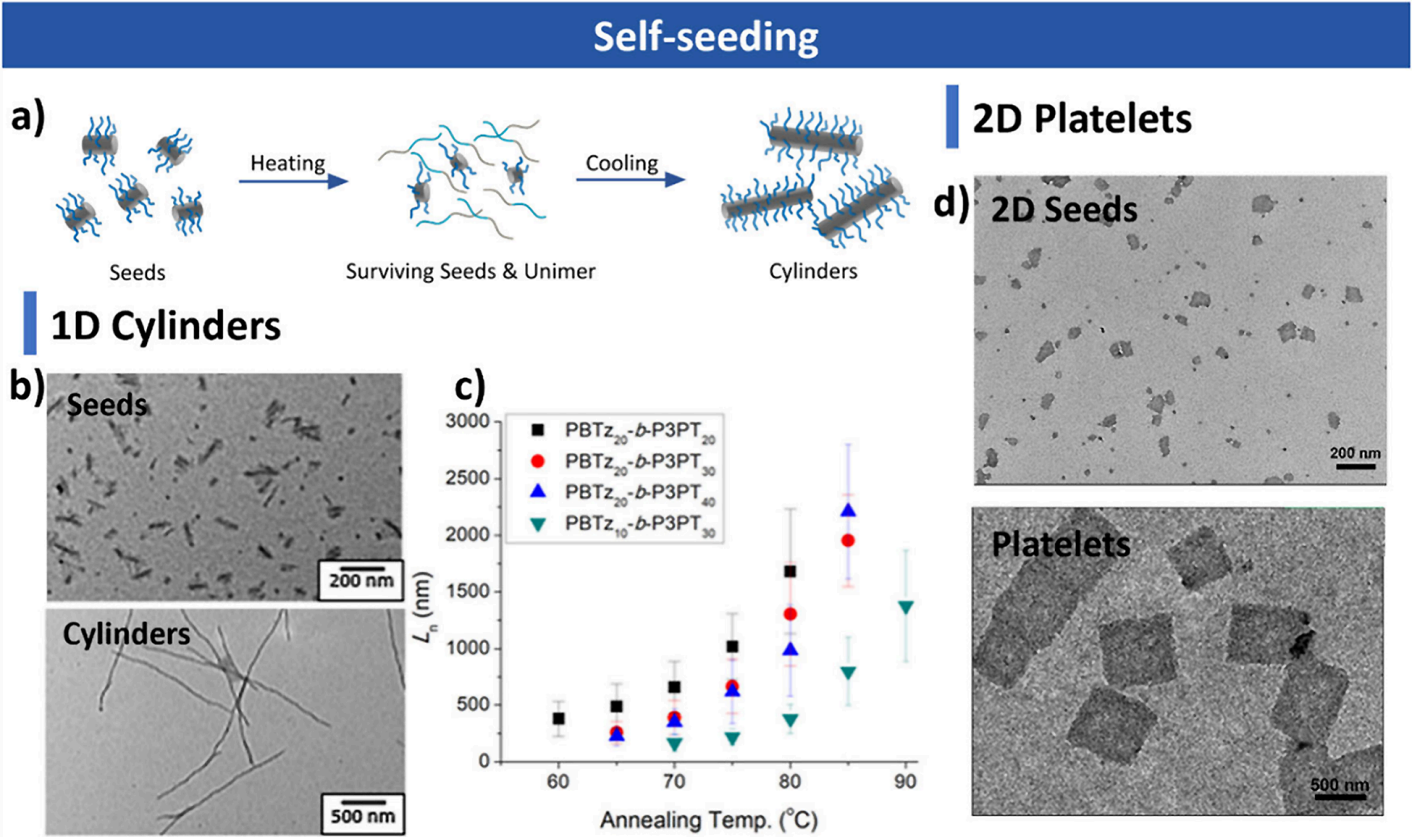
Self-seeding CDSA. (a) Schematic of the self-seeding process. (b) TEM images of seeds and cylinders formed by self-seeding. (c) Cylinder length as a function of annealing temperature. Reproduced with permission from ref 175. Copyright 2024 American Chemical Society. (d) TEM images of 2D seeds and platelets formed by self-seeding. Reproduced with permission from ref 102. Copyright 2022 American Chemical Society.

#### 3.1.4 Polymerization-Induced CDSA

To enable the commercial application of nanoparticles prepared via CDSA, scalable production is crucial. However, living CDSA requires dilute solutions (typically less than 0.1 wt %) for reliable size control and uniformity, which limits throughput. Additionally, sonication used for seed particle preparation is restricted to small-scale production due to the limited propagation of ultrasonic energy. These factors make scaling up CDSA nanoparticle production a significant challenge.

Inspired by polymerization-induced self-assembly (PISA), where polymerization and self-assembly occur simultaneously at high concentrations, polymerization-induced crystallization-driven self-assembly (PI-CDSA) was introduced to enhance throughput (Figure 6a). While some studies have increased production, they often compromised nanoparticle size control, leading to high dispersity (Figure 6b).^127,145,319^ To address this, the Manners group introduced preformed seed particles into the PFS-based PI-CDSA system (living PI-CDSA), separating nucleation from epitaxial growth to better control cylinder size (Figure 6c–e), yielding uniform cylinders with the length determined by unimer-to-seed ratio.^297^ However, there are few reports of uniform 2D structures, possibly because 2D platelet formation typically requires mixtures of homopolymers and block copolymers, which complicate the already rapid PI-CDSA process. For instance, Reuther and colleagues have successfully produced uniform platelets using poly­(aryl isocyanide) (PAIC) in PI-CDSA, though size control was inferior to traditional methods.^336^ Consequently, scaling up CDSA remains challenging, and new, more efficient techniques are needed.

**Figure 6:**
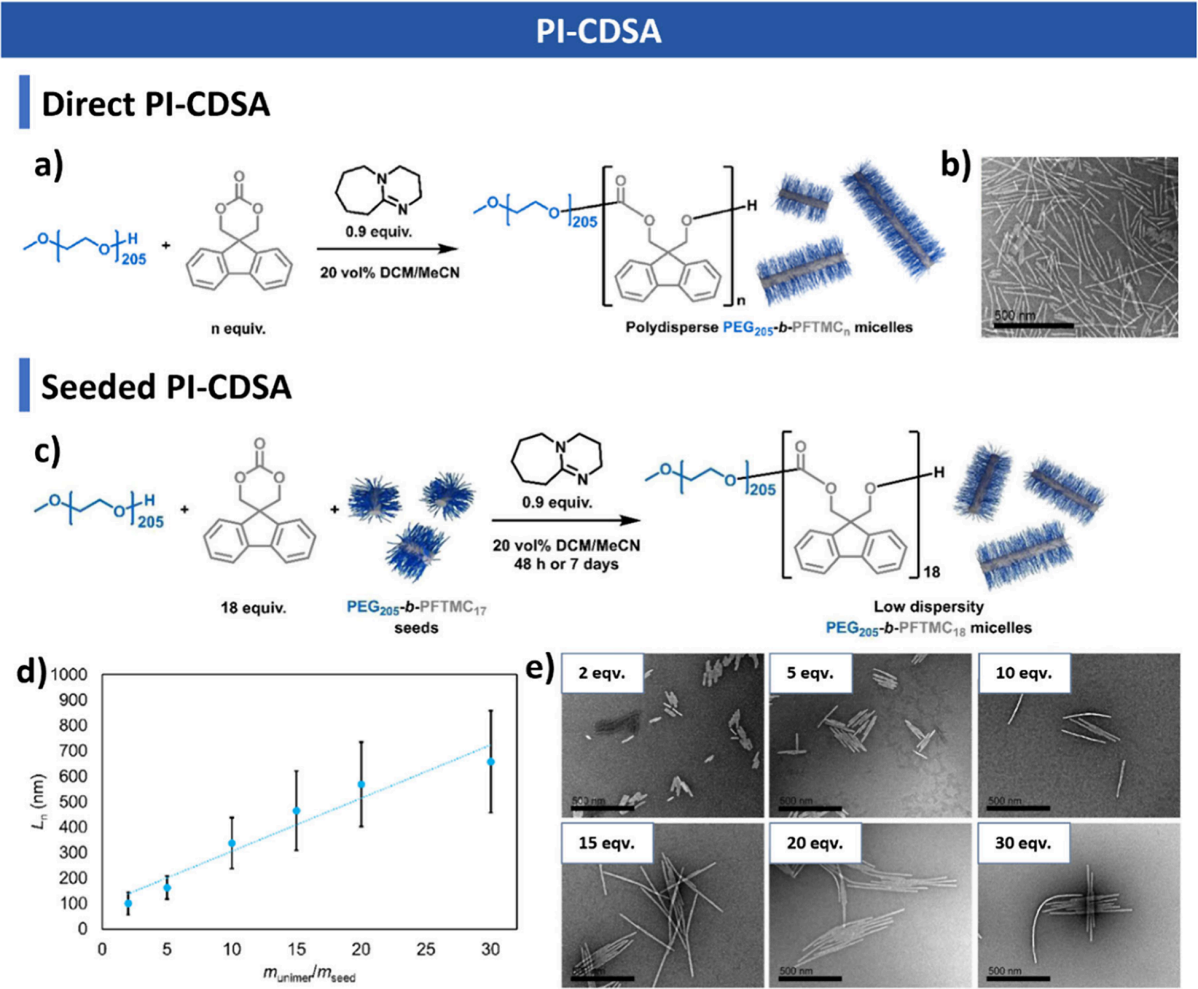
PI-CDSA. (a) Schematic illustration of PI-CDSA. (b) Polydisperse cylinders formed by PI-CDSA. (c) Seeded PI-CDSA process for uniform cylinders. (d) Linear growth of cylinders with increasing unimer-to-seed ratio. (e) TEM images of cylinders at different unimer amounts. Reproduced with permission from ref 297. Copyright 2022 American Chemical Society.

#### 3.1.5 Other Approaches

Besides the above-mentioned general methods, there are also some derivatives that apply external stimuli as a source to induce CDSA. Using a redox reaction as the stimulus, the Winnik and Manners groups oxidized noncrystallizable Fe^2+^ PFS species into Fe^3+^ crystallizable species, and therefore CDSA would occur spontaneously to form spheres or fibers. They also demonstrated that the strategy was reversible, that preprepared structures would disassemble to unimer once Fe^3+^ was reduced to Fe^2+^.^364^ This redox-mediated approach was later extended to π-conjugated block copolymers by the Seferos group, where selective oxidation of the core block promoted the formation of uniform nanoparticles.^365^ More recently, they applied this strategy to π-conjugated homopolymers (Figure 7a). By tuning the concentration of the oxidative dopant, iron­(III) p-toluenesulfonate (FeTs_3_), and the assembly temperature, they accessed three distinct morphologies. Additionally, the dopant’s counterion contributed to colloidal stability of the resulting nanoparticles.^366^ Alternatively, the Choi group modulated the crystallization ability of poly­(p-phenylenevinylene) blocks by cis-to-trans photoisomerization (Figure 7b–d), where the living growth behavior can be precisely controlled by switching the light on and off.^367^ In addition to tuning polymer structures, Sun and co-workers used temperature as the stimulus to control crystallization of the same polymer (Figure 7e), where kinetically trapped spherical nanoparticles at a lower temperature would transform to a thermodynamically stable cylindrical morphology upon heating, as increased temperatures favored chain rearrangement to crystallize. As such, this process was irreversible.^256^ With the ongoing pursuit of improved control over nanostructures, it is anticipated that more diverse and innovative methods will be developed in the future, further expanding the toolbox for controlled CDSA.

**Figure 7:**
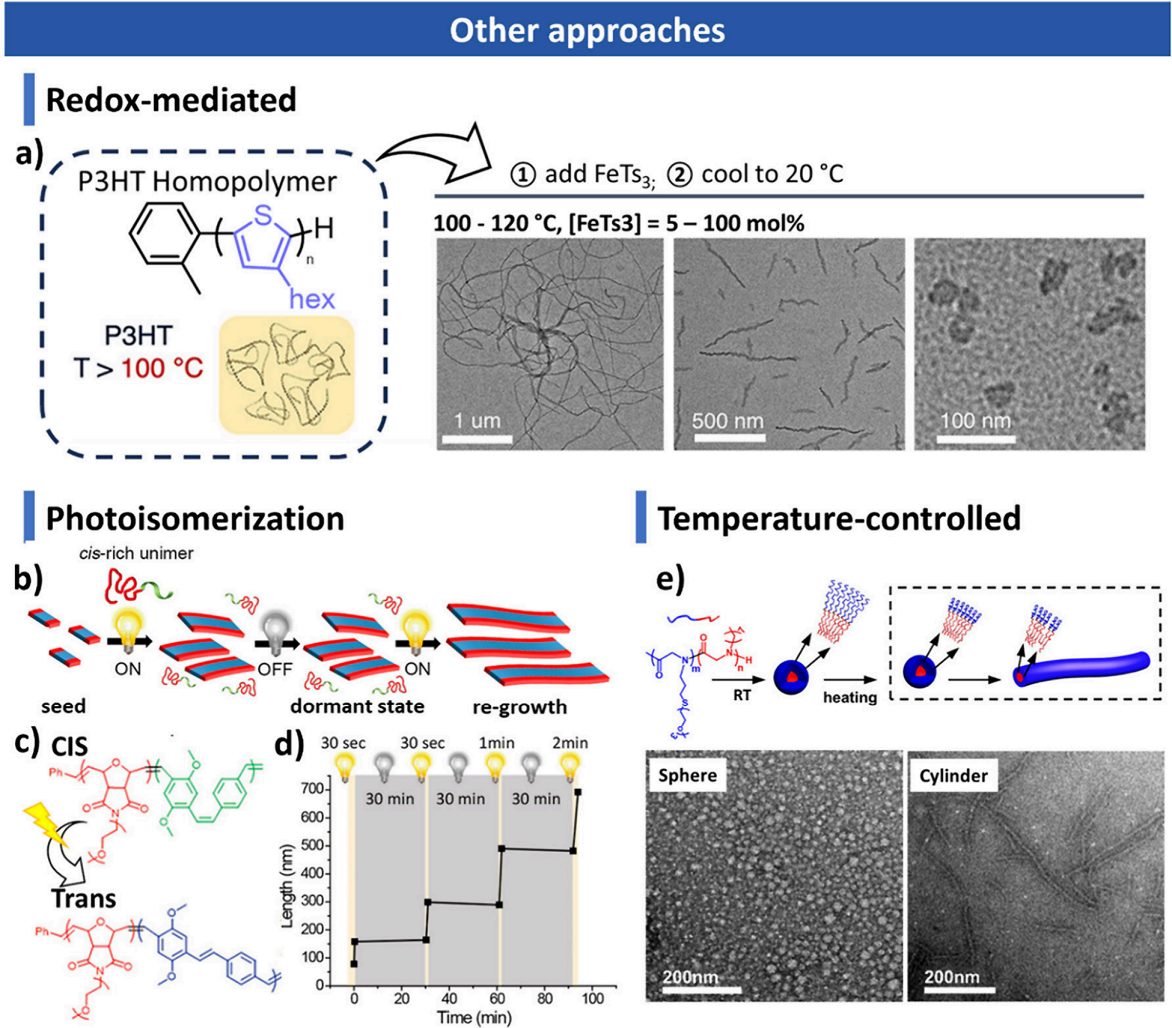
Other approaches for CDSA. (a) Redox-mediated CDSA of poly­(3-hexythiophene) homopolymer. Reproduced with permission from ref 366. Copyright 2022 American Chemical Society. (b) Scheme of living CDSA driven by photoisomerization. (c) Molecular structures of BCPs before and after photoisomerization. (d) Plot of fiber length versus time for light on–off cycles. Reproduced with permission from ref 367. Copyright 2018 American Chemical Society. (e) Temperature-controlled morphological transition through CDSA. Reproduced with permission from ref 256. Copyright 2020 American Chemical Society.

### 3.2 Factors Influencing CDSA

CDSA enables the fabrication of well-defined nanostructures with controlled size, morphology, and segmental organization. However, the final characteristics of CDSA-derived assemblies—such as morphology, dimensions, uniformity, kinetics, and colloidal stability—are governed by multiple parameters, including block copolymer composition (block length and ratio), homopolymer presence, solvent conditions, temperature, end group functionalities, and additives. Understanding how these factors influence self-assembly is critical for rational design and optimization of CDSA systems.

#### 3.2.1 Blocks Length and Ratio

CDSA follows general self-assembly principles, such as core–corona stretching and core–solvent repulsion, but is uniquely dictated by crystallization ability. The length and ratio of the core-forming and corona-forming blocks significantly influence self-assembly behavior, determining final morphology and structural organization.^54,116,150,225,268,353,354,368–374^

For example, Sleiman and co-workers demonstrated that subtle changes in the length of the hydrophobic block, composed of branched C8 alkyl chain-based monomers (bC8), led to unexpected morphological transitions.^311,375^ Cryo-TEM analysis of bC8_8_-DNAa assemblies revealed that increasing the hydrophobic block by just four monomers (from bC8_8_ to bC8_12_, the subscript indicates the degree of polymerization) induced a transition from long to short fibers (Figure 8a). This was attributed to core compaction, where alkyl chains reorganized within the fiber core, while the phosphate backbone wrapped around them, influencing self-assembly outcomes.

**Figure 8:**
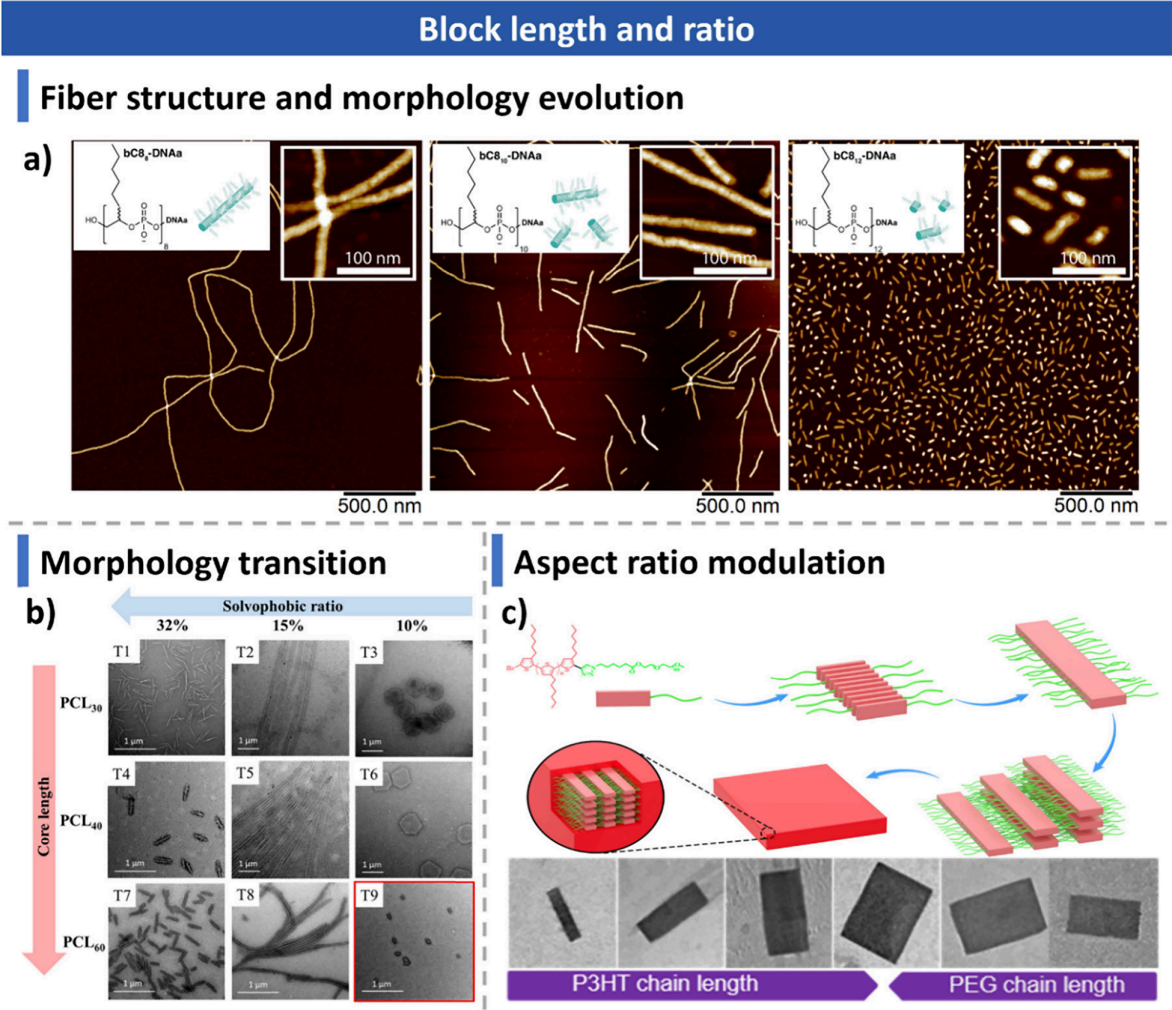
Effect of block length and block ratio on CDSA morphology. (a) Fiber length variation in bC8_10_-DNAa, bC8_12_-DNAa, and bC8_8_-DNAa assemblies. Reproduced with permission from ref 311. Copyright 2024 Springer Nature. (b) Morphology transition of triblock copolymers. TEM images of micelles formed from PDMA_*x*_-*b*-PCL_*y*_-*b*-PDMA_*x*_ in methanol after 2 days at room temperature. Reproduced with permission from ref 121. Copyright 2020 American Chemical Society. (c) Aspect ratio tuning in 2D rectangular micelles by adjusting P3HT and PEG block lengths. Reproduced with permission from ref 194. Copyright 2020 American Chemical Society.

O’Reilly, Dove, and co-workers reported a cylinder-to-platelet transition in PLLA-*b*-PDMA diblock copolymers upon increasing the corona-to-core block ratio.^150^ A similar trend was observed in PDMA-*b*-PLLA-*b*-PDMA triblocks, where longer hydrophilic coronas and more soluble unimers reduced crystallization rates, favoring 2D platelet formation.^141^ However, the Wooley group observed the opposite trend when using α-d-glucose carbonate (DGC) as the corona block.^99,169^ At a low corona-to-core ratio (1:2), bundle-like cylinders and platelets formed possibly due to insufficient coronal repulsion, while increasing the ratio to 1:1 led to cylinders, and 4:1 resulted in spherical assemblies.

In PCL-based triblock copolymers (PDMA-*b*-PCL-*b*-PDMA), corona length dictated morphology. A longer corona promoted the formation of 2D hexagonal platelets, whereas a shorter corona led to elongated fibers (Figure 8b). This may result from increased polymer solubility, allowing semicrystalline chains to self-organize into their preferred crystalline conformations.^121^

Chen et al. investigated PCL-*b*-PDMAEMA platelet assemblies, showing that longer PDMAEMA coronas produced smaller, less uniform platelets possibly caused by increased defect-induced chain folding outside the PCL crystal plane, disrupting structural order.^353^ A similar trend was observed in PCL-*b*-PAA BCPs, where increasing the PAA block length resulted in irregular platelet structures and aggregation.^110^

Block length also affects the aspect ratio in CDSA. He and co-workers examined P3HT-*b*-PEG assemblies and found that as the P3HT/PEG ratio increased, both fiber and 2D micelle lengths increased (Figure 8c). The widths of 2D micelles were dictated by the packing density of adjacent fibers, expanding with longer P3HT chains but contracting with longer PEG chains. Interestingly, the P3HT/PEG ratio had a stronger influence on width than on length, leading to decreased aspect ratios in 2D rectangular micelles with increasing P3HT content.^194^

These studies collectively demonstrate that block length and ratio critically govern CDSA morphology, uniformity, and aspect ratio, with hydrophobic core–hydrophilic corona interactions driving diverse structural transitions. Understanding these relationships enables controlled morphological tuning, facilitating tailored applications in nanomaterials design.

#### 3.2.2 Role of Homopolymers

The incorporation of crystalline homopolymers significantly influences the CDSA process, particularly in promoting the formation of 2D nanostructures. However, these assemblies often suffer from poor colloidal stability unless modified with hydrophilic substituents.^376^ Compared to systems composed solely of BCPs, homopolymer addition modulates morphology, accelerates assembly, and facilitates transitions from 1D to 2D structures.^241,377^ Notably, homopolymers reduce assembly time to mere minutes in a PCL system, offering promising opportunities for scalable CDSA processing.^378^

In 2014, Eisenberg, van de Ven and colleagues reported that increasing homopolymer content induces a cylinder-to-platelet transition.^358^ The Manners and Winnik groups later developed a seeded growth strategy to fabricate monodisperse rectangular platelets using a blend of PFS_36_-*b*-P2VP_502_ BCP and PFS_20_ homopolymer, growing from cylindrical micelle seeds.^344^ The sequential addition of homopolymers and BCP blends with different corona chemistries (P2VP or PDMS) enabled the formation of concentric rectangular platelet block comicelles, which could be selectively cross-linked to yield hollow rectangular platelets upon disassembly in a good solvent. Additionally, spatially defined dye-functionalized 2D micelles were fabricated and characterized via fluorescence microscopy. This work demonstrated unprecedented control over 2D morphology, providing a tunable platform for functional soft materials.

The topology of homopolymers plays a crucial role in CDSA behavior. Tong and co-workers synthesized tadpole-shaped PCL modified with styrylpyrene (SPy), which could be cyclized via [2 + 2] photocycloaddition.^379^ During living CDSA, platelets formed from cyclic PCL exhibited higher aspect ratios than those from tadpole-shaped PCL (Figure 9a,b), likely due to reduced crystallization ability in cyclic PCL, which altered the growth rates of the (110) and (200) crystalline planes, leading to distinct platelet dimensions.

**Figure 9:**
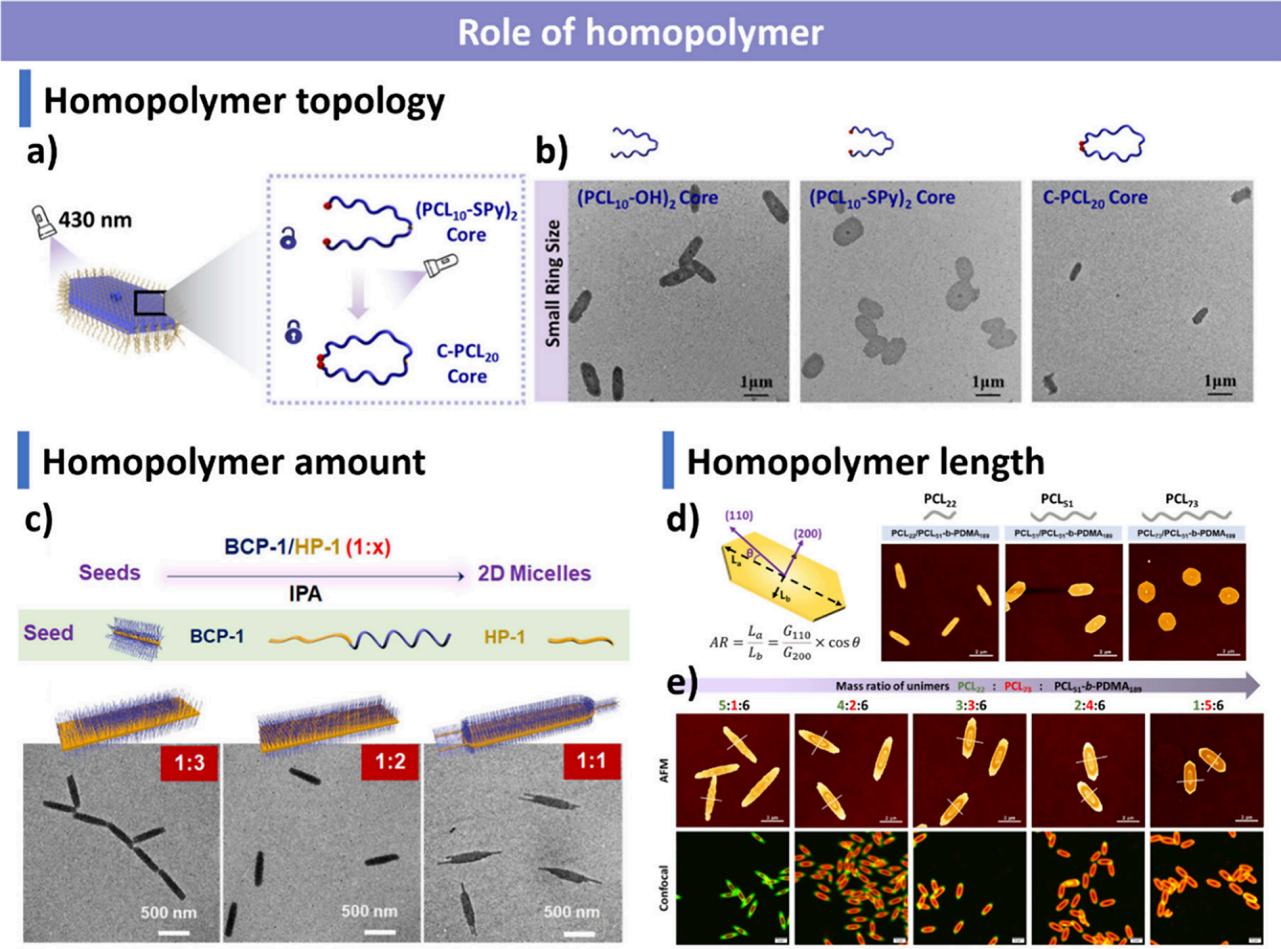
Role of homopolymer in CDSA. (a) Influence of homopolymer topology on 2D platelet formation via seeded growth under light irradiation. (b) TEM images of 2D platelets with core-forming blocks of (PCL_10_-OH)_2_, (PCL_10_-SPy)_2_, and C-PCL_20_. Reproduced with permission from ref 379. Copyright 2023 American Chemical Society. (c) Effect of homopolymer content on 2D micelle morphology at different BCP-to-homopolymer ratios (1:3, 1:2, 1:1). Reproduced with permission from ref 378. Copyright 2022 American Chemical Society. (d) Influence of homopolymer length (PCL_22_, PCL_51_, PCL_73_) on platelet aspect ratio. (e) AFM and confocal images of 2D platelets formed with mixed long and short PCL homopolymers. Reproduced with permission from ref 380. Copyright 2024 American Chemical Society.

The homopolymer-to-BCP ratio also impacts living CDSA morphology. An important early example of this effect was reported by the Winnik and Manners groups, they demonstrated that cocrystallization, along with the ratio of homopolymer to BCP, plays a crucial role in modifying the structure of micelles formed via living CDSA. In their study, the shape of PFS-*b*-PI block copolymer micelles was tuned by blending with a homopolymer. The nature of this morphological change was found to depend on the amount of homopolymer added.^321^ Manners and co-workers identified a competitive crystallization kinetics mechanism in PFS-*b*-P2VP/PFS homopolymer blends, where morphology was regulated by the molar ratio (c_B_/c_H_)_t_ and crystallization rates (G_B_/G_H_)_t_ (Figure 9c).^378^ Similarly, the O’Reilly and Dove groups demonstrated that increasing PCL homopolymer content relative to PCL-*b*-PDMA led to larger 2D platelets with greater area, length, and width.^124^ Additionally, the Winnik group demonstrated that the addition of trace amounts (<5 wt %) of PFS homopolymer to PFS-based block copolymers can significantly improve the uniformity of fiber-like micelles formed via direct CDSA.^313^ In this one-step protocol, the homopolymer preferentially crystallizes during the early cooling stage, acting as a nucleation seed for subsequent epitaxial growth of block copolymer unimers, to yield micelles with tunable lengths (∼0.6 to 9.7 μm) in selective solvents. This work highlights how small amounts of homopolymer can be used to synchronize nucleation and enhance control over micelle size and uniformity, providing a simple and scalable alternative to multistep seeded growth methods.

The length of homopolymers further dictates crystallization behavior. The O’Reilly and Dove groups synthesized PCL homopolymers of varying lengths for living CDSA and observed length-dependent crystallization rates that influenced platelet aspect ratios and spatial growth (Figure 9d,e).^380^ Longer PCL chains exhibited faster crystallization, uniformly overcoming energy barriers associated with both (110) and (200) planes, resulting in isotropic growth. In contrast, shorter PCL chains crystallized more slowly, favoring preferential growth along the (110) plane and yielding anisotropic platelets.

Homopolymers play a pivotal role in CDSA, influencing assembly morphologies, crystallization dynamics, and colloidal stability. Their presence facilitates 1D-to-2D transitions, accelerates assembly kinetics, and allows controlled morphological tuning through variations in topology, composition, and length. These findings highlight the versatility of homopolymers in tuning platelet properties, paving the way for tailored nanostructures with optimized aspect ratios and functionalization potential.

#### 3.2.3 Solvent

Solvent selection plays a critical role in governing self-assembly dynamics, crystallization behavior, and colloidal stability in CDSA. Selective solvation drives phase separation, controls nucleation and growth rates, and prevents aggregation by solvating the corona-forming block.^381,382^ Solvent properties such as polarity, miscibility, and solubility dictate assembly morphology, enabling transitions among micelles, cylinders, and platelets.^82,383^ By modulating thermodynamic parameters, solvent choice allows control over nanostructure size, shape, and uniformity, making it essential for tailoring CDSA assemblies.^320^

Solvent choice directly influences aspect ratios in 2D CDSA assemblies. The Choi group demonstrated that varying the cosolvent ratio in a conjugated PCPV system modulated nanorectangle aspect ratios.^335^ Adding good solvents (e.g., THF, chloroform) triggered a square-to-rectangular transition, with aspect ratio increasing linearly with cosolvent content (Figure 10a,b). This effect was attributed to solvent-driven variations in crystalline plane growth rates—with (110) > (010) > (100) surface energy—leading to anisotropic structures based on Wulff construction principles. Similarly, in PCL-based CDSA, the O’Reilly and Wallace groups found that increasing THF content enhanced unimer addition along the longer axis, yielding elongated platelets with higher aspect ratios.^384^ The improved polymer solubility in THF slowed overall growth while promoting preferential axial elongation.

**Figure 10:**
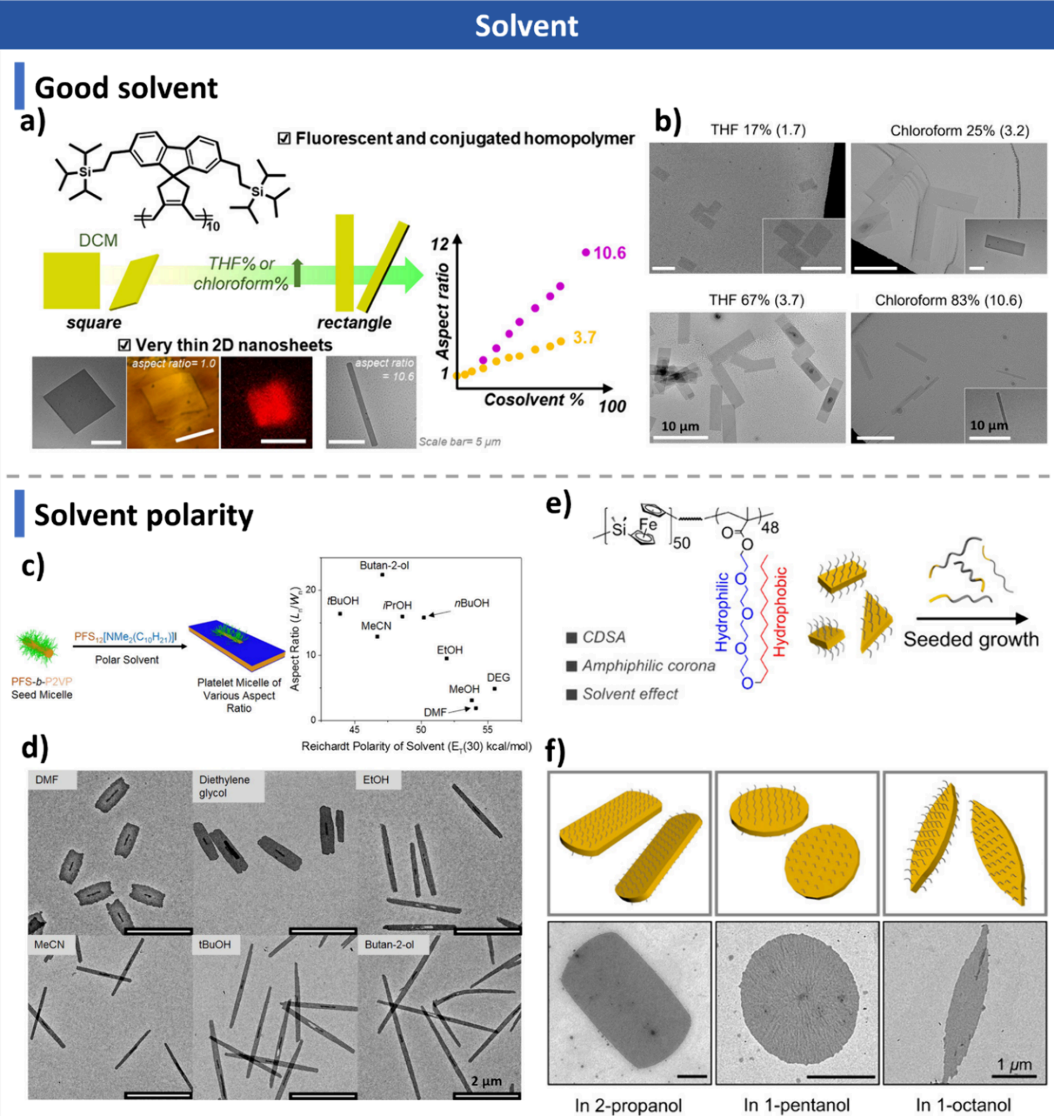
Influence of solvent on CDSA. (a) Effect of cosolvent (THF or chloroform) on 2D platelet aspect ratio. (b) TEM images showing square to rectangular nanosheet transition with increasing cosolvent content (scale bar: 10 μm). Reproduced with permission from ref 335. Copyright 2019 American Chemical Society. (c) Platelet aspect ratio control via seeded growth in solvents of different polarity. (d) TEM images of 2D platelets formed in solvents with varying polarity (scale bar: 2000 nm). Reproduced with permission from ref 327. Copyright 2019 American Chemical Society. (e) Amphiphilic corona-forming block promoting diverse 2D platelet morphologies. (f) TEM images of platelets formed in 2-propanol, 1-pentanol, and 1-octanol. Reproduced with permission from ref 382. Copyright 2021 American Chemical Society.

In contrast, poor solvents (assembly medium solvents) also impact aspect ratios. Manners and co-workers showed that tuning medium solvent polarity during PFS-based platelet seeded growth allowed control over aspect ratios from 2 to 20 (Figure 10c,d).^327^ A clear trend showed that higher medium solvent polarity resulted in lower aspect ratios, likely due to increased solvent–polymer interactions reducing anisotropic growth.

Solvent polarity also determines final assembly morphology. The Winnik group synthesized a PFS_50_-*b*-P­(EG-E)­MA_48_ block copolymer with amphiphilic pendant groups, producing distinct structures in different solvents (Figure 10e,f).^382^ In 2-propanol, rounded rectangular platelets formed, while 1-pentanol produced circular disks, and 1-octanol led to irregular, leaf-shaped structures. Interestingly, high unimer addition in 1-pentanol resulted in the formation of 3D circular spirals, likely driven by screw dislocations.

A similar solvent-dependent morphological transition was observed in a liquid crystalline system by Du et al., using poly­(3-(4-(phenyldiazenyl)­phenoxy)­propyl methacrylate) (PAzoPMA).^240^ The assembled structures varied with solvent polarity: cylinders formed in ethanol (high polarity), platelets in isopropanol, and large compound micelles in n-butanol (low polarity). These findings highlight how polymer–solvent interactions and intermolecular forces dictate unimer deposition and micelle growth, enabling solvent-driven morphology control.

Solvent selection is a key determinant in CDSA, influencing assembly kinetics, crystallization behavior, and final morphology. Solvent properties—including polarity, miscibility, and solvation ability—govern nucleation and growth rates, colloidal stability, and morphological transitions, allowing controlled tuning of aspect ratios and shapes. From rectangles and platelets to complex 3D spirals, studies demonstrate that solvent interactions provide an effective approach to modulate CDSA assemblies and influence their structural diversity. In addition to polarity, other intrinsic solvent parameters, such as hydrogen bonding capacity, dielectric constant, viscosity, and density, may also play important roles in modulating crystallization kinetics and morphology. However, systematic studies exploring the influence of these parameters, particularly in dominant solvents, remain limited and warrant further investigation.

#### 3.2.4 Temperature

Temperature is a key parameter in CDSA, governing crystallization behavior, assembly kinetics, and structural morphology across diverse polymer systems.^355,385^ Temperature modulation during polymerization, annealing, or cooling enables control over nucleation rates, growth kinetics, and defect formation, facilitating the fabrication of uniform and complex nanostructures. Temperature also facilitates hierarchical assembly, leveraging synergistic effects such as thermal responsiveness and crystallographic compatibility.^386^ Studies on various BCPs underscore temperature’s role in directing dynamic self-assembly processes and thermodynamic equilibrium, driving the formation of 1D, 2D, and 3D structures.^387^

Wang and co-workers demonstrated that polymerization temperature strongly influences the crystallization and self-assembly of PIB-*b*-PCL, PIB-*b*-PVL, and PIB-*b*-P­(CL-*co*-VL) systems.^319^ Nanoparticles with identical compositions formed distinct morphologies depending on polymerization temperature (Figure 11a). At 80 °C, PIB-*b*-PCL and PIB-*b*-PVL predominantly formed spherical micelles or precipitates, while at 20 °C, fibrillar micelles emerged due to temperature-dependent self-assembly pathways driven by crystallization. In PIB-*b*-P­(CL-*co*-VL), high polymerization temperatures favored random copolymer formation, leading to spherical micelles, whereas low polymerization temperatures yielded quasi-block copolymers, promoting fibrillar micelle growth.

**Figure 11:**
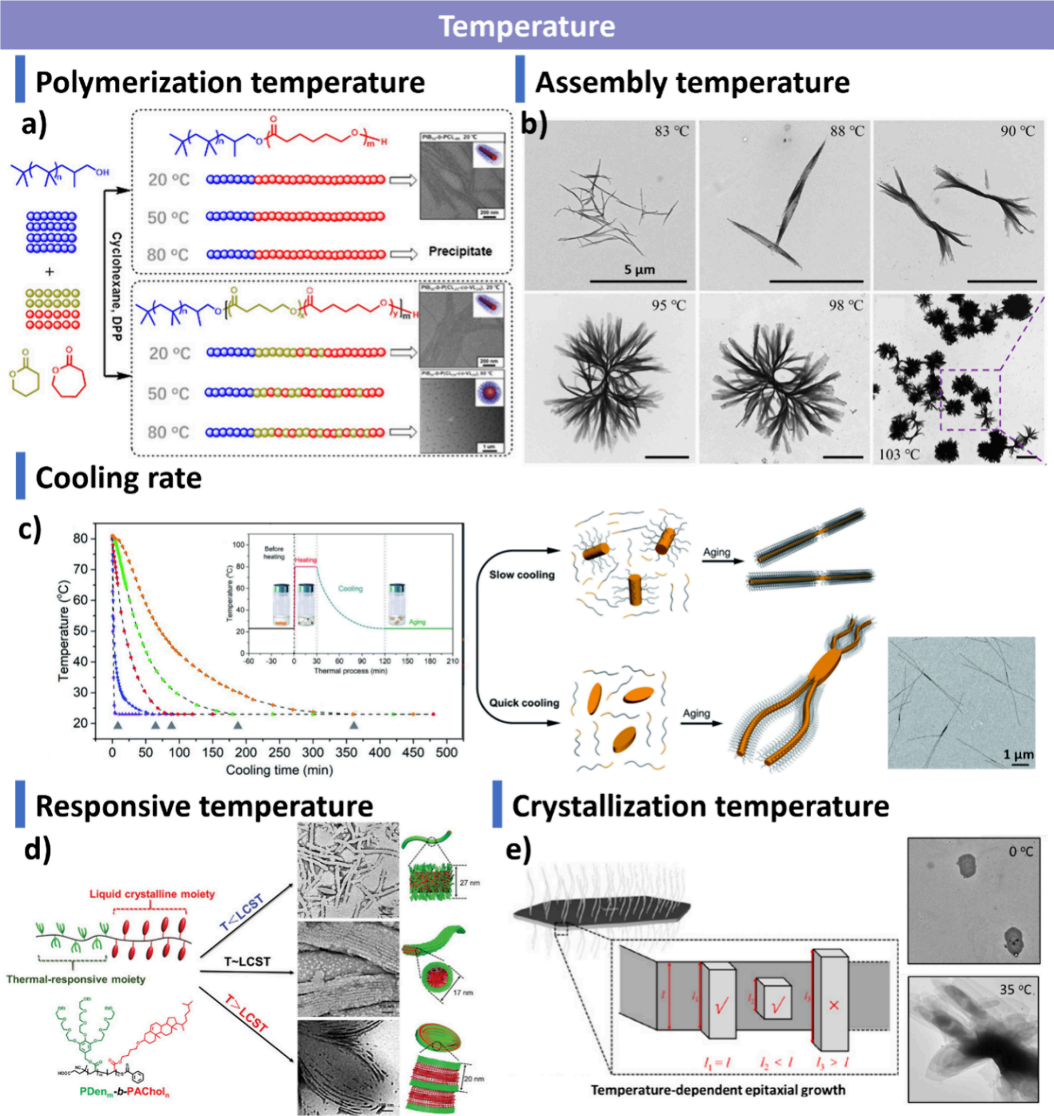
Influence of Temperature on CDSA. (a) ROPISA at different temperatures using PIB-OH as the stabilizer/macroinitiator with PCL, PVL, or P­(CL-*co*-VL) core-forming blocks. Reproduced with permission from ref 319. Copyright 2023 American Chemical Society. (b) TEM images of PFS_50_-*b*-PMMA_153_ micelles formed by heating in acetone at various temperatures, followed by cooling. Reproduced with permission from ref 349. Copyright 2023 American Chemical Society. (c) Co-self-assembly under different cooling conditions yielding distinct nanostructures. Reproduced with permission from ref 359. Copyright 2022 Royal Society of Chemistry. (d) Temperature-controlled self-assembly of PDen_14_-*b*-PAChol_36_, forming short fibers, bundled fibers, or multilayer vesicles at different temperatures. Reproduced with permission from ref 244. Copyright 2024 Wiley-VCH GmbH. (e) Seeded epitaxial growth regulated by crystallization temperature. Reproduced with permission from ref 388. Copyright 2023 American Chemical Society.

Winnik and co-workers developed a one-pot strategy for synthesizing uniform 3D structures via CDSA by modulating assembly temperatures, including heating, cooling, and aging conditions.^349^ Using PFS_50_-*b*-PMMA_153_ in acetone, they demonstrated a transformation from elongated lamellae to hedrites, sheaf-like micelles, and ultimately spherulites, driven by annealing temperature and supersaturation levels (Figure 11b). The formation of secondary crystals at defect sites on lamellae initiated growth, with subsequent multilayer branching and twisting leading to compact spherulites. This study highlights how controlled temperature conditions direct 3D structure evolution in CDSA.

The cooling rate significantly impacts nucleation, growth, and morphology in CDSA.^359^ The Winnik group showed that during the CDSA process, unimers dissolve upon heating and recrystallize as solubility decreases. Rapid cooling (cooling in air) generates high supersaturation, analogous to large undercooling in semicrystalline polymer melt crystallization. This promotes early nucleation of the least soluble components, forming seed particles with multiple faces that drive micelle growth. Faster cooling yielded highly branched structures (Figure 11c), whereas slower cooling (cooling in oil bath) allowed for more controlled growth. Notably, most self-assembly occurred after reaching room temperature, emphasizing that early cooling dynamics determine branching extent in CDSA.

Jia et al. introduced PDen-*b*-PAChol BCPs, where PDen (oligoethylene glycol dendron) provides a hydrophilic lower critical solution temperature (LCST)-responsive block and PAChol (cholesterol) forms a smectic liquid crystalline hydrophobic block.^244^ Upon heating, PDen blocks collapse due to LCST behavior, triggering assembly, while PAChol blocks direct epitaxial growth (Figure 11d). This synergistic combination of liquid crystalline ordering and thermal responsiveness enables controlled hierarchical self-assembly, distinguishing these BCPs from conventional thermoresponsive polymers.

Tong and co-workers examined crystallization temperature effects on seeded epitaxial growth of polymer blends, showing that lower temperatures promote epitaxial growth due to the lamellar thickness relationship between new and seed crystals.^388^ In studies of PHL epitaxial growth from PCL seed crystals (Figure 11e), they demonstrated that for epitaxial growth to occur, newly formed crystals must be thinner than the seed crystals, an energetically favorable condition. This work highlights crystallographic compatibility and thermal control as key factors in directing hierarchical polymer growth.

Temperature plays a pivotal role in CDSA, influencing crystallization, growth kinetics, and hierarchical structure formation. Morphology, aspect ratios, and structural complexity can be controlled by tuning polymerization, annealing, and cooling conditions. Studies on BCP crystallization, cooling dynamics, and hierarchical self-assembly underscore the versatility of temperature modulation in tailoring CDSA architectures. These insights provide a foundation for designing next-generation nanostructures with enhanced functionality and scalability.

#### 3.2.5 Additives

Additives—such as hydrogen bond disruptors, metal ions, and nanoparticles—play a crucial role in controlling assembly dynamics, crystallization behavior, and structural transitions in CDSA. By modifying intermolecular interactions, guiding nucleation, and facilitating morphology transformations between 1D, 2D, and 3D architectures, additives enhance uniformity, reduce defects, modulate solubility, and introduce functional properties such as conductivity and catalytic activity.^275,361^ Their ability to fine-tune assembly processes makes them valuable tools for tailoring CDSA structures for diverse applications.

He et al. demonstrated the controlled formation of uniform PLLA-based fiber-like micelles via living CDSA.^143^ A key requirement for efficient living CDSA is that added unimers elongate exclusively at seed micelle termini. However, in PLLA_47_-*b*-PNIPAm_267_, unimer aggregation and undesired self-nucleation occurred due to C–H···O hydrogen bonding between methyl protons and carbonyl oxygen atoms in adjacent polymer chains (Figure 12a). To mitigate this, trifluoroethanol, a hydrogen bond disruptor, was introduced into the self-assembly solution, effectively suppressing aggregation and enabling the formation of uniform fiber-like micelles exceeding 1 μm in length. This strategy was further extended to block comicelles, demonstrating its adaptability for hierarchical self-assembly.

**Figure 12:**
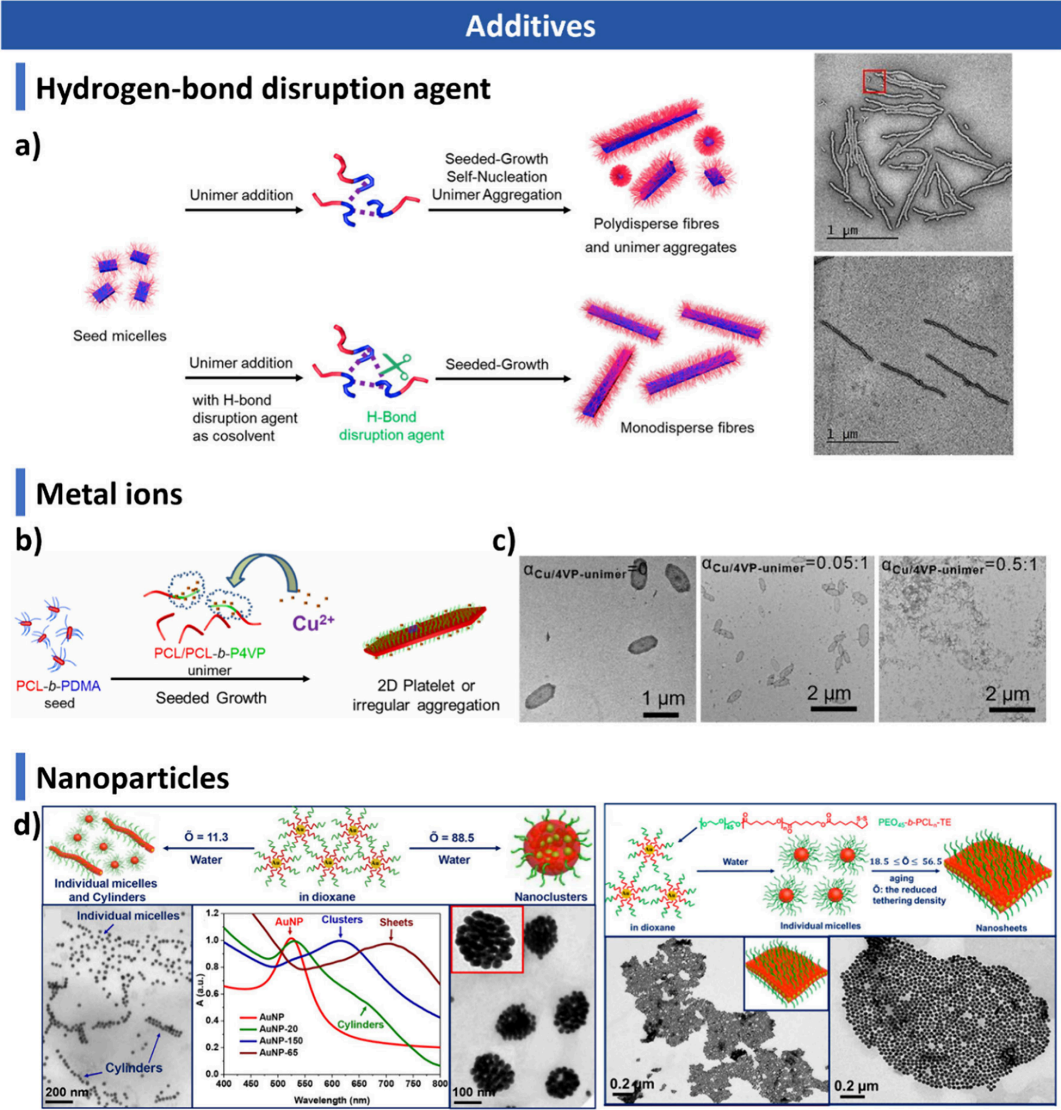
Influence of additives on CDSA. (a) Improved seeded growth of PLLA-based diblock copolymers with hydrogen bond-disrupting agents. Reproduced with permission from ref 143. Copyright 2019 American Chemical Society. (b) Schematic of the “chemical shield” effect from metal ion complexation inhibiting PCL-based seeded growth. (c) TEM images showing Cu^2+^ concentration effects on assembly morphology. Reproduced with permission from ref 361. Copyright 2023 Elsevier Inc. (d) Self-assembly pathway of TE-PCL-*b*-PEO-coated AuNPs, from micelles and cylinders to clustered aggregates and nanosheets. Reproduced with permission from ref 131. Copyright 2018 American Chemical Society.

The Tong group investigated the impact of metal ions (Cu^2+^, Mn^2+^, Zn^2+^, Co^2+^) on seeded growth, finding that metal ion addition created a “chemical shielding” effect, rendering seed micelles inactive.^361^ This effect was particularly pronounced in 4VP-based corona seed micelles, where strong metal–4VP binding inhibited micelle growth. Furthermore, metal ion interactions with P4VP unimers disrupted the self-assembly of PCL/PCL-*b*-P4VP systems, leading to poorly formed structures or irregular aggregates instead of well-defined platelets (Figure 12b,c). These findings highlight the ability of metal ions to selectively influence crystallization dynamics in CDSA.

Mai et al. functionalized gold nanoparticles (AuNPs) with PCL-*b*-PEO diblock copolymers, creating amphiphilic AuNPs that underwent CDSA in aqueous solutions.^131^ The crystallization of PCL blocks, influenced by grafting density and polymer radius of gyration, drove the formation of AuNP nanosheets. The assembly process progressed from individual micelles to small nanosheets and finally to well-defined, large nanosheets (Figure 12d). The structural evolution was reflected in localized surface plasmon resonance absorption shifts, transitioning from nanoclusters to nanocylinders to nanosheets, with a strong near-infrared absorption peak at ∼700 nm. This study illustrates how polymer crystallization can guide the assembly of plasmonic nanostructures with tunable optical properties.

Li and co-workers reported a universal one-pot initiation-growth method using liquid crystalline block copolymers to achieve precise control over hierarchical nanostructures.^43^ By adding small-molecule, macromolecular, or nano-object initiators to P2VP-*b*-PFMA solutions, they triggered nucleation via supramolecular interactions, followed by growth of cylindrical micelles driven by liquid crystalline ordering. This approach enabled the formation of uniform 1D, branched, and star-like nanostructures, with tunable dimensions and morphologies.

In a more recent example, Feng and co-workers developed a one-step/one-pot crystallization-driven coself-assembly strategy to construct π-conjugated-polymer-based three-dimensional nanostructures.^210^ They employed hydrogen bonding between P2VP segments and poly­(styrene sulfonic acid) (PSS) to modulate the nucleation and growth of OPE-*b*-P2VP block copolymers. This enabled the formation of uniform flower-like nanostructures, composed of a core surrounded by radially protruding nanofibers. The approach allowed tuning of fiber number and length through the adjustment of additive content and copolymer concentration.

Additives such as hydrogen bond disruptors, metal ions, and nanoparticles are powerful tools for modulating self-assembly dynamics, crystallization behavior, and structural evolution in CDSA.^2,5^ These strategic modifications help suppress unimer aggregation, regulate nucleation, prevent seed inactivation, and enhance functional integration. The studies discussed highlight how tailored additive incorporation enables controlled morphology, facilitating the formation of fiber-like micelles, platelets, and plasmonic nanosheets.^131,361^ Additionally, the introduction of functional components such as metal ions and nanoparticles paves the way for enhanced optical, catalytic, and electronic properties, expanding the potential applications of CDSA-derived materials.^164^

#### 3.2.6 Other Parameters

Beyond conventional parameters such as block length and ratio, homopolymer content, solvent, temperature, and additives, other factors—including chain-end functionality, polymer dispersity, solution pH, and polymerization rate, and external stimuli—also significantly impact CDSA behavior, stability, and morphology. These parameters influence nucleation, growth, colloidal stability, and self-assembly kinetics, providing additional tools for controlled nanostructure formation.

Pure homopolymers often suffer from poor colloidal stability due to hydrophobic crystalline blocks, which promote aggregation. Surface charge modifications can mitigate this issue.^389^ Manners and co-workers introduced charged end groups to stabilize planar PFS-based structures, enabling the formation of colloidally stable 2D platelet micelles with controlled dimensions.^390^ Using PLLA_24_[PPh_2_Me]I and PLLA_34_[PPh_2_Me]­I, they synthesized diamond-shaped 2D assemblies, where the functional corona [PPh_2_Me]I provided lipophilic charged surfaces, enhancing stability. Furthermore, functionalized surfactant counterions (I, SDS, AOT) effectively suppressed unwanted self-nucleation and unimer aggregation, enabling higher-concentration CDSA with long-term colloidal stability.^389^

Polymer dispersity plays a crucial role in dictating morphology and uniformity in CDSA assemblies.^36^ The Winnik group examined PFS-*b*-PDMAEMA BCPs with similar compositions and number-average DP but varying dispersities under identical CDSA conditions. Low-dispersity BCPs formed rod-like micelles with uniform widths but polydisperse lengths, whereas higher-dispersity samples yielded size-uniform but morphologically diverse structures (Figure 13a). Increased dispersity promoted branching, especially in 1-octanol, leading to 3D structures at the highest dispersities. Mixing low-dispersity BCPs with varying PDMAEMA chain lengths generated 1D cylindrical micelles, branched insect-like structures, and spherical pompoms with densely packed domains (Figure 13b), highlighting dispersity-driven structural diversity.

**Figure 13:**
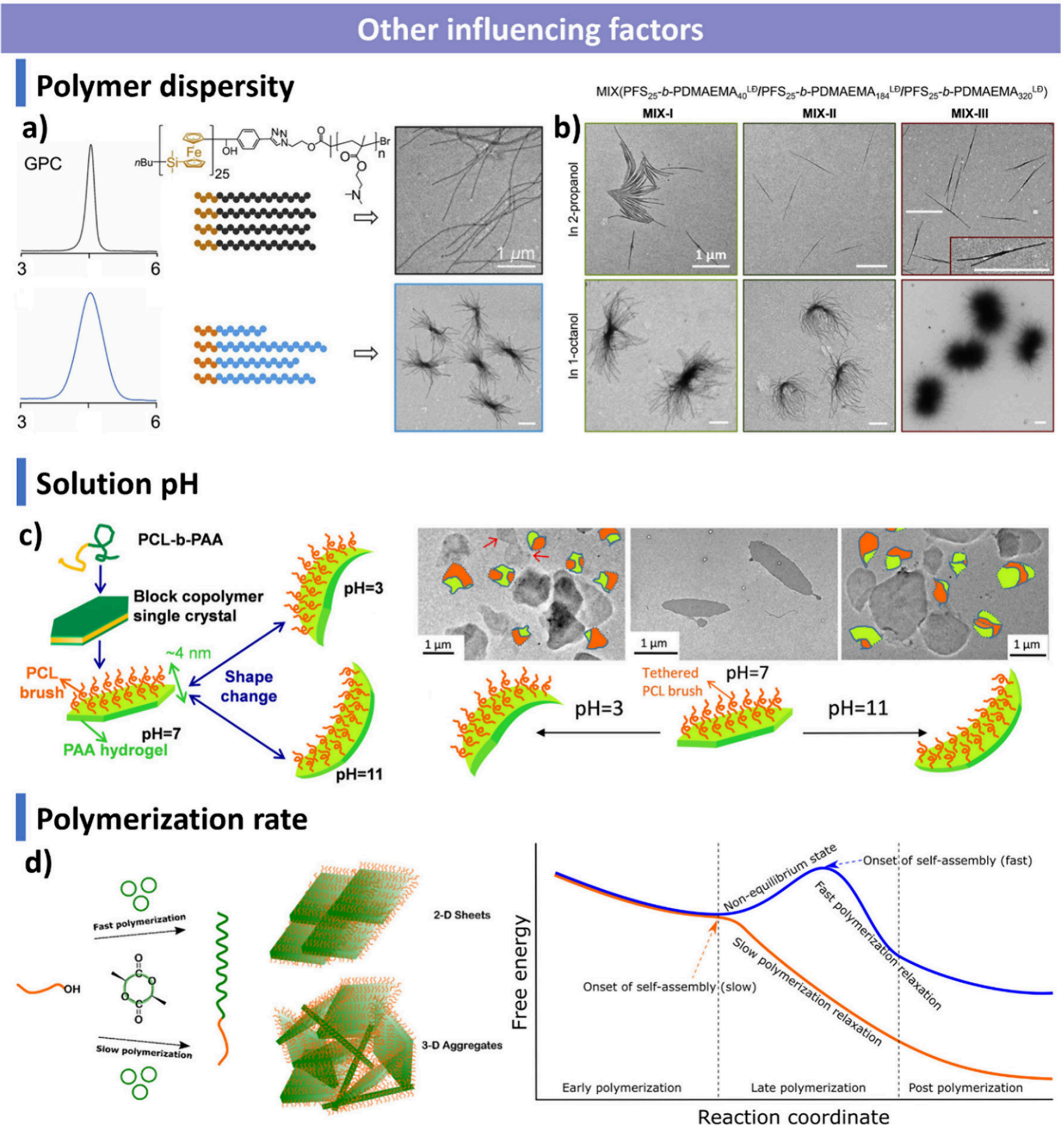
Other influencing factors on CDSA. (a) Effect of polymer dispersity on assembly morphology. (b) TEM images showing uniform structures formed by three block copolymer blends in 2-propanol and 1-octanol (scale bars: 1 μm). Reproduced with permission from ref 36. Copyright 2021 Elsevier Inc. (c) pH-responsive shape changes of Janus PCL-*b*-PAA nanoplates at pH 3, 7, and 11. Reproduced with permission from ref 129. Copyright 2016 American Chemical Society. (d) Effect of polymerization rate on morphology during ROPI-CDSA, with a free energy diagram comparing fast and slow polymerization pathways. Reproduced with permission from ref 145. Copyright 2022 American Chemical Society.

pH-responsive polymers allow controlled morphological transitions in CDSA. Li et al. synthesized PCL-*b*-PAA Janus polymer nanoplates, which undergo reversible shape transformations based on environmental pH.^129^ These platelet-shaped single crystals function as nanoscale “sandwiches,” where PCL lamellae are confined between two PAA layers. At neutral pH (pH 7), the nanoplates maintain a flat morphology. Under acidic conditions (pH 3), they bend, while under alkaline conditions (pH 11), they adopt a bowl-like shape with nanoparticle-decorated surfaces (Figure 13c). These shape transitions are fully reversible, returning to their original flat form after multiple pH cycles (7 → 3 → 7 → 11 → 7), demonstrating robust and tunable pH-dependent actuation.

Polymerization kinetics influence self-assembly pathways in ROPI-CDSA. Patterson and co-workers studied PLLA-*b*-PEG self-assembly, showing that catalyst selection, which controls polymerization rate, dictates final morphology independently of polymer structure or concentration.^145^ Slow polymerization led to increased PLLA chain folding, promoting early stage self-assembly with slower kinetics, resulting in 3D network formation. In contrast, fast polymerization produced less folded PLLA chains, delayed self-assembly initiation, and faster kinetics, yielding 2D structures (Figure 13d). This effect arises from differences in solvophobic block length at the onset of self-assembly, with fast polymerization creating a transient nonequilibrium state, favoring 2D morphologies. This study highlights polymerization rate as a tunable parameter for directing nanostructure formation.

Functional group design along the polymer main chain can selectively modulate CDSA pathways. Gao et al. demonstrated that alternating copolymers with flexible alkenyl or rigid triazole linkers yield distinct assembly outcomes depending on linker chemistry.^391^ Alkenyl-linked copolymers followed a kinetic pathway, forming planar 2D flakes that transformed into crystalline structures upon annealing, while triazole-linked systems underwent direct thermodynamic assembly into highly ordered spindle-like morphologies.

Thermoresponsive living CDSA systems have also been developed. Kempe and co-workers reported that temperature-sensitive poly­(2-oxazoline)-based BCPs exhibit reversible nanorod assembly and disassembly via controlled thermal cycling.^392^ The crystallization temperature range was tuned through block composition, allowing precise thermal control over nucleation and growth kinetics. This enables reconfigurable and recyclable CDSA systems for dynamic applications.

In addition, light can serve as an external stimulus to control CDSA. The Choi group introduced a light-induced living CDSA approach using diarylethene-functionalized conjugated polymers. Upon UV irradiation, trans–cis isomerization enhanced crystallinity, triggering rapid nucleation and nanofiber growth.^367^ Light exposure duration tuned fiber length and uniformity in a one-pot process, showcasing spatiotemporal control of CDSA for optoelectronic nanomaterials. Similarly, Tong, Xie, and co-workers designed a light-responsive CDSA system based on PCL copolymers bearing styrylpyrene groups capable of reversible photoinduced topological transformations.^379^ Light-triggered formation of cyclic or tadpole-like architectures altered chain packing during seeded growth, enabling reversible tuning of 2D platelet micelle morphology and fluorescence properties.

While block composition, homopolymers, solvent, temperature, and additives are primary factors in CDSA, chain-end functionality, dispersity, pH, polymerization rate, main chain design, and external stimuli such as light and heat also play crucial roles in morphology control, stability, and self-assembly pathways. These factors offer additional strategies for tailoring CDSA architectures, enabling controlled formation of responsive, hierarchical, and functional nanostructures. Understanding their effects allows for the design of advanced nanomaterials with enhanced stability, responsiveness, and tunable properties.

### 3.3 Kinetics in CDSA

#### 3.3.1 Observation of CDSA Assemblies’ Kinetics

Despite extensive studies on the effects of core-forming block composition, block ratio, homopolymer content, and solvent conditions on CDSA morphology and dimensions, direct observation of the self-assembly process remains challenging. Understanding how CDSA nanostructures form and evolve is essential for achieving controlled self-assembly. However, capturing the intricate dynamics of this process has been difficult, with only a few experimental approaches capable of providing detailed insights. Advanced techniques such as AFM, TEM, confocal microscopy, and interferometric scattering (iSCAT) microscopy have emerged as powerful tools for real-time visualization of micelle growth, nucleation events, and assembly pathways, allowing direct monitoring of self-assembly evolution.^220,242,393^

In 2018, the Manners group investigated the growth kinetics of living CDSA using PFS-*b*-PDMS block copolymers as a model system, employing *ex situ* TEM analysis.^394^ Several factors—polymer concentration, temperature, solvent quality, and BCP composition—were found to strongly influence micelle elongation rates (Figure 14a). Lower temperatures, poorer solvents for the crystalline PFS core, and longer PFS segments all accelerated self-assembly. Interestingly, the kinetics of living CDSA deviated from classical living covalent polymerization, as the growth rate was neither first-order in unimer concentration nor in seed concentration. Instead, the data fit a stretched exponential function, attributed to the influence of polymer chain conformation and molecular weight polydispersity on unimer addition at micelle termini.

**Figure 14:**
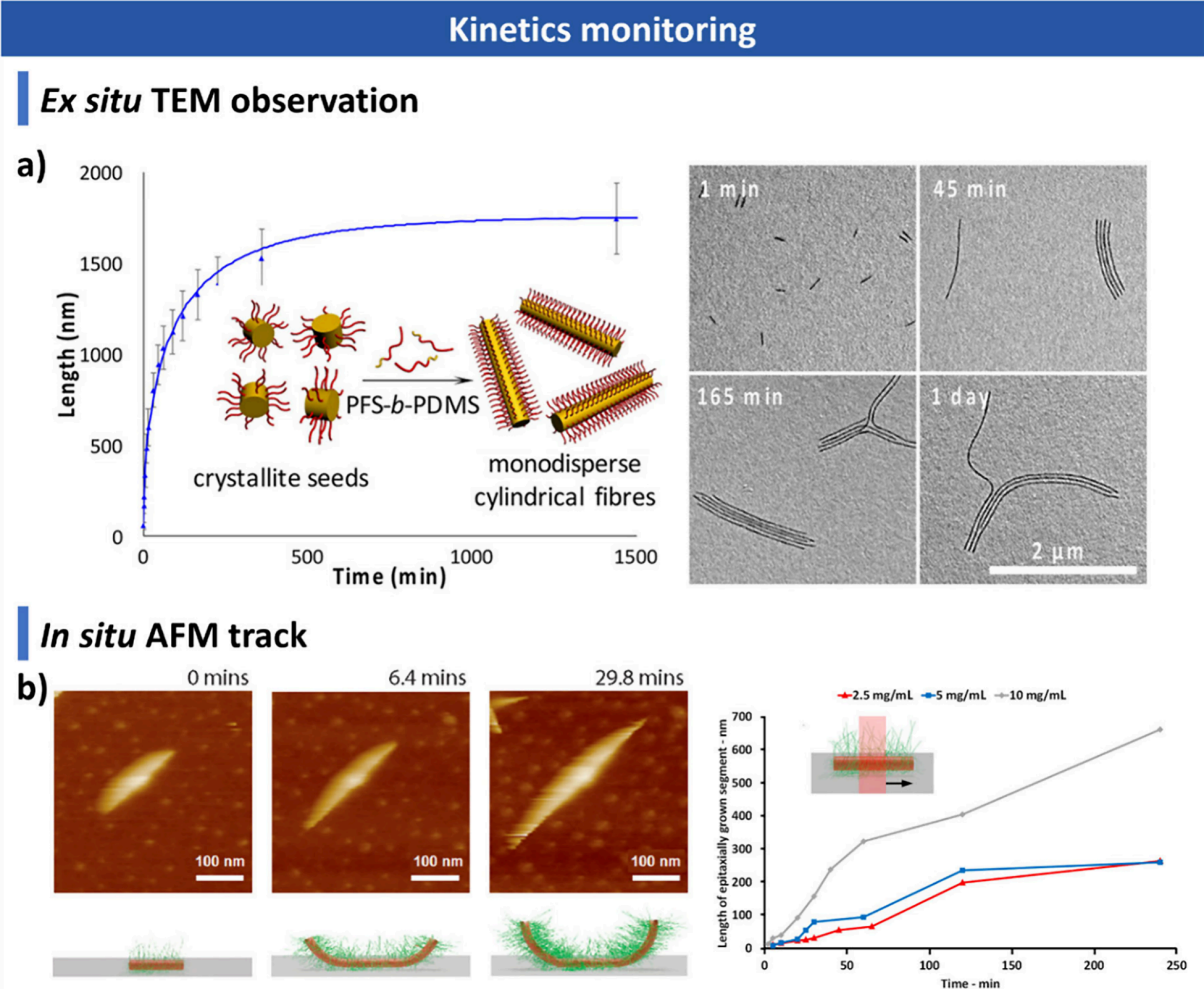
Kinetic monitoring for CDSA. (a) *Ex situ* TEM showing length evolution of PFS_63_-*b*-PDMS_513_ micelles over 2 days after unimer addition, with TEM images at different time points. Reproduced with permission from ref 394. Copyright 2018 American Chemical Society. (b) *In situ* AFM tracking of surface-bound seeded growth, showing AFM images and length evolution of PFS_20_-*b*-P2VP_374_ nanofibers on silicon in iPrOH. Reproduced with permission from ref 395. Copyright 2022 American Chemical Society.

Further extending the study of living CDSA kinetics, Manners and co-workers examined the seeded growth of PFS_20_-*b*-P2VP_374_ fiber-like micelles from surface-bound seeds on silicon substrates.^395^ Using *ex situ* and *in situ* AFM, the study revealed a four-stage interfacial growth process (Figure 14b). Initially, micelle elongation was strongly influenced by the substrate, resulting in rapid growth within a surface-bound regime. As the process continued, the influence of the surface weakened as the newly grown micelle segments detached from the substrate, eventually transitioning into a regime where growth became independent of surface effects. This work highlights key differences between bulk solution and interfacial CDSA, providing a framework for tailoring substrate-mediated nanostructures.

Recently, O’Reilly, Wallace, and co-workers employed iSCAT microscopy to track the real-time growth dynamics of PCL-based fibers and platelets.^384^ This label-free, high-sensitivity technique enabled detailed mapping of reaction parameters affecting growth rate, size, and morphology. In the 1D PCL_73_-*b*-PDMA_204_ system, fiber elongation proceeded faster than in the previously reported a PFS_63_-*b*-PDMS_513_ cylinder system (Figure 15a).^394^ For 2D platelet formation (PCL_45_/PCL_45_-*b*-PDMA_348_), over 210 platelets were recorded during a 25 min time-lapse, allowing analysis of area evolution in real time (Figure 15b). The low deviation in platelet areas indicated convergence toward a stable mean, independent of spatial position. With submillisecond temporal resolution, iSCAT provided unprecedented insights into early stage growth dynamics, micelle trajectories, and size evolution, highlighting its potential for high-resolution kinetic studies of CDSA.

**Figure 15:**
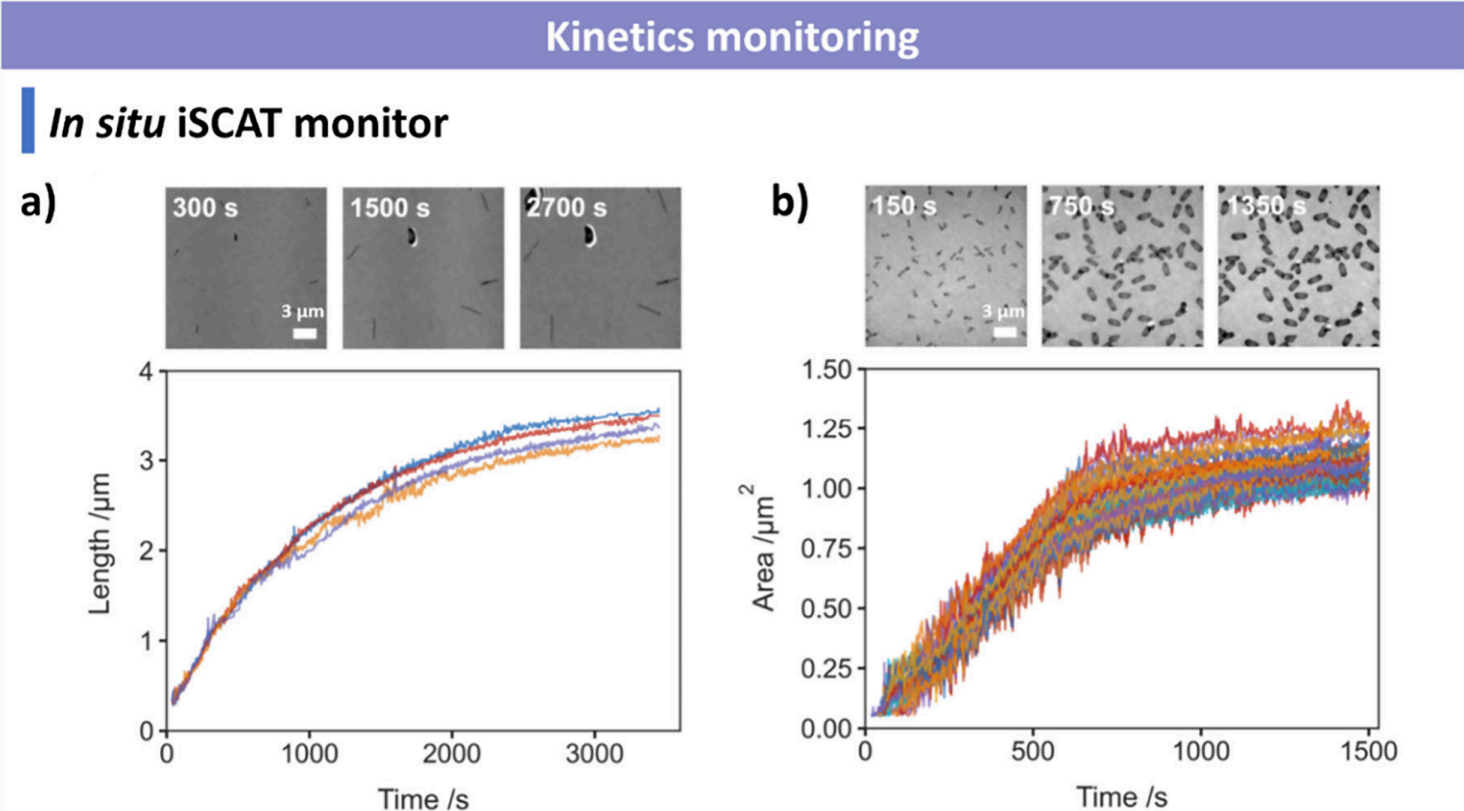
Kinetic monitoring for CDSA. (a) iSCAT images and length evolution of PCL_73_-*b*-PDMA_204_ fiber growth. (b) iSCAT images and size evolution of PCL_45_/PCL_45_-*b*-PDMA_348_ platelet growth (scale bar: 3 μm). Reproduced with permission from ref 384. Copyright 2025 Springer Nature.

Lee and co-workers utilized liquid-phase TEM (LP-TEM) to investigate the dynamic self-assembly pathways of semicrystalline poly­(ethylene oxide)-*b*-poly­(ε-caprolactone) (PEO-*b*-PCL) block copolymers in aqueous condition.^396^ Through systematic variation of block lengths and compositions, they observed the progression of morphological transformations from unimers to spherical micelles, cylindrical micelles, toroidal micelles, and vesicular structures. Beyond structural imaging, LP-TEM enabled quantitative tracking of particle dynamics, uncovering key kinetic parameters such as anomalous diffusion behavior, interparticle interactions, and long-range hydrophobic-driven aggregation processes. The study also highlighted distinct mechanisms for toroidal structure formation, influenced by core crystallinity and interfacial curvature effects.

Kinetic studies of CDSA assemblies using TEM, AFM, and iSCAT microscopy have provided fundamental insights into self-assembly mechanisms, enabling comparisons between solution-phase and substrate-confined growth. The influence of core chemistry on self-assembly rates has also been extensively explored, revealing significant variations in kinetics and structural outcomes. However, several critical aspects of real-time CDSA evolution remain unresolved, including polymer chain folding dynamics, 3D structural development, height and edge/interface characteristics, crystal refractive index, and crystallinity changes during growth. From our perspective, two key directions warrant future exploration: (1) the development of advanced characterization techniques capable of capturing these processes at atomic or near-atomic resolution in semicrystalline materials, including the ability to resolve transient chain conformations, crystalline lattice evolution, and interfacial dynamics. The integration of such techniques with correlative multimodal imaging and spectroscopy is expected to provide unprecedented insights into CDSA mechanisms; and (2) the rational design of CDSA systems optimized for real-time studies, incorporating tailored assembly kinetics, core chemistries, and interfacial properties to enable systematic and mechanistically informative investigations.

#### 3.3.2 Kinetics Application of CDSA Nanostructures

Kinetics play a fundamental role in controlling self-assembly pathways and determining final morphology in CDSA. Regulation of growth rates, solvent conditions, and polymer composition enables the formation of uniform micelles, transitions between linear and branched architectures, and the design of hierarchical structures.^238^ Adjusting unimer concentration, solvent polarity, temperature, and block copolymer composition provides control over assembly pathways and functionality.^397–399^ While kinetic control is critical for 1D cylindrical micelles, it is equally essential in 2D platelet formation, where subtle variations in growth dynamics influence morphology, stability, and surface properties. In 2D systems, the interplay between crystallization growth rates and unimer composition dictates platelet uniformity, corona-chain distribution, and overall dimensions, allowing controlled tuning of functional properties.

The Manners and Winnik groups investigated whether fiber-like micelles exhibit length-dependent growth during living CDSA.^393^ To test this, they conducted seeded growth experiments under identical conditions (concentration, temperature, solvent, and BCP composition) for short (∼100 nm) and long (∼1000 nm) micelles with PFS cores. This experimental design established competition for unimer addition between short and long seeds. Surprisingly, growth rates at the micelle termini were identical, regardless of fiber length or core composition (Figure 16a), which is consistent with the idea originally from Flory that propagation rates in polymerization reactions are independent of the chain length. These findings challenge the notion that low dispersity in living CDSA arises from a gradual decrease in growth rate with increasing fiber length. Instead, the results align with living covalent polymerization models, reinforcing the mechanism of controlled length and low dispersity in CDSA.

**Figure 16:**
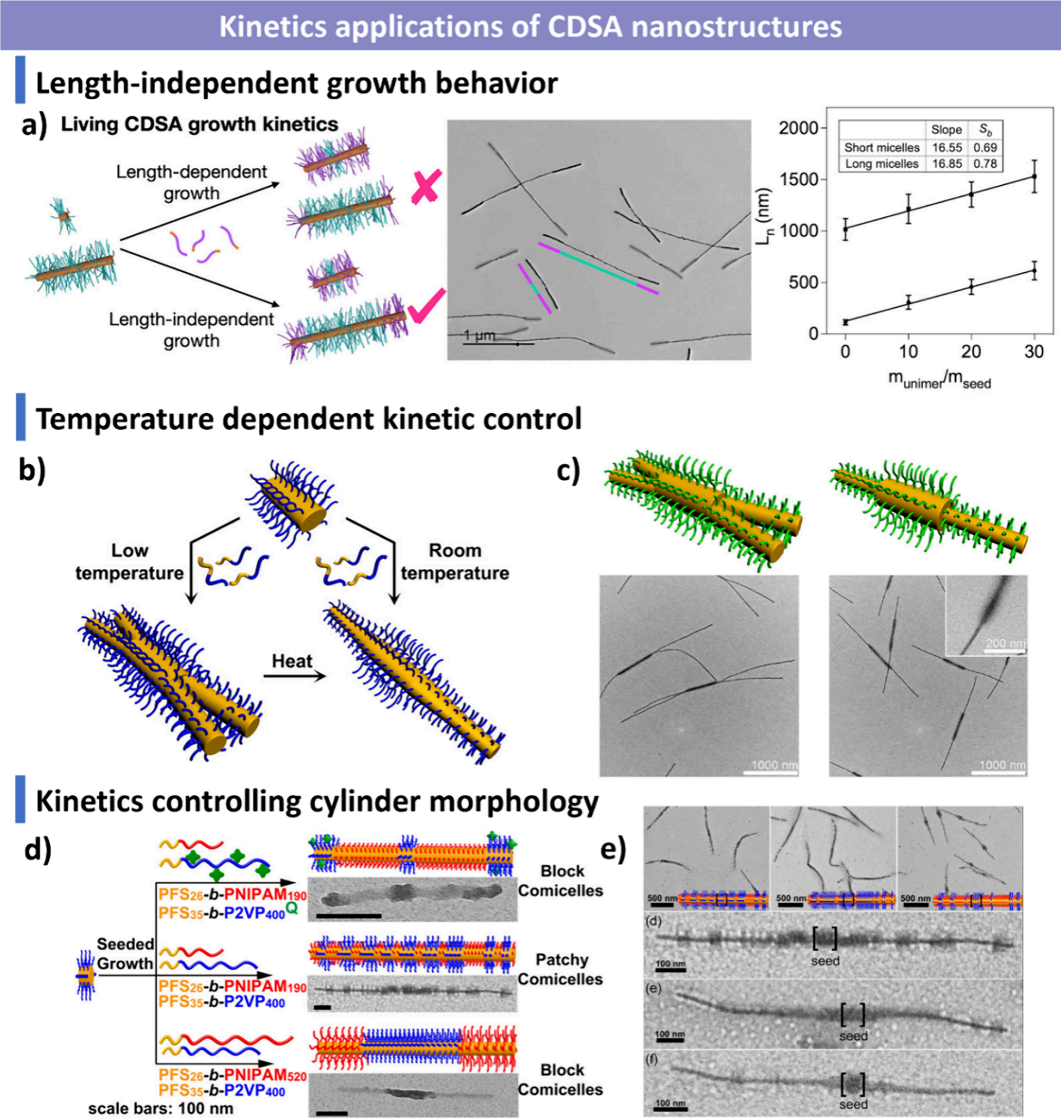
Kinetics application of nanostructures. (a) Length-independent growth of PFS_26_-*b*-P2VP_325_ micelles, showing a linear relationship between micelle length and unimer-to-seed mass ratio. Reproduced with permission from ref 393. Copyright 2021 American Chemical Society. (b) Temperature-controlled transformation of branched micelles into linear micelles. (c) TEM images showing branched micelles of PFDMS_48_-*b*-P2VP_414_ and PFDMS_20_-*b*-P2VP_140_ before annealing, and linear micelles after heating at 80 °C for 2 h (scale bar: 1000 nm). Reproduced with permission from ref 400. Copyright 2015 American Chemical Society. (d) Schematic and (e) TEM images of patchy micelles with phase-separated corona chains formed via competitive seeded growth at PFS_35_-*b*-P2VP_400_ to PFS_26_-*b*-PNIPAM_190_ mass ratios of 1:1, 3:1, and 1:3. Reproduced with permission from ref 78. Copyright 2018 American Chemical Society.

Qiu and co-workers demonstrated that solvent polarity and temperature strongly influence the morphology of micelles formed via CDSA.^400^ In less polar solvents, which act as moderately poor solvents for PFDMS, and at elevated temperatures, linear micelles were predominant. In contrast, when more polar solvents (strongly poor solvents for PFDMS) were used at ambient or subambient temperatures, branched micelles and block comicelles with spatially distinct corona chemistries were obtained (Figure 16b,c). The formation of branched micelles was attributed to rapid crystallization of unimers at micelle termini under nonequilibrium conditions, leading to kinetically trapped structures. Supporting this hypothesis, branched micelles transformed into linear micelles upon heating, indicating temperature-dependent morphology reversibility.

Sequential block comicelle formation through multiple unimer additions is a well-established process, but competitive crystallization kinetics between different BCPs on a common seed remains less explored. Winnik and co-workers investigated competitive seeded growth in PFS-based BCPs, revealing that kinetic differences govern comicelle morphology.^78^ When two BCPs with similar epitaxial growth rates were introduced on the same seed, patchy comicelles with microphase-separated corona chains formed. However, introducing long corona-forming blocks or charged corona chains altered growth rates, leading to large-scale corona separation and block comicelle formation. Importantly, comicelle termini remained active, allowing further hierarchical growth to generate hybrid structures with distinct patchy and single-component blocks (Figure 16d,e). These findings highlight how kinetic control and molecular design dictate complex hierarchical nanostructures through CDSA.

Deng et al. examined the competitive crystallization kinetics of PFS homopolymers (HPs) and PFS-*b*-P2VP BCP blends and their influence on 2D micelle morphology using living CDSA.^378^ Their study emphasized the critical balance between PFS segment fraction (f_PFS_) necessary for 2D platelet formation and P2VP corona density required for colloidal stability. The morphology and stability of platelets were determined by the BCP/HP mole ratio and the crystallization growth rate ratio, both of which were tunable by adjusting solvent quality and polymer molecular weight. The study found that platelet morphology depended on the relative mole ratio of BCP to HP ((*c*_B_/*c*_H_)_t_) and the crystallization growth rate ratio ((*G*_B_/*G*_H_)_t_). When (*c*_B_/*c*_H_)_t_ aligned with (*G*_B_/*G*_H_)_t_, uniform platelets with a homogeneous P2VP corona distribution were obtained. However, deviations from this balance led to nonuniform corona distributions, affecting platelet morphology and stability. When (*c*_B_/*c*_H_)_t_ < (*G*_B_/*G*_H_)_t_, P2VP density decreased at platelet termini, promoting end-to-end aggregation into chain-like superstructures. When (*c*_B_/*c*_H_)_t_ > (*G*_B_/*G*_H_)_t_, P2VP density increased at edges and termini, resulting in fusiform micelles.

Controlling kinetics in CDSA provides a powerful approach for directing nanostructure complexity and functionality. The studies discussed demonstrate how fine-tuning kinetic factors—including solvent polarity, temperature, and unimer addition rates—modulates 1D cylindrical micelle growth, morphology transitions, and hierarchical self-assembly. Investigations of 2D platelet formation reveal how kinetic control dictates morphology, stability, and hierarchical organization. By optimizing parameters such as unimer concentration, growth rate ratios, and solvent conditions, controlled regulation of crystallization kinetics and corona-chain distribution enables the design of uniform, functional nanostructures.

## 4 Characterization Techniques in the Investigation of CDSA Mechanisms

Two major challenges are faced in this area of research: the reproducible, scalable and controlled synthesis of such assemblies and the reliable, accurate and in-depth analysis of these materials. This part will focus on this second aspect and provide a guide for the characterization and analysis of soft materials in solution using scattering, microscopic and spectroscopic techniques.^401–405^

### 4.1 Scattering Techniques

Scattering techniques are nondestructive methods that provide realistic insights into nanoparticles in their solution state. These techniques rely on the interaction of light (or other radiation) with matter, resulting in changes in direction, intensity, or polarization of the scattered beam. Among these techniques, common methods including dynamic light scattering (DLS), static light scattering (SLS), small-angle X-ray scattering (SAXS), small-angle neutron scattering (SANS), and wide-angle X-ray scattering (WAXS), differ in their light sources, scattering angles, detectable length scales, and the information they extract. Below, we discuss how these techniques are applied to analyze CDSA nanoparticles.

#### 4.1.1 Dynamic Light Scattering (DLS)

DLS is a widely used and convenient technique for determining the hydrodynamic size of nanoparticles based on their Brownian motion in solution.^406^ The hydrodynamic radius (*R*_h_) is calculated using the Stokes–Einstein eq (eq 1):

$$R_{h}=\frac{k_{B}T}{6\pi \eta D}$$ 1

Where η is the viscosity of the solvent, *k*_B_ is Boltzmann’s constant, *T* is the absolute temperature, and *D* is the diffusion coefficient.^406^ While DLS provides valuable information about particle size, it cannot reveal morphological details, as the calculated *R*_h_ represents the theoretical radius of an ideal hard sphere with an equivalent diffusion coefficient.^402^ In the case of CDSA, where the resulting nanostructures are typically anisotropic, the true diffusion behavior deviates from spherical models. Therefore, the measured diffusion coefficient is better described as an apparent diffusion coefficient (*D*_app_). Although *D*_app_ does not provide direct morphological detail, monitoring *D*_app_ remains useful for assessing relative size variations and stability of nonspherical assemblies.^407^

DLS is widely used as a rapid and effective tool for investigating CDSA behavior under varying conditions, including temperature, solvent composition, polymer structure, additives, pH, and aging time.^175,203,221,307,324,326,358,398,407–409^ For example, the Choi group extensively studied CDSA using conjugated polymers, whose rigid structures are highly sensitive to solubility and significantly influence assembly behavior. They employed DLS to measure hydrodynamic diameter (*D*_h_, where *D*_h_ = 2*R*_h_) as an indicator for polymer structural optimization.^175,218–221,326,367,410^ In their work on P34DHT-*b*-PT polymers, they used an *in situ* assembly strategy to simultaneously conduct polymerization and self-assembly (Figure 17a,b).^326^ The *D*_h_ values increased from 38 to 141 nm as the PT block length grew. Additionally, they used DLS to monitor living seeded growth, observing an increase in *D*_h_ with the addition of more unimer to the seed solution (Figure 17c,d).^221^

**Figure 17:**
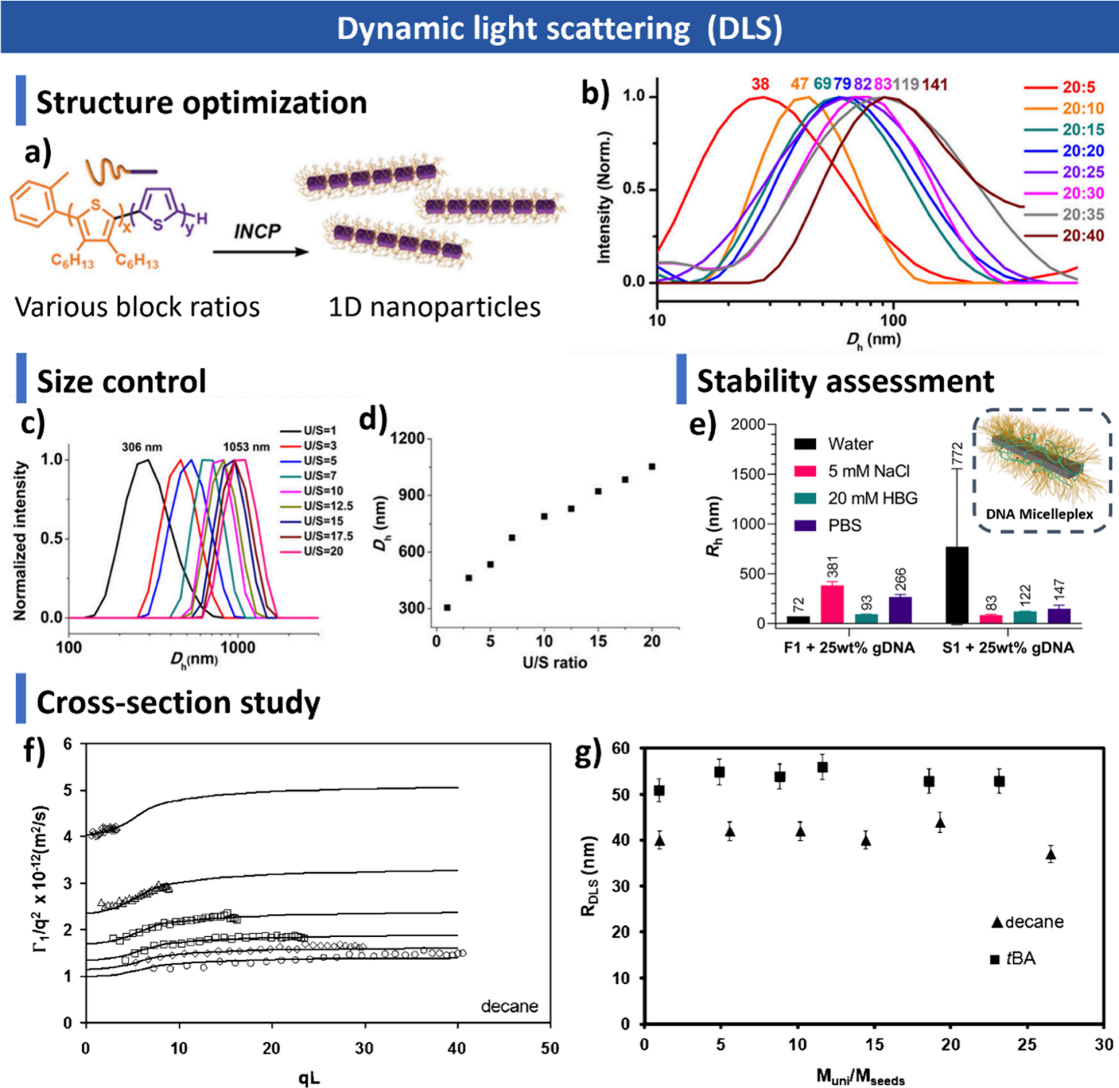
Application of DLS in characterization of CDSA nanoparticles. (a) *In situ* nanoparticlization forming 1D nanoparticles. (b) *D*_h_ of nanoparticles formed from P34DHT-*b*-PT with different block ratios. Reproduced with permission from ref 326. Copyright 2022 American Chemical Society. (c) DLS results and (d) *D*_h_ values of CDSA nanoparticles with increasing unimer content. Reproduced with permission from ref 221. 2023 American Chemical Society. (e) *R*_h_ values in different media. Reproduced with permission from ref 303. Copyright 2023 Royal Society of Chemistry. (f) Multiangle DLS results for cylinders of various lengths. (g) *R*_h_ of cylinders as a function of length. Reproduced with permission from ref 411. Copyright 2012 American Chemical Society.

The Manners group also utilized DLS to assess the stability and size of micelleplexes formed by CDSA nanoparticles and DNA.^303^ They prepared size-defined nanofibers and nanospheres using poly­(fluorenetrimethylenecarbonate)-*b*-poly­(2-(dimethylamino)­ethyl methacrylate) (PFTMC-*b*-PDMAEMA) and complexed them with DNA in various media (Figure 17e). By comparing the sizes of the complexes with the original nanoparticles and DNA, they determined whether DNA was surface-bound or if aggregation occurred. This approach identified 20 mM HEPES buffer with 5 wt % glucose (pH 7.4, HBG) as the optimal medium for CDSA nanoparticle-DNA complexation. These examples highlight the critical and versatile role of DLS in characterizing CDSA nanostructures.

While conventional DLS performed at a single scattering angle is commonly used to estimate the hydrodynamic diameter of colloidal systems, it provides only an averaged size and cannot resolve the distinct dimensions of anisotropic morphologies such as rods or platelets. Multiangle DLS provides a more comprehensive approach by enabling application of shape-dependent theoretical models, allowing extraction of structural parameters such as the hydrodynamic cross-section.^316,411^ For example, Guerin et al. performed multiangle DLS analysis on thick, rigid rod-like micelles formed by PFS_50_-*b*-PI_1000_ (Figure 17f,g). The angle-dependent decay rates revealed differences in hydrodynamic swelling of the micelle coronas, demonstrating that PI chains were more extended in t-butyl acetate than in decane, attributable to improved solvent quality in the former.^411^

#### 4.1.2 Static Light Scattering (SLS)

Unlike DLS, which characterizes particles based on Brownian motion-induced scattering relaxation, SLS measures the time-averaged scattering intensity over durations much longer than the relaxation time, emphasizing the effects of particle size and morphology.^402^ Although SLS measurements are more complex and time-consuming—requiring data collection at multiple concentrations and angles for a single sample—they provide comprehensive structural information, including molecular weight, aggregation number, radius of gyration, and particle shape.^402,412^ These parameters are determined using the Zimm eq (eq 2).

$$\frac{Kc}{R_{\theta }}=\frac{q^{2}R_{g}^{2}}{3M_{w}}+\frac{1}{M_{w}}+2A_{2}c$$ 2

where *K* is the optical constant, *c* is the concentration, *R*_θ_ is the Rayleigh ratio, *q* is the scattering vector, *R*_g_ is the radius of gyration, *M*_w_ is the molecular weight of nanoparticles, *A*_2_ is the second virial coefficient.

To better characterize rigid rod-like CDSA nanoparticles, the Winnik group presented the SLS results of cylinders by Holtzer-Casassa (HC) plots according to the following eq (eq 3).^316,413–415^

$$f\left(q\right)=\frac{qR_{\theta }}{\pi M_{w}Kc}=\frac{P\left(q\right)q}{\pi }L_{w}N_{\mathrm{agg}/L}$$ 3

where *P*(*q*) is the form factor, *N*_agg/L_ is the number of polymer chains per nanometer of micelle length, and *L*_w_ is the weight-averaged length of cylinders. Different form factor models are applied in SLS analysis depending on the micelle geometry. For thin, rigid rods—where the cross-sectional radius is negligible compared to the length—the form factor simplifies, allowing extraction of only *N*_agg/L_.^30,407,416^ In contrast, for thicker rods, the analysis must account for the cross-sectional dimensions, and additional structural parameters, such as radius, can be obtained.^316,411,417^

The practicality and accuracy of this strategy were demonstrated by the size consistency of PFS-based cylinders characterized by SLS and TEM (Figure 18a,b).^316^ Furthermore, when fitted using the thin rod model, SLS provided detailed structural information, including both *N*_agg/L_ and the cross-sectional radius, under appropriate geometric assumptions. The same group also utilized SLS to monitor the temperature-responsive behavior of cylinder coronas, serving as a model to investigate the collapse behavior of surface-attached polymers (Figure 18c,d).^413^ The results showed that the poly­(oligoethylene glycol methacrylate) (POEGMA) corona exhibited a broad and continuous collapse transition, contrasting with the sharp LCST transition observed in dissolved homopolymers.

**Figure 18:**
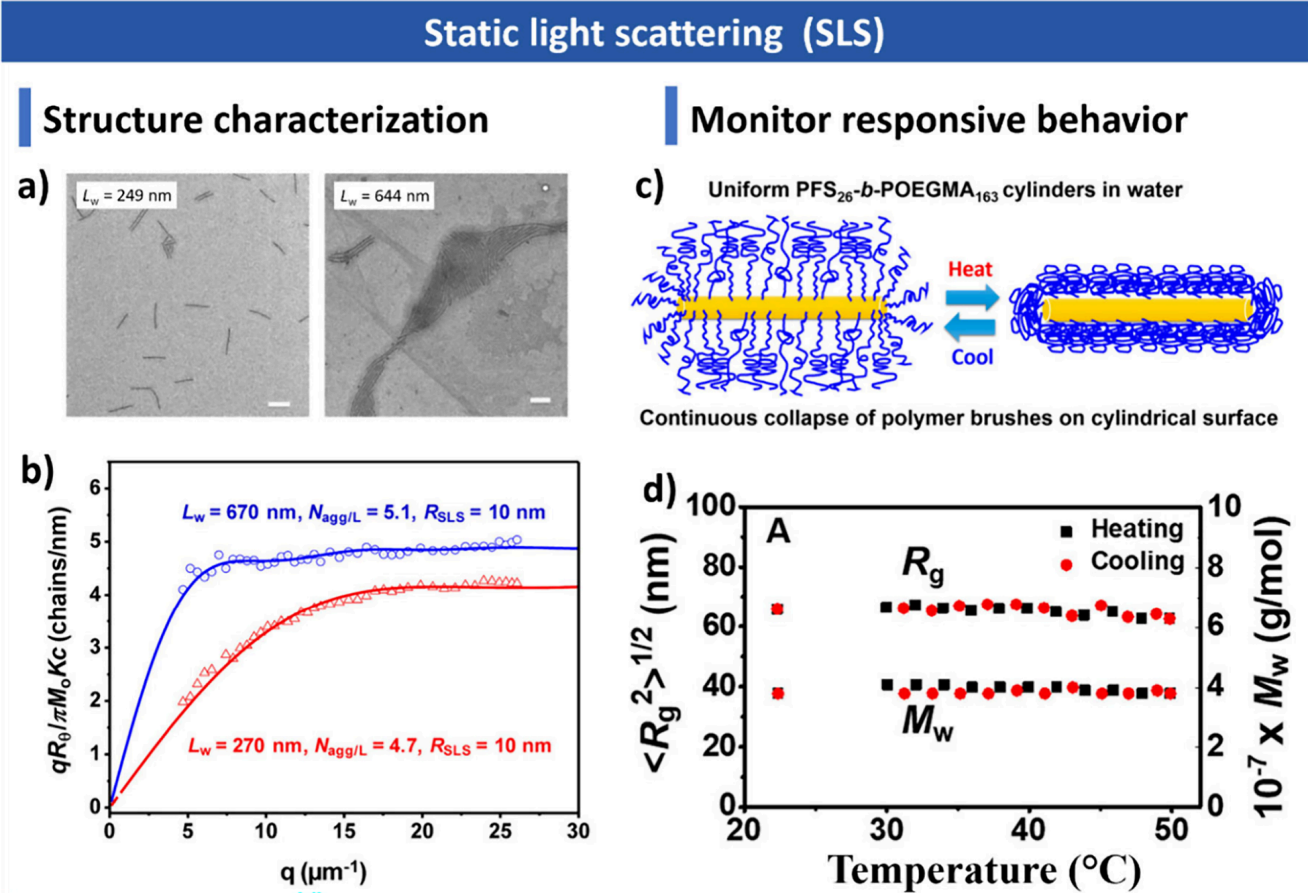
Application of SLS in characterization of CDSA nanoparticles. (a) TEM images of cylinders with defined lengths (scale bar = 200 nm) and (b) corresponding SLS results. Reproduced with permission from ref 316. Copyright 2019 American Chemical Society. (c) Schematic of temperature-responsive behavior of cylinders and (d) changes in *R*_g_ and *M*_w_ with temperature. Reproduced with permission from ref 413. Copyright 2018 American Chemical Society.

#### 4.1.3 Small-Angle X-ray Scattering (SAXS)

SAXS is a more sophisticated and advanced technique compared to DLS and SLS, utilizing short-wavelength X-rays to probe structural features at nanometer scales (1–100 nm).^418^ By measuring scattering at small angles (2θ < 5°), SAXS provides detailed characterization of size, shape, internal chain arrangements, and interchain spacing in complex materials.^402,418^ For CDSA nanoparticles, SAXS has been instrumental in elucidating nanoscale organization and morphology, offering deeper insights beyond conventional light scattering techniques.^172^

Many studies have focused on elucidating the internal structure of CDSA nanoparticles.^419,420^ In addition to conventional size parameters such as *R*_h_, *R*_g_, and *R*_c_ (core radius),^394,419,421–423^ which can also be determined by DLS and SLS, SAXS provides insights into molecular packing.^148,236,266,324,409^ Tong and co-workers developed a one-pot strategy to prepare poly­(behenyl acrylate)-*b*-poly­(N,N-dimethylacrylamide) (PA22C-*b*-PDMA) platelets and utilized SAXS to investigate chain arrangement and platelet formation kinetics.^324^ SAXS analysis (Figure 19a–c) suggested that PA22C-*b*-PDMA forms rod-like units within the core, with a diameter of 5.87 nm and a *d*-spacing of 5.08 nm. Furthermore, the progressively increasing SAXS signal with longer aging times supports the proposed mechanism in which spherical nanoparticles initially form and subsequently fuse stepwise into platelets.

**Figure 19:**
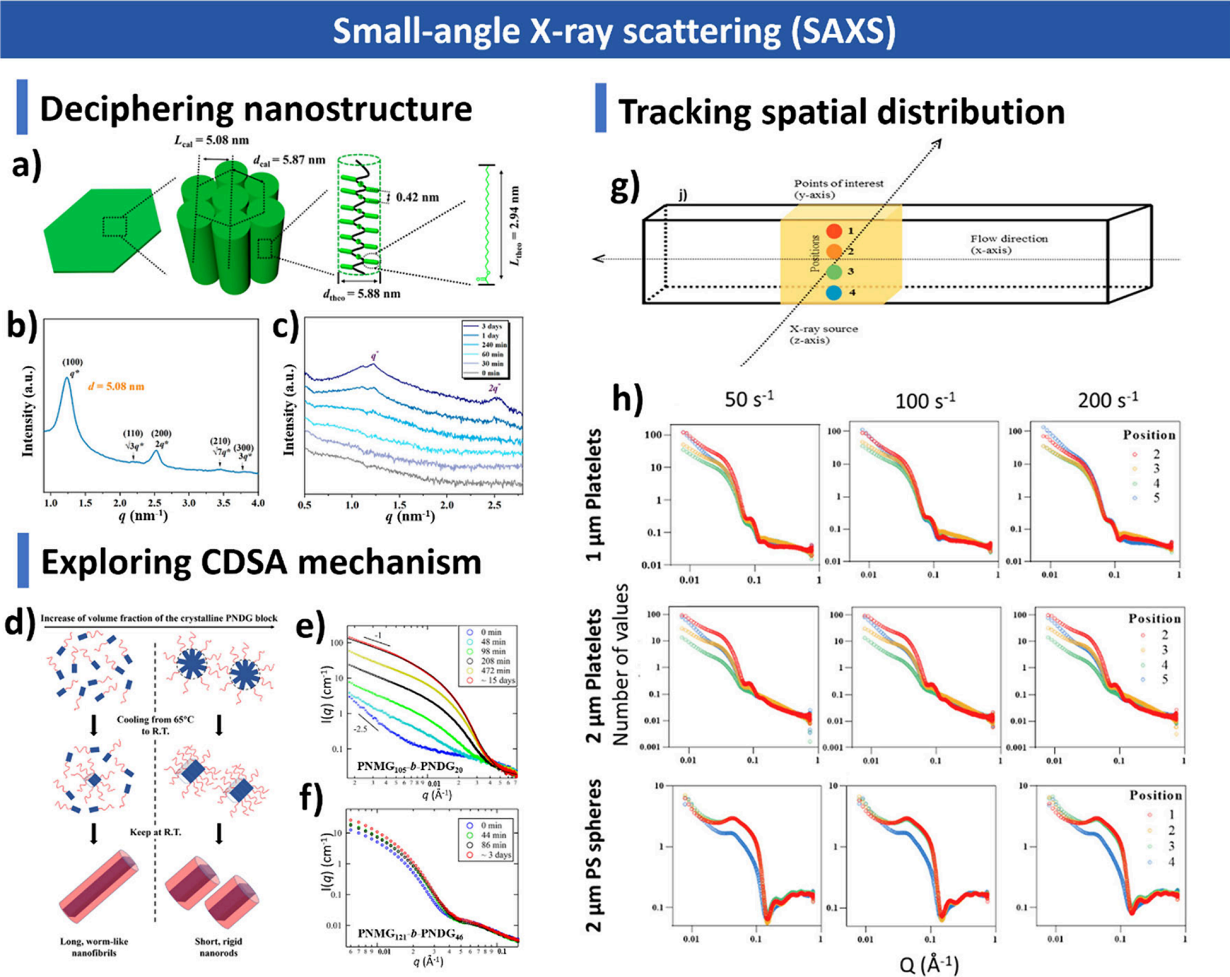
Application of SAXS in characterization of CDSA nanoparticles. (a) Schematic illustration of platelet structure analyzed by SAXS. (b) SAXS profile of solid platelets. (c) Time-resolved SAXS profiles during micelle aging. Reproduced with permission from ref 324. Copyright 2023 American Chemical Society. (d) Proposed self-assembly pathways for nanofibrils and nanorods with varying PNDG fractions. SAXS profiles at different times for (e) PNMG_105_-*b*-PNDG_20_ and (f) PNMG_121_-*b*-PNDG_46_ solutions. Reproduced with permission from ref 424. Copyright 2019 American Chemical Society. (g) Schematic of margination tracking using SAXS in a microfluidic device. (h) SAXS curves collected at different positions in the microfluidic channel. Reproduced with permission from ref 425. Copyright 2023 American Chemical Society.

A more detailed SAXS study on the CDSA mechanism was conducted by Jiang et al., who investigated polypeptoid block copolymers with varying core lengths.^424^ Their findings revealed that polymers with short crystalline blocks preferentially formed long nanofibrils, whereas those with long crystalline blocks formed short nanorods. Time-dependent SAXS profiles showed a significant increase in scattering intensity at low *q* and changes in signal shape for short crystalline block polymers, indicating initial seed formation upon cooling, followed by nanofibril growth (Figure 19d–f). In contrast, polymers with long crystalline blocks formed nanorods via confined crystallization within micelle precursors present before cooling, as evidenced by minimal changes in SAXS signal shape and intensity over time.

SAXS can be utilized for real-time dynamic monitoring.^425,426^ For instance, the Stenzel group employed SAXS to investigate nanoparticle margination—defined as their lateral migration toward vessel walls—by tracking scattering signals across different positions in a flow channel.^425^ This approach provided insights into the local nanoparticle concentration. In this study, the margination behavior of PLLA platelet samples (1-μm and 2-μm in length) was compared with 2-μm PS spherical particles under varying shear rates. SAXS analysis revealed that margination efficiency increased with higher shear rates, and anisotropic platelets demonstrated significantly enhanced margination compared to spherical particles (Figure 19g,h). These results demonstrate the versatility of SAXS as a powerful analytical technique *in situ* characterization of complex dynamic systems.

#### 4.1.4 Wide-Angle X-ray Scattering (WAXS)

WAXS is another X-ray scattering technique based on principles similar to those of SAXS.^418^ However, WAXS operates at larger scattering angles (2θ > 5°), enabling the measurement of atomic-scale structural features (<1 nm), such as crystalline lattices and molecular packing.^418,427^ Consequently, WAXS is particularly useful for characterizing the crystalline core of CDSA nanoparticles.^357,398,420,422,428–432^

CDSA is a crystallization process, making WAXS a suitable technique for detecting crystallinity and monitoring the CDSA process.^372,398,431^ For instance, Lecommandoux and co-workers synthesized poly­(2-isopropyl-2-oxazoline)-*block*-poly­(2-methyl-2-oxazoline), which exhibited lower critical solution temperature (LCST) behavior at 57 °C.^431^ Upon annealing the polymer solution at 65 °C, crystallization occurred, driving the transformation of amorphous spherical particles into micron-sized fiber-like structures (Figure 20a). This CDSA process was monitored using WAXS, which revealed enhanced scattering signals with increasing annealing time, confirming the growth of crystalline domains (Figure 20b).

**Figure 20:**
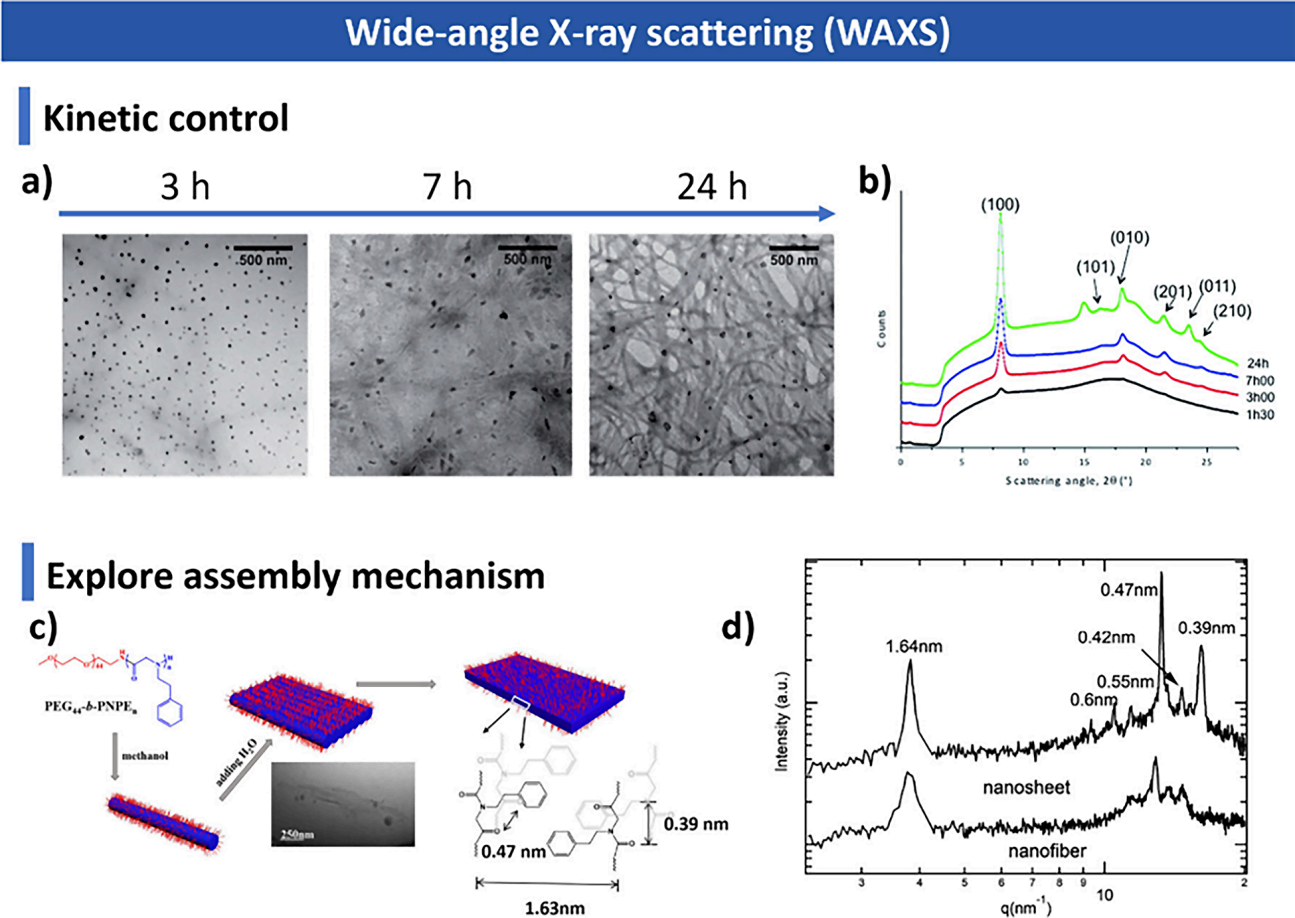
Application of WAXS in characterization of CDSA nanoparticles. (a) TEM images and (b) WAXS patterns of cylinders at different time points (scale bar = 500 nm). Reproduced with permission from ref 431. Copyright 2015 Royal Society of Chemistry. (c) Morphological transition from nanofibers to nanosheets and (d) corresponding WAXS profiles showing structural differences. Reproduced with permission from ref 254. Copyright 2019 American Chemical Society.

WAXS is also valuable for elucidating the mechanisms of morphological changes, as different morphologies often exhibit distinct crystal structures.^254,420,422,428^ For example, Wei et al. investigated morphological change using amphiphilic poly­(ethylene glycol)-*b*-poly­(*N*-(2-phenylethyl)­glycine) (PEG-*b*-PNPE) polymers (Figure 20c).^254^ They observed that self-assembled nanofibers and nanosheets displayed different crystal structures, reflected in their unique WAXS scattering profiles (Figure 20d). By adding water to a methanol solution of nanofibers, the transformation of nanofibers into nanosheets was achieved, a process that was visually confirmed using microscopy. This demonstrates the utility of WAXS in correlating structural and morphological changes during CDSA.

#### 4.1.5 Small-Angle Neutron Scattering (SANS)

In addition to the previously mentioned techniques, SANS is another scattering method that operates similarly to SAXS but uses neutrons instead of X-rays as the beam source.^402^ Unlike X-rays, which interact with electron clouds, neutrons interact with atomic nuclei, providing unique contrast for hydrogen-rich polymer nanoparticles.^433^ However, due to the limited availability of neutron sources, only a few studies have utilized SANS to characterize CDSA nanoparticles.^424,434–436^ These studies have demonstrated the advantages of SANS in probing internal chain organization and solvent–polymer interactions, which are difficult to resolve using SAXS alone. As neutron facilities continue to advance, SANS could become a more accessible and powerful tool for probing the nanoscale morphology and hierarchical ordering of CDSA nanostructures.

### 4.2 Microscopy

Microscopy is essential for characterizing CDSA, providing direct visualization of nanostructures and insights into morphology, crystallinity, and hierarchical organization.^4,54,403^ Transmission electron microscopy (TEM) enables atomic-scale imaging of crystallization pathways and defect formation,^405^ while atomic force microscopy (AFM) offers quantitative height and adhesion measurements for three-dimensional characterization.^403^ Fluorescence techniques, including confocal laser scanning microscopy (CLSM)^437^ and stimulated emission depletion (STED) microscopy,^438^ enhance spatial resolution and enable real-time visualization of polymer assemblies. Emerging methods such as interferometric scattering (iSCAT) microscopy allow high-speed, label-free tracking of self-assembly kinetics.^439–441^ This section highlights the role of these techniques in elucidating CDSA structures and guiding the design of functional materials.

#### 4.2.1 Transmission Electron Microscopy (TEM)

In CDSA, understanding the structural and morphological features of assemblies is essential for designing systems with tailored properties and functionalities. TEM, with its ability to visualize nanostructures at atomic resolution, is an indispensable tool for unraveling the nanoscale organization and crystallinity of these assemblies.^405,442^ TEM provides detailed insights into assembly mechanisms, growth pathways, and crystalline domains, enabling a deeper understanding of how nanoscale morphology influences macroscopic properties.^35,443,444^

Over the years, TEM has revealed an impressive range of morphologies in CDSA assemblies, highlighting the diversity and tunability of these systems. Structures such as cylindrical micelles, crystalline platelets, interconnected networks, and hollow platelets have been directly imaged. Furthermore, TEM has captured complex hierarchical architectures, including arrow-like, branched, windmill-shaped, and dendritic micelles (Figure 21a). The atomic-level resolution of TEM enables detailed optimization of assembly processes and refinement of material designs, making it an essential tool for studying CDSA.

**Figure 21:**
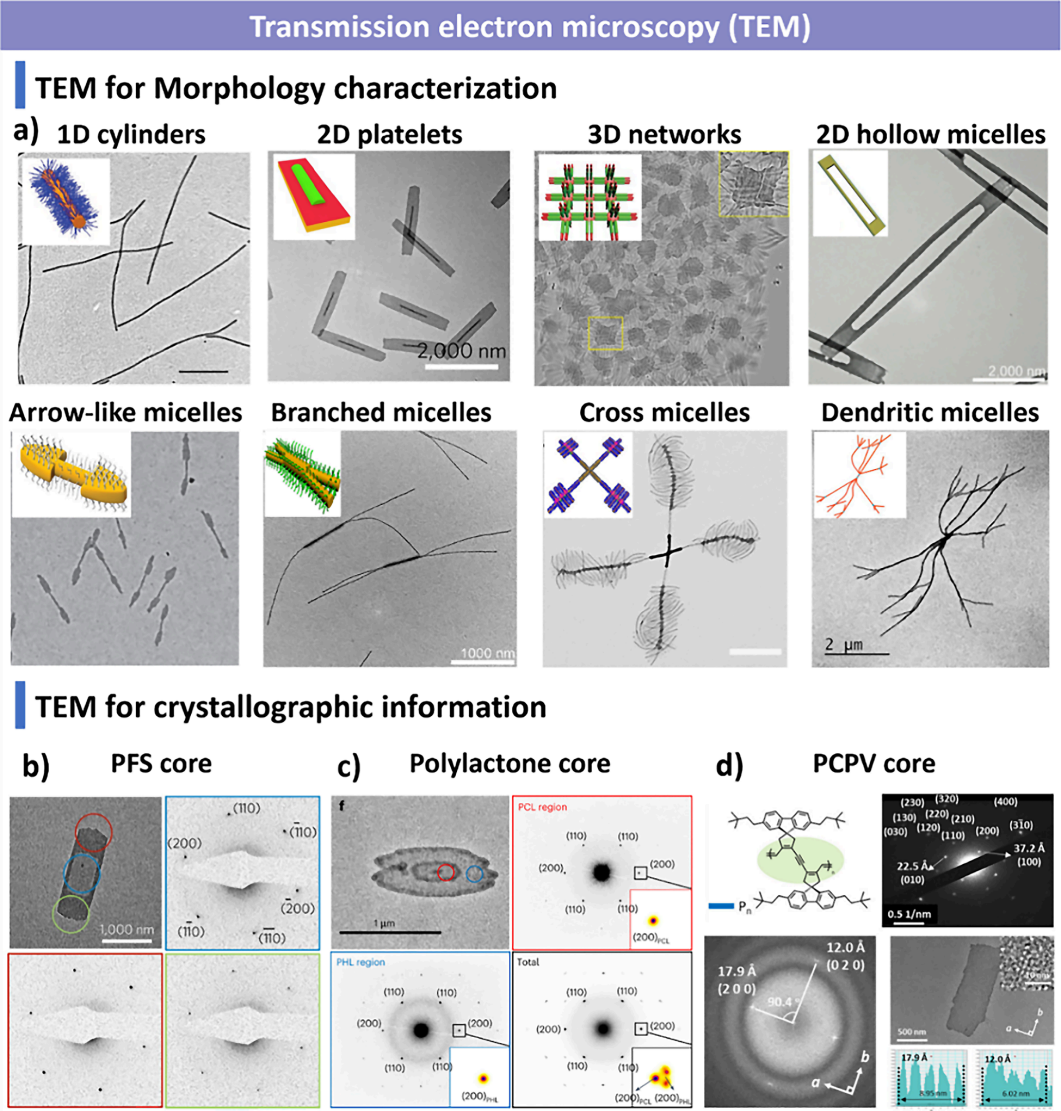
TEM characterization of CDSA assemblies. (a) TEM images showing a variety of morphologies formed via CDSA. Reproduced with permission from refs 30, 88, 314, 331, 343, 344, 390, 400 . Copyright 2007 American Association for the Advancement of Science, 2014 Springer Nature, 2015 Springer Nature, 2021 American Chemical Society, 2015 American Association for the Advancement of Science, 2016 American Association for the Advancement of Science, 2017 Springer Nature, and 2015 American Chemical Society. (b) TEM SAED pattern providing crystallographic information for PFS cores. Reproduced with permission from ref 390. Copyright 2017 Springer Nature. (c) TEM SAED pattern of polylactone cores. Reproduced with permission from ref 38. Copyright 2023 Springer Nature. (d) TEM SAED pattern of PCPV cores. Reproduced with permission from ref 221. Copyright 2023 American Chemical Society.

TEM in selected area electron diffraction (SAED) mode is a powerful tool for analyzing the crystalline structures of nanomaterials, enabling the differentiation of crystallinity and lattice parameters in materials with diverse core structures. SAED analysis not only reveals crystallographic information but also provides insights into the relationship between crystalline planes and the morphology of assemblies.

In the PFS system, He et al. employed SAED to investigate the growth dynamics of various crystalline planes ((010), (100), and (110)) and their influence on the resulting morphologies, such as hexagonal and rectangular assemblies (Figure 21b).^390^ This analysis highlighted the role of anisotropic crystal growth in determining the shape and dimensions of PFS-based nanostructures.

For polylactone systems, the O’Reilly and Dove groups utilized polymers with distinct polylactone cores for epitaxial growth.^38^ SAED patterns were used to examine the crystal structures within spatially and compositionally distinct domains of platelets, particularly for cases involving significant lattice-spacing differences between components. For instance, in block comicelles comprising PCL and poly­(ζ-heptalactone) (PHL), SAED patterns revealed clear distinctions between the PCL and PHL domains, including separated diffraction spots for their respective (200) planes (Figure 21c). This analysis confirmed the segregation of the two phases and provided insights into the structural organization within the platelet assemblies.

In the conjugated polymer PCPV system, Choi and co-workers used SAED to reveal over ten orthogonal diffraction spots, indicating a rectangular crystal unit cell with a and b axes.^221^ The *d*-spacings of 37.2 Å and 22.5 Å corresponded to the a and b axes, respectively. Fast Fourier transform (FFT) analysis of TEM images showed shorter-range periodicities with *d*-spacings of 17.9 Å and 12.0 Å. The axis ((100) direction) aligned with the width-growth direction, while the *b* axis ((010) direction) matched the length-growth direction, providing insights into the structural orientation of the 2D nanorectangles (Figure 21d). These examples underscore the utility of SAED in characterizing crystalline domains and their orientation within CDSA-derived assemblies. The integration of SAED with TEM provides critical insights into the crystallographic parameters, enabling detailed understanding and rational design of nanostructures with tailored morphologies and functional properties.

However, TEM and SAED measurements can induce electron beam damage, particularly in soft matter-based nanostructures. Recently, low-dose techniques for crystal structure determination have been developed to minimize radiation damage in highly beam-sensitive samples, among which serial electron diffraction (SerialED) represents a promising approach.^445^ The Winnik group have employed SerialED to investigate the crystalline structure of PFS-based CDSA assemblies.^349,350^ SerialED was used to confirm that the primary and secondary crystals within complex three-dimensional spherulitic micelles exhibit consistent monoclinic symmetry and lattice parameters, providing critical structural insights into their hierarchical morphological evolution (Figure 22a,b).

**Figure 22:**
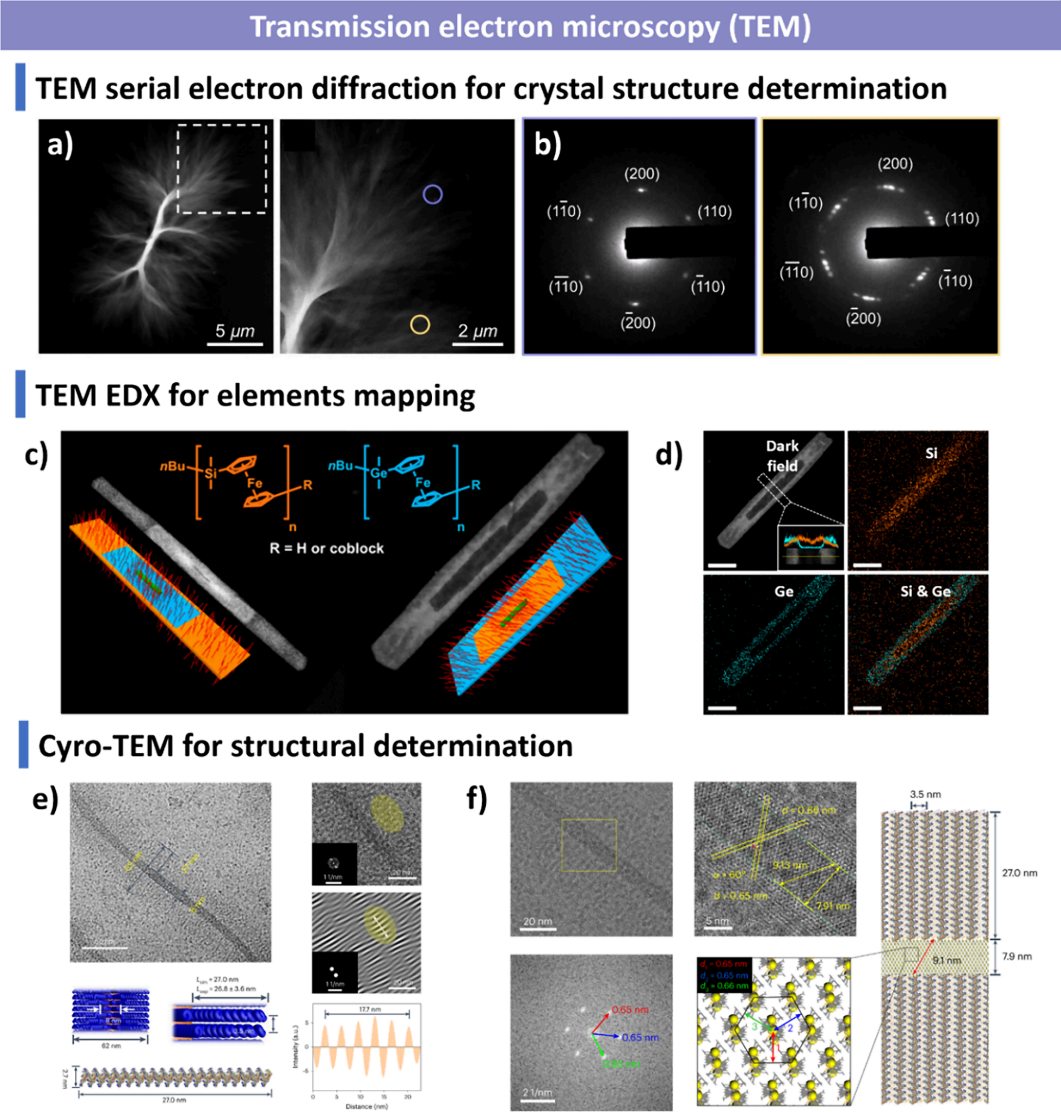
TEM characterization for CDSA assemblies. (a) Annular dark-field STEM images and (b) ED pattern of the PFS_65_-*b*-PI_335_/PFS_56_ micelle using serial electron diffraction (SerialED) tests. Reproduced with permission from ref 350. Copyright 2023 American Chemical Society. (c) Formation of 2D platelets with mixed PFDMS/PFDMG cores grown from 1D PFDMS-*b*-P2VP seeds. (d) STEM image and EDX Si/Ge mapping showing spatial distribution in platelet block comicelles (scale bar: 500 nm). Reproduced with permission from ref 93. Copyright 2017 American Chemical Society. (e) High-resolution cryo-TEM analysis showing corona and core structures of nanofibers. (f) Molecular modeling combined with cryo-TEM revealing core structure of PFS_24_-*b*-P4VP_192_ nanofibers. Reproduced with permission from ref 35. Copyright 2023 Springer Nature.

Energy-dispersive X-ray spectroscopy (EDX) coupled with TEM enables elemental mapping to track the distribution of elements within CDSA assemblies. The Manners group investigated the heteroepitaxial growth of core-forming PFDMS and poly­(ferrocenyldimethylgermane) (PFDMG) block platelets using STEM-EDX.^93^ Elemental mapping revealed an uneven Si distribution, with a higher concentration in the central region, indicating its presence in both the PFDMS core and PDMS corona. In contrast, the Ge map displayed a “hollow” pattern, confirming the absence of Ge in the inner platelet block and highlighting the distinct core-forming domains within the 2D platelet structures (Figure 22c,d).

In recent years, advancements in cryo-TEM have revolutionized the imaging of nanomaterials by preserving their solution-state structures in a vitrified, near-native state, eliminating artifacts from drying. This technique enables direct physical characterization, providing high-resolution real-space images of individual nanostructures. Tian et al. utilized cryo-TEM to examine nanofibers with crystalline PFS cores, revealing both solvated corona chains and the internal crystalline structure (*d*-spacing ∼ 0.65 nm) without surface adhesion interference.^35^ Their findings demonstrated that PFS chains pack into a 2D pseudohexagonal lattice perpendicular to the fiber axis. Based on these observations, they proposed a molecular model for PFS_24_-*b*-P4VP_192_ nanofibers, highlighting the spatial arrangement of coronal chains and a brush density of approximately one P4VP strand per four parallel PFS chains (Figure 22e,f).

Most recently, Lee and co-workers used liquid-phase transmission electron microscopy (LP-TEM) to investigate the dynamic self-assembly of semicrystalline BCPs in aqueous solution.^396^ Through *in situ* visualization, they observed morphological transitions and quantified kinetic parameters, including mean-square displacement, diffusion coefficients, and interparticle interactions. Spherical micelles acted as nucleation seeds for hierarchical crystallization-driven growth. Morphological evolution was governed by the folding and extension of semicrystalline core chains, with lateral assembly minimizing unfavorable core–water interactions. Crystallization-driven hydrophobic interactions promoted long-range aggregation over hundreds of nanometers. Once stabilized, micelles showed no further transitions, even upon collision. The crystallinity of the core-forming blocks strongly influenced intermicellar interactions and self-assembly pathways, with vesicles forming through localized aggregation and crystallization-driven diffusion.

TEM plays a critical role in characterizing CDSA assemblies, providing insights into morphology and crystalline structure. However, due to the intrinsic characteristics of CDSA systems—such as their soft-matter nature, dynamic solution-state behavior, and the coexistence of crystalline and amorphous domains—obtaining comprehensive structural information using conventional TEM remains challenging. In contrast to rigid inorganic nanomaterials, CDSA assemblies are more susceptible to electron beam-induced damage, dehydration artifacts during sample preparation, and potential structural rearrangements under vacuum. Furthermore, the complex interplay between crystalline cores and solvated coronas often limits the level of structural detail achievable with standard TEM imaging. To overcome these limitations and advance the structural understanding of CDSA systems, the application of more advanced electron microscopy techniques is highly encouraged. Spherical aberration-corrected TEM (AC-TEM) offers enhanced spatial resolution and improved image contrast by correcting lens-induced aberrations, enabling more precise visualization of nanoscale features in soft-matter systems. In addition, liquid-phase TEM (LP-TEM) provides a powerful platform for directly observing CDSA dynamics *in situ* under near-native solution conditions, capturing real-time morphological transitions and assembly pathways that are otherwise inaccessible with conventional TEM. The integration of such advanced techniques will be instrumental in driving the next generation of CDSA studies toward deeper mechanistic insights and more accurate structure–property correlations.

#### 4.2.2 Atomic Force Microscopy (AFM)

AFM is a powerful 3D characterization technique that provides high-resolution morphological analysis and accurate height and thickness measurements of nanostructures. With vertical resolution reaching the subnanometer scale (0.1–0.2 nm under optimal conditions), AFM is indispensable for studying nanoscale materials. Beyond imaging, functional attachments such as conductive AFM (C-AFM), force modulation AFM, and nanoindentation allow the measurement of additional properties, including electrical conductivity, adhesion, and mechanical characteristics like Young’s modulus.^446^

AFM has been extensively utilized to characterize self-assembled structures formed via CDSA, including 1D cylindrical micelles and 2D platelets (Figure 23a).^221,380^ By providing detailed insights into morphology, height, and adhesion properties, AFM enables a deeper understanding of the structural organization of CDSA assemblies, essential for optimizing self-assembly conditions and tailoring nanostructures for advanced applications.

**Figure 23:**
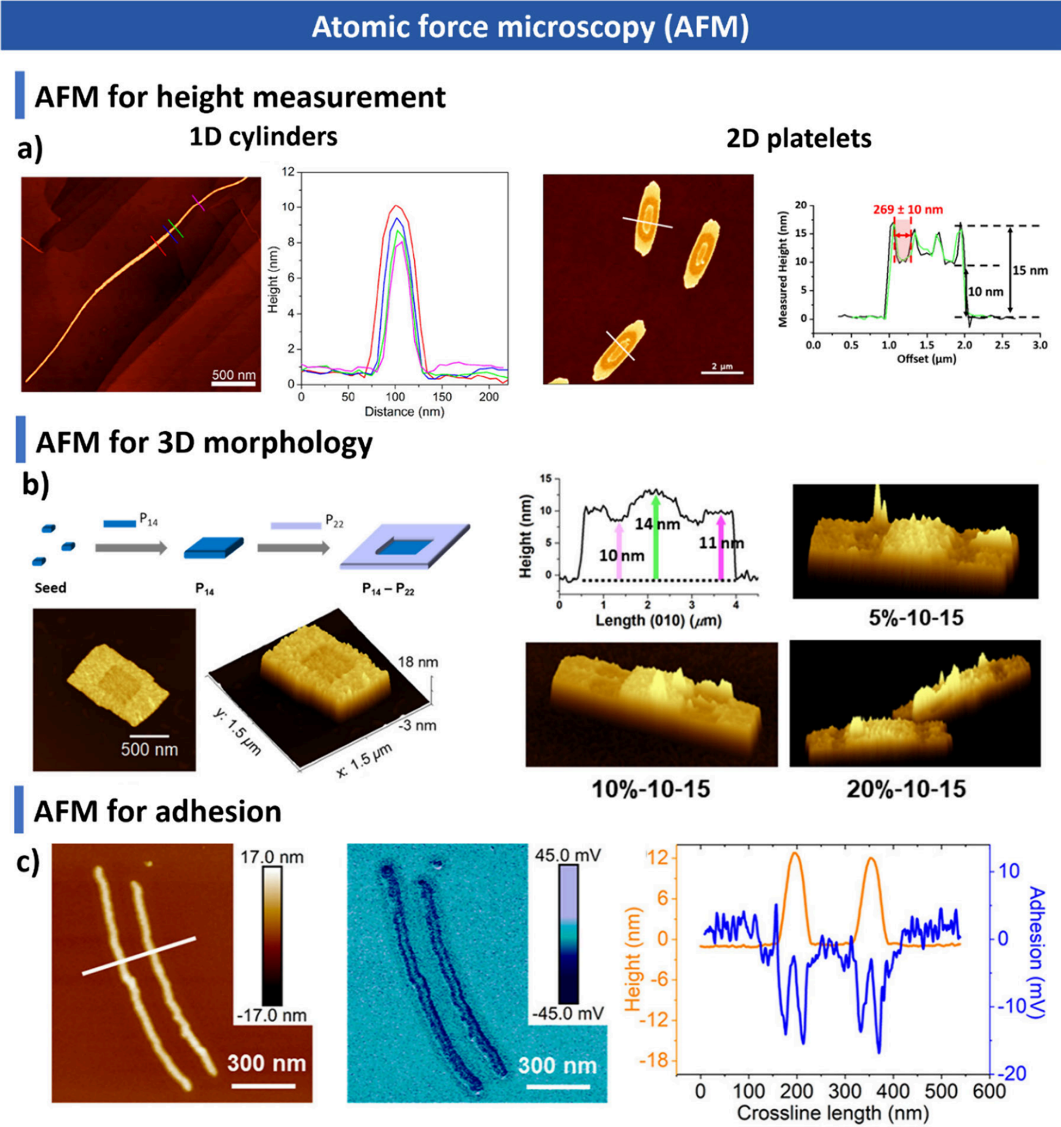
AFM characterization of CDSA assemblies. (a) AFM images and height profiles of 1D cylindrical micelles and 2D platelets. Reproduced with permission from ref 400. Copyright 2015 American Chemical Society. Reproduced with permission from ref 380. Copyright 2023 American Chemical Society. (b) AFM images showing 3D morphologies of assemblies from PCPV homopolymers with different lengths. Reproduced with permission from refs 42, 221 . Copyright 2023 and 2024 American Chemical Society. (c) AFM height and adhesion images of PLLA_47_-*b*-PNIPAm_267_ micelles with controlled lengths. Reproduced with permission from ref 143. Copyright 2019 American Chemical Society.

The folding behavior of polymer chains within crystalline cores can be inferred from AFM height measurements. Li et al. employed AFM to examine the lamellar thickness of polymer single crystals formed under different crystallization conditions.^119^ Their findings showed thicknesses of 5.5, 9, and 12 nm, corresponding to varying degrees of undercooling. By correlating PCL unit cell structure with the degree of polymerization, they determined that polymer chains folded four, two, and one time, respectively. These results demonstrate AFM’s utility in quantitatively probing polymer folding and structural organization within CDSA assemblies.

AFM has been instrumental in imaging the three-dimensional morphology of polymeric nanostructures. Choi and co-workers investigated the self-assembly of conjugated PCPV-based platelets with different DP using AFM, revealing distinct height differences between platelets (Figure 23b).^42,221^ For example, using a seeded growth strategy, they initiated assembly with P_14_ nanorectangles and sequentially added P_22_ unimers to form diblock comicelles. The resulting platelets exhibited a well-defined 3D gully structure, where AFM height analysis revealed a height difference of up to 3 nm between the P_14_ and P_22_ blocks, with the shorter P_14_ block appearing darker in the images. Similarly, the O’Reilly and Dove groups examined crystallization rates in PCL systems with different block lengths.^380^ By combining PCL_22_, PCL_73_, and PCL_51_-*b*-PDMA_189_, they synthesized 3D boat-like platelets, characterized via AFM. These findings highlight AFM’s ability to resolve subtle structural details, such as height variations and morphological transitions, crucial for understanding polymer self-assembly.

Beyond morphological characterization, AFM can be combined with functional imaging modes to probe additional physical properties of CDSA-derived nanostructures. A notable example is the use of tunneling AFM (TUNA) by Manners, Faul, and co-workers to investigate the electronic properties of electroactive fiber-like micelles with crystalline P3HT cores.^447^ In this study, TUNA measurements directly quantified the local electrical conductivity of individual micelles deposited onto device substrates, revealing that the charge transport occurred primarily through the crystalline P3HT core, independent of corona composition.

Additionally, AFM can characterize the adhesion properties of CDSA assemblies. He et al. used AFM to demonstrate that micelles exhibited a uniform core height of 14 nm, attributed to the PLLA core. Adhesion mapping further revealed structural parameters, such as core width (36 nm) and corona width (16 nm), with the latter predominantly lying on the substrate (Figure 23c).^143^ These studies highlight AFM’s ability to capture nanoscale mechanical and interfacial interactions, further enriching the understanding of CDSA assembly behavior.

#### 4.2.3 Fluorescence Microscopy

Fluorescence microscopy serves as a powerful tool for visualizing CDSA-derived nanostructures, complementing traditional techniques such as TEM and AFM. While TEM and AFM provide high-resolution structural details, fluorescence microscopy selectively highlights polymer segments, offering crucial insights into morphological organization and segmental composition. The incorporation of fluorescent labels into block copolymers enables direct identification of distinct domains within self-assembled nanostructures, making fluorescence imaging particularly valuable for investigating segmented micelles, hierarchical nanostructures, and corona-functionalized assemblies.

##### 4.2.3.1 Confocal Laser Scanning Microscopy (CLSM)

CLSM enhances fluorescence imaging by eliminating out-of-focus light, significantly improving spatial resolution. Unlike conventional wide-field fluorescence microscopy, which illuminates the entire sample simultaneously, CLSM employs a focused laser beam that scans the specimen point by point, combined with a pinhole aperture to generate high-contrast, three-dimensional reconstructions of nanostructures. This technique effectively visualizes segmented micelles, block comicelles, and patchy platelet assemblies, enabling detailed analysis of their morphological organization.

CLSM imaging of A–B–A segmented nanofibers confirmed the presence of distinct fluorescently labeled segments. The central B-segment (PDHF-*b*-P3EHT crystalline core) exhibited red/orange P3EHT emission, while the longer A-segments (PDHF crystalline core) displayed blue fluorescence, directly visualizing the segmented nature of CDSA-derived micelles (Figure 24a).^229^ Similarly, CLSM imaging of 2D patchy and hollow rectangular platelets confirmed selective corona functionalization with fluorescent PFS BCPs or nanoparticle associations, allowing high-contrast visualization of complex micellar morphologies (^344^ The ability to resolve structurally distinct domains within self-assembled nanostructures highlights the advantages of fluorescence-based characterization.

**Figure 24:**
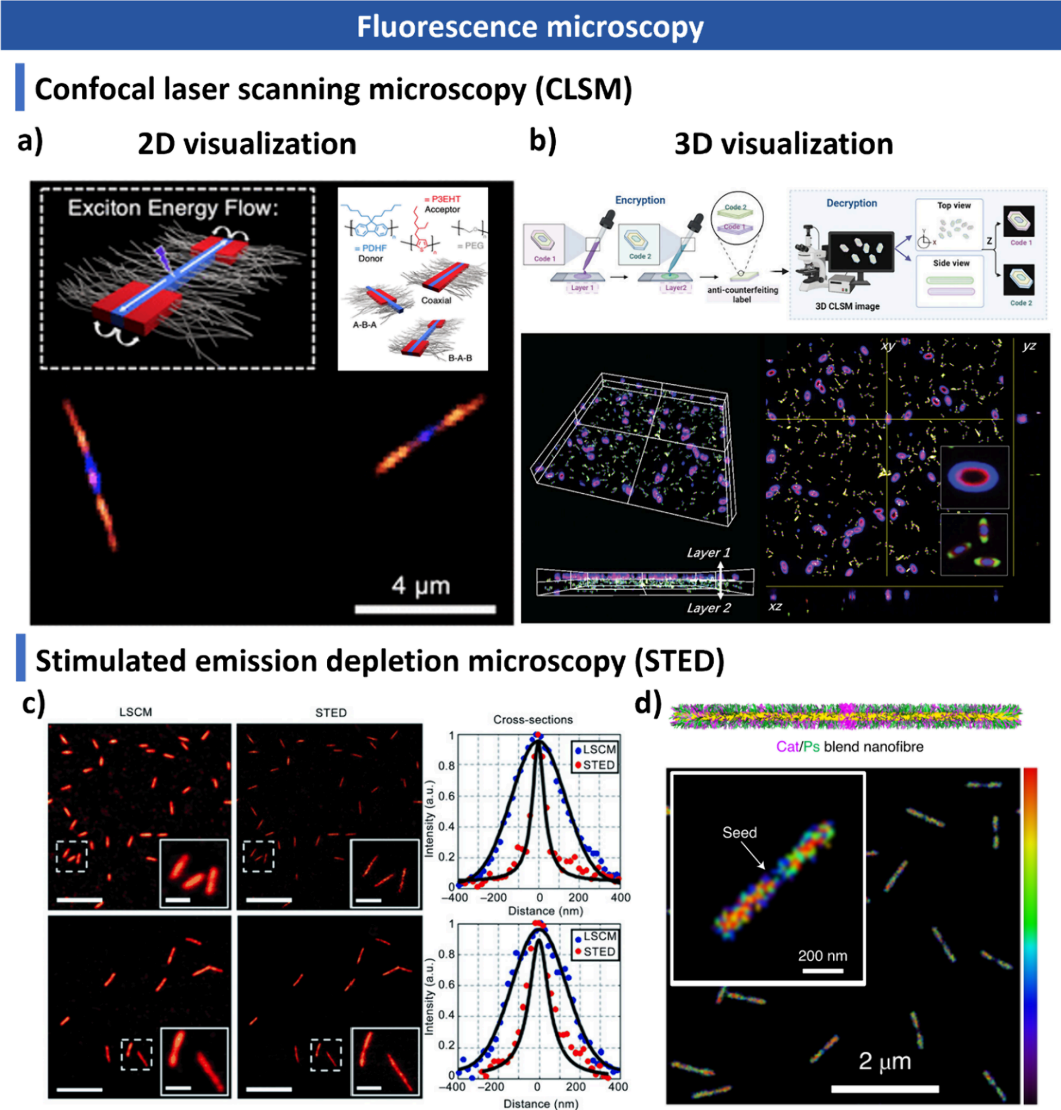
Fluorescence microscopy characterization of CDSA assemblies. (a) CLSM image of B–A–B segmented nanofibers formed by epitaxial growth of PDHF_8_-*b*-P3EHT_25_-*b*-PEG_113_ unimers from PDHF_14_-*b*-PEG_227_ seed micelles in THF:MeOH (1:9, v/v). Reproduced with permission from ref 229. Copyright 2020 American Chemical Society. (b) Design of a two-layered CDSA-based anticounterfeiting film with encoded barcodes. Reproduced with permission from ref 114. Copyright 2023 Wiley-VCH GmbH. (c) CLSM and STED images of cylindrical micelles labeled with STAR635 and CAGE635. Reproduced with permission from ref 448. Copyright 2015 Wiley-VCH GmbH. (d) STED images of Cat/Ps blend nanofibers prepared using a PFS-*b*-Cat to PFS-*b*-Ps mass ratio of 1:10. Reproduced with permission from ref 449. Copyright 2020 Springer Nature.

CLSM was employed in layered scanning mode to reconstruct 3D images of fluorescence-encoded CDSA barcode particles, enabling depth-resolved visualization of encrypted structures. Microscale barcodes with predefined fluorescence patterns were evenly distributed within a polymer film, with no overlap, ensuring clear spatial separation of encoded information.^114^ The strong crystallization force within the CDSA system stabilized the platelets, preventing fluorophore leakage or structural disassembly. To enhance security, a two-layered PVP film containing distinct fluorescence-encoded CDSA barcodes was fabricated. CLSM 3D imaging successfully resolved the layered structures, offering clear visualization of encrypted data at defined depths (Figure 24b). This depth-resolved fluorescence imaging approach provides a novel strategy for applying CDSA-derived barcode particles in advanced anticounterfeiting and secure labeling technologies.

##### 4.2.3.2 Stimulated Emission Depletion Microscopy (STED)

STED microscopy is a super-resolution fluorescence imaging technique that overcomes the diffraction limit of conventional optical microscopy, achieving nanometer-scale resolution. By selectively depleting fluorescence emission in a controlled manner, STED reduces the size of the effective point spread function, enhancing spatial resolution beyond the capabilities of standard confocal microscopy. This technique has been applied to investigate block copolymer nanostructures with high precision, providing structural details that bridge the resolution gap between fluorescence microscopy and electron microscopy.

Although CLSM captures micelle dimensions and segmental organization in CDSA-derived structures, its resolution is inherently limited. STED imaging demonstrates agreement with TEM measurements while enabling multicolor fluorescence visualization in solution, allowing for accurate size determination and polymer domain distribution mapping within self-assembled micelles (Figure 24c).^448^ Selective fluorophore incorporation into block copolymers enables direct observation of segmental organization, revealing the internal structure of CDSA assemblies with unprecedented resolution.

STED microscopy has also been employed to analyze micelle elongation and growth kinetics in CDSA. Fluorescently labeled PFS_56_-*b*-PDMS_775_ block copolymers were used to track micelle growth by adding fluorescent unimers to preformed seed micelles, allowing real-time visualization of micelle elongation.^394^ Dual-color fluorescence labeling distinguished newly incorporated unimers from pre-existing seed micelles, elucidating the mechanisms governing CDSA growth. This high-resolution imaging approach provides crucial insights into the kinetics of CDSA-derived structures, offering a quantitative framework for understanding the CDSA process.

Additionally, STED was applied to probe the spatial distribution of photosensitizers in a blend of nanofibers containing catalytic and photoactive segments.^449^ The fluorescence of the photosensitizer block copolymer was confined to the terminal regions, with negligible emission near the cobalt-rich seed, demonstrating segmental distribution at the nanoscale (Figure 24d). This detailed localization of functional polymer domains highlights the potential of STED in investigating molecular organization within CDSA assemblies.

Fluorescence microscopy, when integrated with traditional CDSA characterization techniques such as TEM and AFM, provides a comprehensive approach to structural analysis. Selective fluorescence labeling differentiates polymer domains, while high-resolution imaging techniques such as CLSM and STED offer detailed insights into nanostructure composition. Expanding the application of fluorescence-based techniques to study optoelectronic properties and responsive nanostructures will further enhance the understanding of CDSA. The continued advancement of fluorescence microscopy techniques in CDSA characterization will enable more refined structural analysis, leading to improved design strategies for functional self-assembled materials. Multicolor imaging and super-resolution microscopy are expected to refine structural mapping, facilitating the controlled fabrication of CDSA-derived nanostructures for applications in materials science and nanotechnology.

#### 4.2.4 Interferometric Scattering (iSCAT) Microscopy

iSCAT microscopy is an advanced imaging technology that surpasses traditional characterization methods in nanoparticle analysis.^441^ Unlike conventional techniques, iSCAT utilizes light scattering for label-free imaging, offering ultrahigh-speed real-time detection and accurate nanoparticle positioning. Its exceptional sensitivity enables single-molecule-level detection, making it a powerful tool for nanoscale studies. A key advantage of iSCAT is its minimal dependence on particle size, as it measures the interference between scattered light from nanoparticles and a reference beam in the same optical path. This capability ensures superior sensitivity and signal-to-noise ratio compared to fluorescence microscopy.^450^

The working principle of iSCAT involves illuminating a sample with coherent laser light through a high numerical aperture objective lens. The interaction generates both scattered light from the sample and reflected light from the substrate, which are simultaneously captured by a detector. Since iSCAT relies on interference rather than fluorescence, it is unaffected by photobleaching, scintillation, or light saturation. Its straightforward system setup, compatibility with fluorescence microscopy, and ease of sample preparation make iSCAT a versatile and scalable technique.^451^ In nanoparticle characterization, iSCAT offers unparalleled advantages in single-particle tracking and positioning due to its exceptional spatial precision and time resolution. By utilizing increased laser power to shorten exposure times, iSCAT enables the study of dynamic self-assembly processes with high temporal resolution, making it particularly suitable for applications requiring continuous monitoring.^452,453^

More recently, Wallace, O’Reilly, and co-workers employed iSCAT microscopy to investigate the real-time growth dynamics of nanostructures formed via CDSA, including PCL-based 1D fibers and 2D platelets.^384^ This technique facilitated the mapping of key parameters such as growth rates, size distributions, and morphological evolution, yielding critical insights into the kinetics and mechanisms governing self-assembly. iSCAT analysis demonstrated that PCL-based systems exhibited significantly faster assembly kinetics in both 1D and 2D structures compared to PFS-based fibers and PCPV-based nanosheets. Additionally, iSCAT captured early stage CDSA evolution, where particle contrast variations served as real-time indicators of nucleation and growth dynamics.

Beyond kinetic studies, iSCAT provided a powerful contrast mechanism for visualizing multiannular platelet formation through sequential unimer addition, enabling spatially resolved mapping of polymer composition within individual platelets. This capability underscores the potential of iSCAT in advancing the mechanistic understanding of CDSA by elucidating kinetic pathways and spatial organization in hierarchical nanostructures with unprecedented temporal and spatial resolution.

### 4.3 Spectroscopy

Spectrum characterization techniques, widely used for molecular structure determination, have also been extensively applied to nanostructure analysis due to their easy accessibility, high sensitivity, and nondestructive nature.

UV–vis spectroscopy enables rapid detection, making it highly beneficial for monitoring the CDSA process with fast kinetics.^37,145,380^ The rapid growth kinetics of PCL-based platelets during living CDSA pose a challenge for real-time monitoring. To address this, the O’Reilly and Dove groups employed UV–vis spectroscopy to track the process.^380^ This approach relies on the principle that the increasing nanoparticle size enhances light scattering, resulting in reduced transmitted light intensity, which is quantified as increased absorption in the spectrum. When homopolymers of varying lengths were used as unimers for epitaxial growth, the polymer with the longest PCL chain exhibited the fastest growth kinetics, with the entire process completed within just 2 min (Figure 25a,b), which could be well captured by UV–vis spectroscopy.

**Figure 25:**
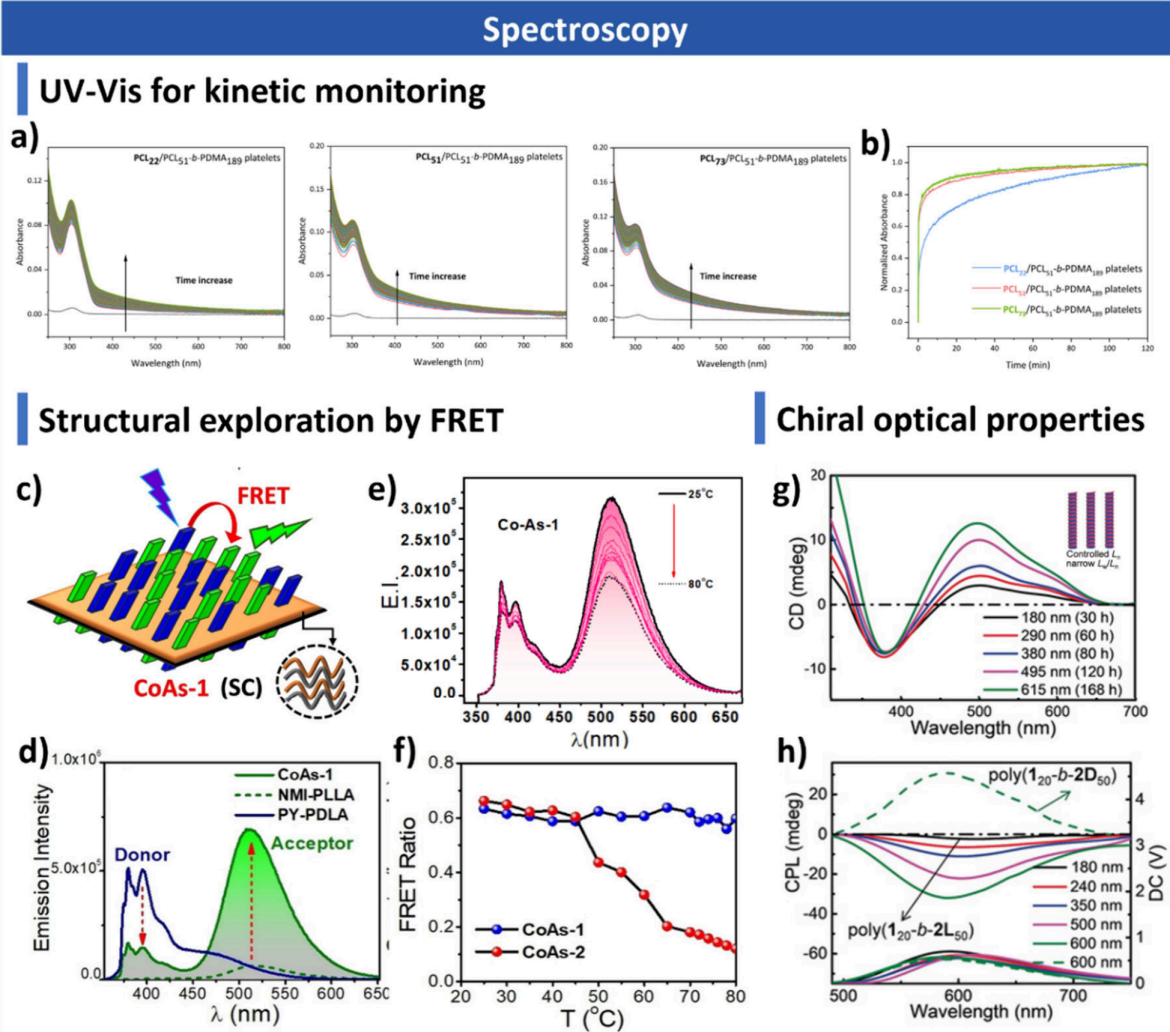
Application of spectrum in characterization of CDSA nanoparticles. (a) UV–vis spectra (250–800 nm) tracking changes in absorbance during CDSA. (b) CDSA kinetics monitored by UV–vis absorbance at 500 nm. Reproduced with permission from ref 380. Copyright 2024 American Chemical Society. (c) Schematic of FRET on platelet surfaces. (d) Emission spectra of polymers and coassembled platelets. (e) Temperature-dependent emission spectra of coassembled platelets. (f) FRET ratio as a function of temperature. Reproduced with permission from ref 455. Copyright 2023 Wiley-VCH GmbH. (g) CD spectra of helical micelles with different lengths prepared by seeded growth with varying annealing times. (h) CPL spectra of nanofibers with different lengths. Reproduced with permission from ref 193. Copyright 2020 Wiley-VCH GmbH.

Further to this, the Das group employed fluorescence emission spectroscopy to investigate chiral platelet nanostructures functionalized with chromophores through end-group modification of PLA polymers.^454–456^ By grafting naphthalene monoamide (NMI) onto PLLA and pyrene (PY) onto PDLA, they designed a Förster resonance energy transfer (FRET) system to probe the coassembly of stereoisomeric platelets (Figure 25c,d).^455^ The constant high FRET efficiency observed in the coassembled platelets demonstrated that the close spatial proximity of chromophores was induced by strong stereoisomeric interactions during the assembly process (Figure 25e,f). This study highlights the utility of fluorescence-based techniques for elucidating the self-assembly mechanism and chiral organization in anisotropic nanoparticles.

Circular dichroism (CD) and circularly polarized luminescence (CPL) spectroscopies are powerful tools for characterizing the chirality and structural evolution of self-assembled nanoparticles.^457^ Wu and co-workers utilized these techniques to investigate the formation of chiral nanofibers assembled from poly­(3-hexylthiophene)-*block*-poly­(phenyl isocyanide)­s (P3HT-*b*-PPI).^193^ CD measurements revealed that the nanofibers’ chirality arose from their self-assembled structure, with the increasing CD signal during annealing indicating that chirality developed progressively along with epitaxial growth. This trend suggests that CD spectroscopy can serve as an effective tool for monitoring the structural evolution of nanofibers in real-time. Consistently, circularly polarized luminescence (CPL) measurements showed that the CPL intensity increased with the nanofiber length, as the chirality was transferred from the PPI block to the emissive P3HT block during growth. The combined use of CD and CPL spectroscopies not only provides complementary information on ground-state and excited-state chiral properties but also enables comprehensive characterization of the structural and optical features of self-assembled nanoparticles (Figure 25g,h).

Spectroscopy techniques offer valuable insights into the structural and optical properties of self-assembled nanoparticles, making them essential tools for comprehensive nanoparticle characterization. In addition to the aforementioned techniques, Fourier transform infrared spectroscopy (FTIR) has been widely applied as well.^37,145^ By combining these spectroscopic methods with scattering techniques and microscopic imaging, a multidimensional understanding of nanoparticle morphology, molecular organization, and optical properties can be achieved. This integrated approach not only facilitates the detailed characterization of nanostructures but also provides critical insights into the structure–function relationship, paving the way for the rational design of functional nanoparticles.

To provide a clearer overview of the strengths and limitations of different characterization techniques in the context of CDSA, we summarize in Table 4 key features, common limitations, and typical application scenarios for each method. This comparative guide is intended to assist researchers in selecting suitable approaches for specific types of CDSA assemblies and experimental conditions.

**Table 4:** Summary of Key Characterization Techniques Used to Investigate CDSA Mechanisms, Highlighting Their Key Features, Limitations, and Typical Application Scenarios

**Characterization techniques**	**Key features/advantages**	**Limitations**	**Suitable situation**
**Scattering techniques**			
DLS	Measures hydrodynamic size (*R*_h_) via Brownian motion; rapid and convenient; widely used for size monitoring and kinetic studies	Cannot resolve morphology; *R*_h_ assumes spherical model	Monitoring size evolution; screening assembly conditions; kinetic studies
SLS	Provides molecular weight (*M*_w_), aggregation number, radius of gyration (*R*_g_), particle shape; sensitive to morphology and aggregation	Complex measurements; requires multiple concentrations and angles	Determining shape and size of rigid nanoparticles; studying aggregation behavior
SAXS	Probes nanostructures (1–100 nm); provides size, shape, internal packing; enables real-time monitoring	Requires synchrotron or advanced source; complex data fitting	Analyzing internal chain arrangement; tracking assembly kinetics; morphology studies
WAXS	Probes atomic-scale crystalline structure; resolves polymorphs and crystal packing; tracks crystallization	Data interpretation can be complex	Determining crystalline core structures; tracking crystallization progress
SANS	Provides unique contrast for hydrogen-rich materials; sensitive to internal chain organization and solvent–polymer interactions	Limited neutron source availability; complex data analysis	Studying internal chain organization; solvent–polymer interactions
**Microscopy**			
TEM	High-resolution imaging of morphology; SAED provides lattice structure information	Requires vacuum (except Cryo-TEM); possible drying artifacts	Imaging morphology; crystalline structure; hierarchical assemblies
AFM	Provides 3D surface topology; height and thickness measurements; subnm vertical resolution	Surface method; limited throughput; sensitive to substrate effects	Analyzing platelet thickness; chain folding; conductivity and adhesion properties
Fluorescence microscopy	Visualizes fluorescently labeled segments; enables 3D imaging and segmental mapping; live imaging with depth profiling (CLSM/STED)	Limited resolution (improved with STED); requires fluorescent labeling	Mapping segmental structures; barcodes; dynamic growth tracking
iSCAT	Label-free imaging; ultrahigh-speed, real-time single-particle tracking; no photobleaching	Advanced optical setup; sensitive to surface effects	Tracking growth process; spatial mapping of composition
**Spectroscopy**			
UV–vis, CD, CPL, FTIR, etc.	Monitors optical transitions, chirality, bonding vibrations; high sensitivity; fast kinetics monitoring; detects chiral organization	No direct imaging; complementary to microscopy/scattering	Monitoring growth kinetics; chiral organization; polymer interactions

## 5 Theoretical Simulation for Revealing CDSA Mechanisms

Theoretical simulations have become an indispensable tool for understanding CDSA mechanisms, complementing experimental approaches.^458^ Advances in computational power enable the study of complex material behaviors across multiple scales, from atomic-level interactions to mesoscopic dynamics.^459^ These methods not only elucidate fundamental self-assembly processes but also provide predictive models, significantly reducing experimental time and cost.^309,460,461^ In soft matter science, where hierarchical structures and dynamic processes span multiple length scales, simulations such as Monte Carlo methods, Brownian dynamics, and dissipative particle dynamics offer unique insights into self-assembly phenomena (Table 5).^131,462–464^ These techniques have been instrumental in deciphering the intricate kinetics and morphology transitions in CDSA, providing a deeper mechanistic understanding of nucleation, growth, and structural evolution.

**Table 5:** Representative Examples of Simulations Applied to Study the CDSA Process

**Item**	**Simulation method**	**Model**	**Dimensions**	**Reference**
Polymer crystallization in dilute solution	Monte Carlo	Bond fluctuation model	-	^465^
Chain folding in polymer melt crystallization	Monte Carlo	Microrelaxation model	-	^466^
Single crystals from polymer solution	Monte Carlo	Anisotropic aggregation model	2D	^467^
Lamellar crystals in dilute solutions	Monte Carlo	Lattice polymer-chain model	2D	^468^
Fibril crystal growth of diblock copolymer	Monte Carlo	Lattice polymer-chain model	1D	^469^
Effect of solvent selectivity on crystallization-driven fibril growth	Monte Carlo	Lattice polymer-chain model	1D	^470^
Crystallization-driven 2D self-assembly of amphiphilic PCL-*b*-PEO coated gold nanoparticles	Dissipative Particle Dynamics	Coarse-grained model	2D	^131^
Glyco-based platelets via CDSA	Dissipative Particle Dynamics	Coarse-grained model	2D	^166^
Living supramolecular polymerization of rod–coil block copolymers	Brownian Dynamics	Coarse-grained model	1D	^471^
Growth and termination of cylindrical micelles via liquid CDSA	Brownian Dynamics	Coarse-grained model	1D	^463^
Anchorage-dependent living supramolecular self-assembly of polymeric micelles	Brownian Dynamics	Coarse-grained model	1D	^238^
Fusion growth of two-dimensional disklike micelles	Brownian Dynamics	Coarse-grained model	2D	^464^
2D liquid CDSA of rod–coil block copolymers	Brownian Dynamics	Coarse-grained model	2D	^460^
Hierarchical 2D–1D micelles self-assembled from the heterogeneous seeded-growth of rod–coil block copolymers	Brownian Dynamics	Coarse-grained model	Complexed 2D-1D	^461^
Living growth kinetics of polymeric micelles on a substrate	Brownian Dynamics	Coarse-grained model	1D	^242^

### 5.1 Monte Carlo Method

The Monte Carlo method is a powerful computational approach for simulating complex systems, offering unique advantages in studying self-assembly. Its stochastic nature allows exploration of diverse configurational landscapes, capturing intricate dynamics and statistical behaviors of molecular assemblies. By modeling interactions and growth pathways, Monte Carlo simulations provide crucial insights into nucleation, growth kinetics, and morphological transitions.

In the early stages of crystallization, direct experimental observation remains challenging, making simulations an invaluable tool for tracking intramolecular nucleation-controlled mechanisms governing crystal growth. The Hu group employed Monte Carlo simulations to examine how anisotropic driving forces, confinement effects, and kinetics influence the quasi-1D fibril crystal growth of diblock copolymers under feeding and depleting conditions.^469^ Their results highlighted the essential role of anisotropic driving forces in sustaining steady fibril crystal growth. Below a critical concentration, lamellar crystal width was restricted by the noncrystalline block, with this threshold decreasing as the fraction of the noncrystallizable block reduced. In the depleting mode, early stage fibril growth followed an exponential decay in long-axis dimensions over time, with growth rates decreasing linearly with polymer concentration, paralleling behavior in the feeding mode (Figure 26a,b).

**Figure 26:**
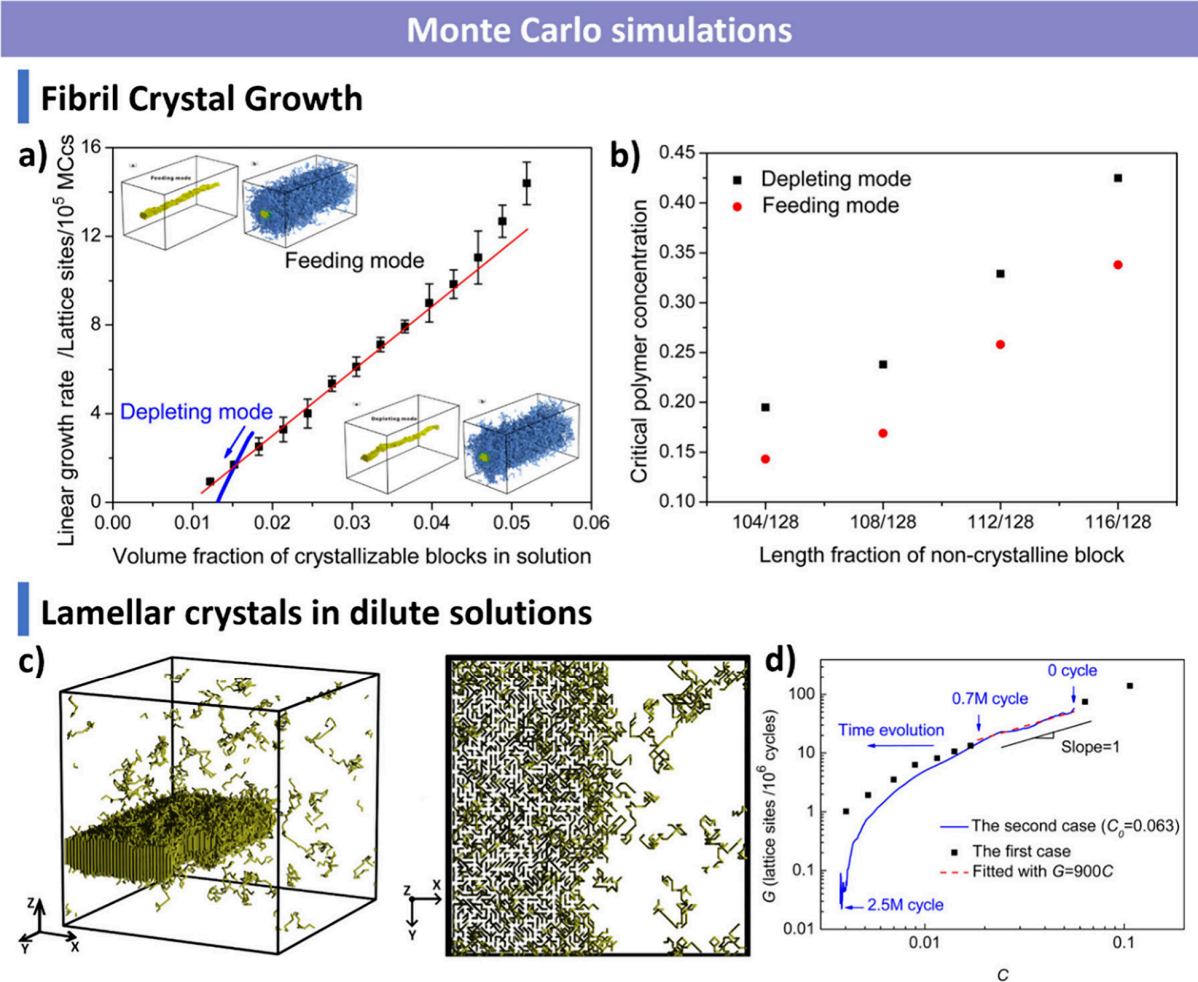
Monte Carlo Simulations. (a) Comparison of linear crystal growth rates versus crystallizable block volume fractions in feeding and depleting modes. (b) Critical volume fractions of diblock copolymers with different noncrystalline block ratios in both modes. Reproduced with permission from ref 469. Copyright 2015 American Chemical Society. (c) Simulation snapshots showing lamellar crystal growth along the *X*-axis from two angles. (d) Double-logarithmic plots of linear growth rates versus polymer volume fractions for two different cases. Reproduced with permission from ref 468. Copyright 2013 American Chemical Society.

Hu et al. employed dynamic Monte Carlo simulations to investigate the kinetics of quasi lamellar crystal growth in polymer solutions under two different scenarios.^468^ In the first scenario, where a few small crystals grew in a large bulk solution with a nearly constant polymer concentration, growth rates exhibited distinct concentration-dependent behaviors. At higher concentrations, the kinetic equation was dominated by the driving-force term, resulting in a linear dependence of crystal growth rates on concentration (Figure 26c). At lower concentrations, the nucleation-barrier term became significant, causing nonlinear deviations. In another scenario, where numerous crystals grew in a confined solution with depleting polymer concentrations, growth rates decayed over time (Figure 26d). However, in the early stage, the rates followed the same linear concentration-dependent behavior observed in the first case. Notably, the crystal growth size at this stage adhered to an exponential decay function over time, providing a theoretical framework for interpreting experimental data.

### 5.2 Brownian Dynamics

The Brownian dynamics (BD) method is widely employed to simulate self-assembly processes, capturing thermal fluctuations and hydrodynamic interactions to reveal kinetic pathways, nucleation events, and structural transitions. Its ability to model stochastic molecular motion makes it particularly useful for investigating CDSA-related crystallization dynamics.

In a study of 1D systems, Boott et al. reported detailed kinetic behavior for the growth of fiber-like PFS micelles, where the growth rate was best fitted to a stretched exponential form.^394^ They also demonstrated that the growth rate was highly sensitive to the PFS block length in the block copolymer. Motivated by these experimental observations, Gao et al. utilized BD simulations to explore the kinetics of living supramolecular polymerization using rod–coil block copolymers as a model system.^471^ By extending classical kinetic theories with an assumed length-dependent rate coefficients, they showed that cylindrical micelles exhibit decreasing growth rates as they elongate due to their rigidity and diffusion behavior. The simulations revealed rapid unimer consumption during early growth stages, followed by a gradual decline, with shorter micelles exhibiting faster unimer uptake (Figure 27a). It is important to note that these simulation results are based on the assumption of length-dependent propagation rates introduced into the model; while this approach successfully reproduced key features of the experimental trends, it does not independently confirm that such length dependence inherently exists in CDSA systems.

**Figure 27:**
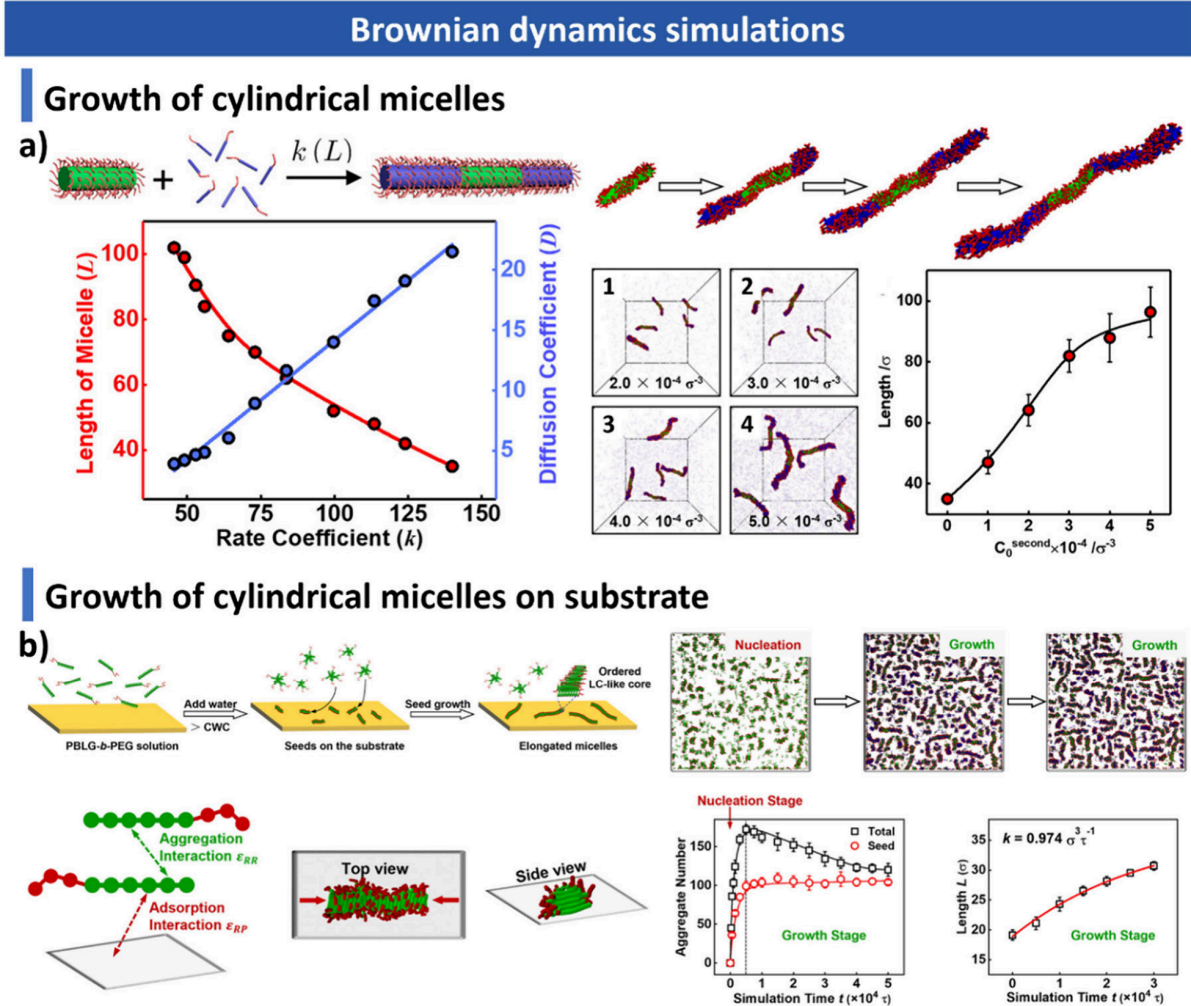
Brownian dynamics simulations for 1D nanostructures formed via CDSA. (a) Simulation of cylindrical micelle growth via living CDSA. Reproduced with permission from ref 471. Copyright 2019 American Chemical Society. (b) Simulations of seeds’ formation and subsequent micelle growth on a substrate. Reproduced with permission from ref 242. Copyright 2024 American Chemical Society.

Beyond free-floating systems, substrate-confined growth presents another important aspect of CDSA. The Lin group developed a BD-based theoretical model to elucidate the kinetics of cylindrical micelle growth on surfaces.^242^ Their study identified two distinct growth stages: an initial nucleation stage where small micelle seeds form, and a subsequent elongation stage where aggregates from solution fuse with the seed termini. During this fusion process, disordered rod blocks within newly formed aggregates gradually rearrange to align with the ordered packing of the micelle core (Figure 27b). This continuous fusion-rearrangement mechanism progressively consumes free aggregates, yielding well-ordered, elongated cylindrical micelles with high structural uniformity and liquid crystal-like core arrangements.

BD simulations extend beyond 1D CDSA, offering insights into the interplay between diffusion dynamics and edge reactivity in 2D disklike micelle growth. Gao and co-workers employed BD simulations to investigate the 2D living growth of disklike micelles formed via liquid CDSA of rod–coil block copolymers.^460^ Their study focused on the seeded growth mechanism, where unimers attach to the edges of disk-like seeds with smectic-like liquid-crystalline cores. The simulations revealed a dynamic balance: larger disks diffuse more slowly, reducing growth rates, yet their increased reactive edge sites promote unimer addition (Figure 28a). This interplay leads to self-similarity in growth, where core-forming rod blocks undergo chain rearrangement, maintaining ordered packing within the micelle core.

**Figure 28:**
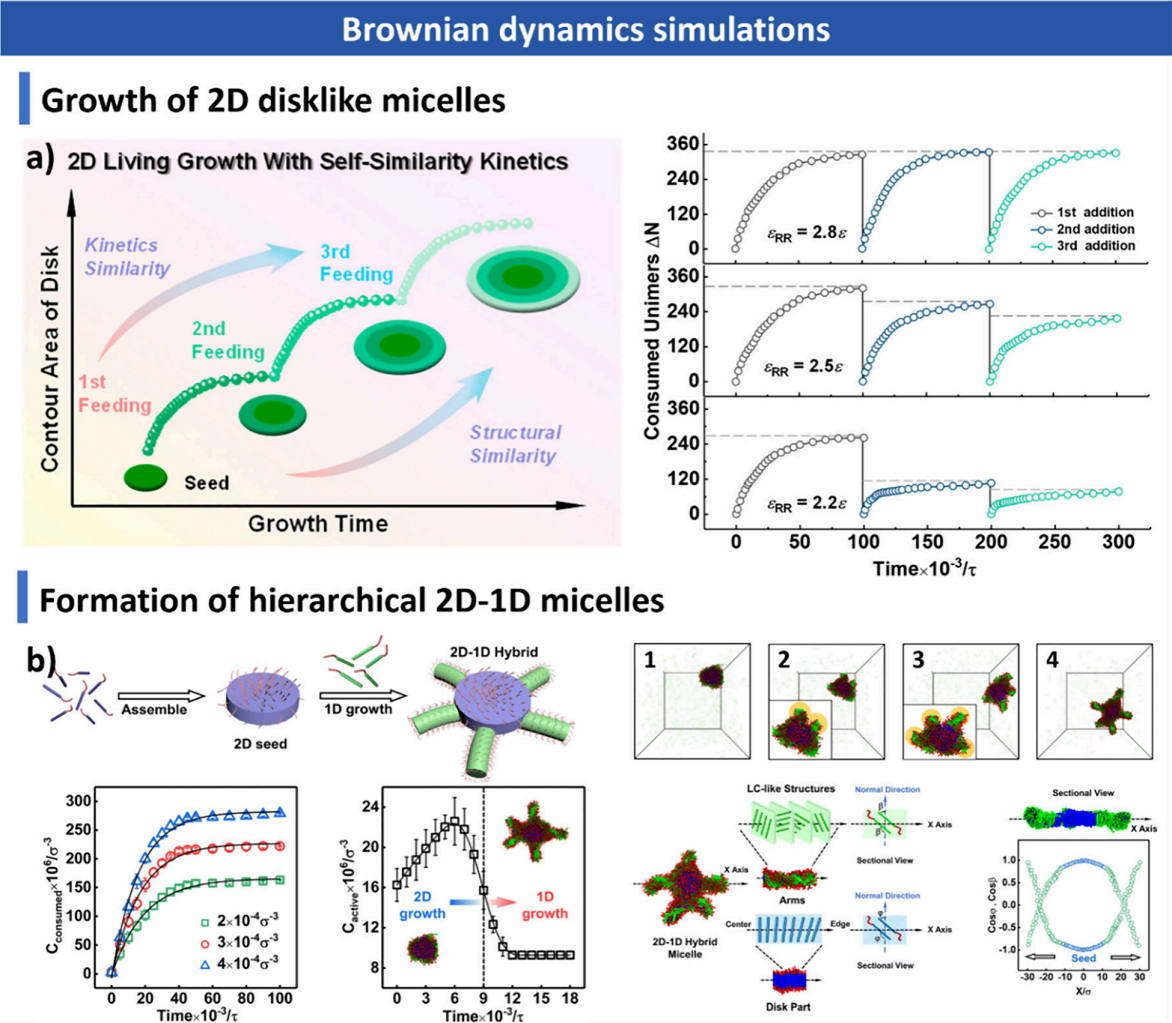
Brownian dynamics simulations for 2D nanostructures formed via CDSA. (a) Simulation snapshots showing multistep growth of disk-like micelles. Reproduced with permission from ref 460. Copyright 2022 American Chemical Society. (b) Simulation of hybrid 2D–1D nanostructure formation via seeded growth of R6C3 copolymers. Reproduced with permission from ref 461. Copyright 2023 Royal Society of Chemistry.

BD simulations have also been employed to explore the formation of hierarchical hybrid micelles. The Lin group simulated the seeded growth of 2D–1D (disk–cylinder) hybrid micelles formed via LCDSA (liquid crystal CDSA) of rod–coil block copolymers.^461^ The disk seed cores exhibit smectic-like LC ordering, while cylindrical arms adopt cholesteric LC packing. Growth occurs through heterogeneous nucleation on disk seeds, followed by homogeneous epitaxial elongation of cylindrical arms. Kinetic analysis revealed that the number of arms correlates with the disk seed size, while unimer concentration dictates total arm length (Figure 28b). Additionally, steric hindrance influences regioselective arm formation, resembling substitution rules in aromatic chemistry. Growth preferentially occurs at para positions relative to initial arms, followed by meta and ortho positions.

### 5.3 Other Simulation Methods

Beyond traditional approaches, dissipative particle dynamics (DPD) simulations have emerged as a valuable tool for investigating CDSA, offering molecular-level insights into self-assembly mechanisms. Chen and co-workers applied DPD to study the solvent-dependent assembly behavior of glycopolymers, specifically coarse-grained PLLA_33_-*b*-PMan_12_, under varying DMF/water conditions.^166^ In pure water, PLLA’s hydrophobicity led to rapid aggregation into small clusters, which fused into stable spherical micelles with PLLA cores and glycopolymer coronas. In DMF-containing solutions, assembly initially formed spherical micelles, but as temperature decreased, a shape transition to columnar micelles occurred. This transformation was attributed to the reduced PLLA–solvent incompatibility and decreased conformational entropy, enhancing PLLA–PLLA interactions. DPD simulations revealed that spherical micelles promoted 1D growth, while columnar micelles facilitated 2D growth, with simulated dimensions (∼10 nm) aligning well with experimental findings.

In addition to dynamic simulations such as DPD, molecular packing and crystallization behavior can also be investigated by atomistic simulations. Recently, Feng, and co-workers employed Materials Studio 7.0 using the COMPASS (condensed-phase optimized molecular potentials for atomistic simulation studies) force field to model the packing of donor–acceptor (D–A) π-conjugated units within CDSA-derived nanofibers.^209,213^ These studies revealed how subtle variations in molecular structure, such as side chain length and core electronic effects, influenced the intermolecular packing, π–π stacking distance, and consequently the self-assembly behavior and optical properties of the resulting nanostructures. This highlights the power of combining atomistic simulations with CDSA experiments to probe structure–property relationships at the molecular level and to guide the rational design of functional nanomaterials.

Integrating theoretical simulations into CDSA research has substantially advanced the mechanistic understanding of self-assembly kinetics and morphological evolution. Techniques such as Monte Carlo, Brownian dynamics, and DPD simulations offer powerful tools for probing nucleation processes, growth pathways, and morphology transitions across a wide range of conditions. These methods enable detailed and systematic evaluation of factors such as concentration effects, solvent composition, polymer–polymer and polymer–solvent interactions, which are often challenging to isolate experimentally. Simulations also provide a predictive framework to explore the assembly of complex hierarchical structures in 1D, 2D, and hybrid nanoscale systems, complementing and extending experimental observations.

However, despite their strengths, it is important to recognize that simulations often require the introduction of simplifying assumptions or predefined parameters (e.g., interaction potentials, rate coefficients, chain rigidity) to make the models computationally tractable. These assumptions may not always fully capture the complexities of experimental systems, and model limitations can lead to deviations between simulated and experimental results. Therefore, careful validation and iterative refinement of simulation parameters against experimental data are essential to ensure the reliability and relevance of predictive modeling. A synergistic approach, wherein simulations guide experimental design and experimental results inform the development of more accurate models, will be critical for achieving a truly comprehensive understanding of CDSA mechanisms.

## 6 Design and Applications of CDSA Assemblies

CDSA-derived nanostructures offer controlled morphological features, enabling applications across biomedicine, optics, energy conversion, and catalysis.^25^ By tailoring size, shape, and functional properties, these assemblies facilitate advanced functionalities, from targeted drug delivery to high-efficiency optoelectronic materials.^1,28,241^

### 6.1 Surface Engineering

The surface of nanoparticles plays a pivotal role in determining their stability, interactions, and functionality across various applications.^472–475^ Surface engineering of assembled nanoparticles typically involves either premodification of building blocks with functional groups before self-assembly or postmodification of the assembled structures.^474^ In CDSA, where crystallization governs the assembly process, local modifications with small functional groups that minimally disrupt crystallization generally have little impact on assembly behavior. Common strategies include end-group modification and copolymerization with a small fraction of functional moieties in the corona block to introduce specific functionalities.^111,132,454–456,476^ For example, the O’Reilly and Manners groups incorporated fluorescent dyes into polymers to generate diverse fluorescent patterns in 1D and 2D nanostrucutres,^89,114^ while Finnegan et al. conjugated block copolymers with biosensors to enhance the cell affinity of assembled nanorods.^259^ While these modification approaches are commonly applied in other self-assembly systems, CDSA presents unique opportunities for surface functionalization. The following section will focus on modification strategies specifically relevant to CDSA.

To achieve controlled surface patterning, CDSA-derived platelets are typically formed through the coassembly of homopolymers and block copolymers, with their spatial distribution dictating the final surface morphology. The Manners group fine-tuned the unimer composition to modulate the crystallization kinetics of homopolymers and diblock copolymers.^378^ This approach allowed controlled manipulation of P2VP corona distribution, enabling uniform dispersion or localization at the platelet center or edges with each unimer addition. Through programmed design, sequential unimer additions facilitated the fabrication of diverse surface patterns, demonstrating significant potential for applications in encoding (Figure 29a). In contrast, Gao et al. employed a second seeded growth on preformed platelets to construct hierarchical structures.^128^ This approach relied on anchoring secondary crystal seeds onto the platelet surface through living CDSA, utilizing polymers with two distinct crystallizable blocks (Figure 29b). Due to the strong self-recognition capability of crystallization, the addition of a second crystallizable unimer led to flat-on epitaxial growth on the preformed platelets (Figure 29c). These examples highlight the ability of a “bottom-up” strategy to fabricate CDSA nanoparticles with tunable surfaces, allowing controlled hierarchical structure formation.

**Figure 29:**
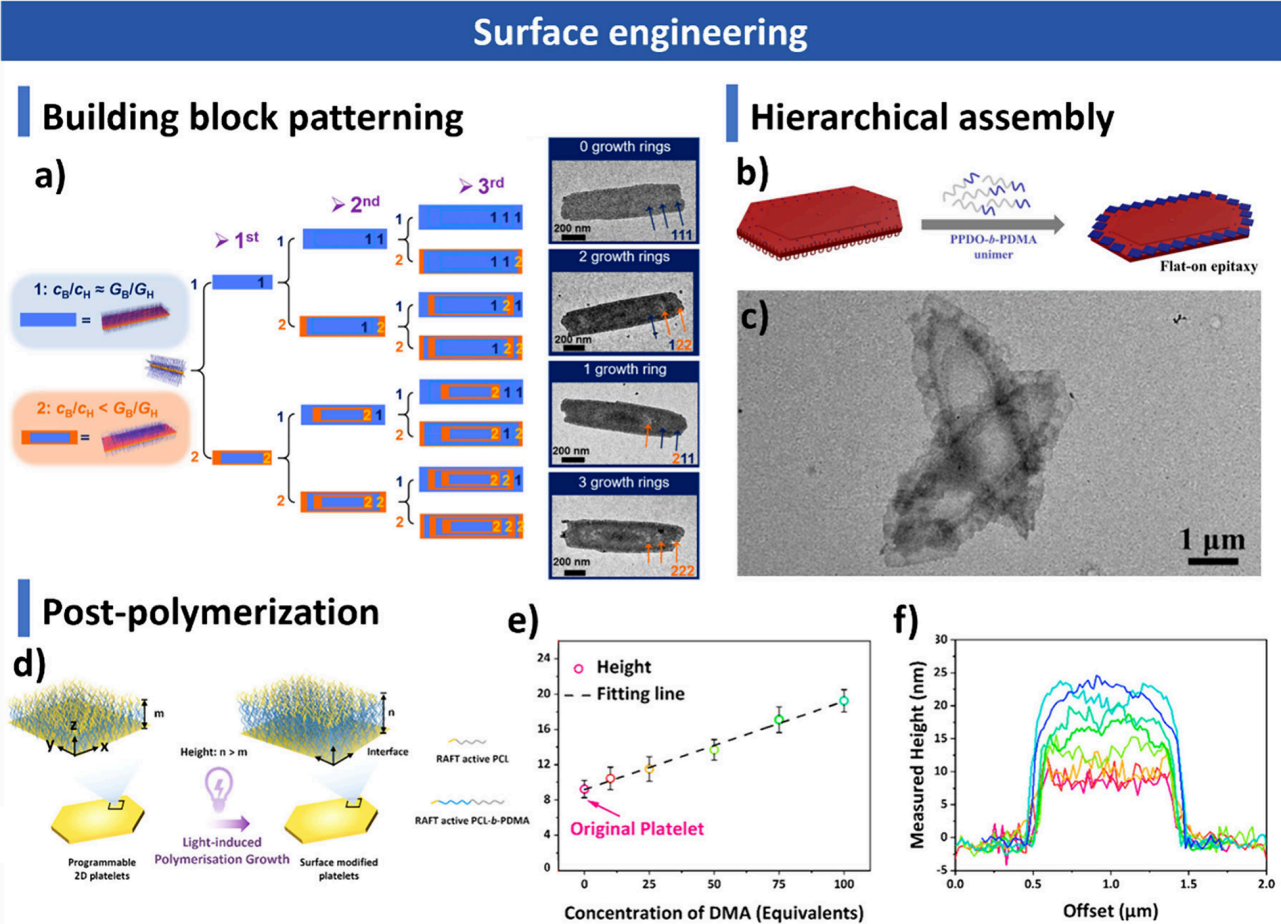
Surface Engineering of CDSA Nanoparticles. (a) Programmed design of platelets with predefined surface patterns (scale bar = 200 nm). Reproduced with permission from ref 378. Copyright 2022 American Chemical Society. (b) Schematic of flat-on epitaxial growth on preformed platelets. (c) TEM image of platelets after flat-on epitaxial growth (scale bar = 1 μm). Reproduced with permission from ref 128. Copyright 2025 American Chemical Society. (d) Surface chain extension on platelets via photopolymerization. (e) Linear correlation between platelet height and initial monomer concentration. (f) AFM height profiles of platelets after chain extension. Reproduced with permission from ref 124. Copyright 2023 American Chemical Society.

In addition, post-modification was employed to tailor the surface of CDSA nanoparticles. Notably, their crystalline core enhances stability over nanostructures assembled via amphiphilic interactions, allowing greater modification flexibility. Leveraging this, the O’Reilly and Dove groups functionalized 2D CDSA platelets in the third dimension using post-polymerization strategies.^124^ PCL-based polymers with or without RAFT groups were alternately used as unimers for seeded growth, yielding substrate platelets with selectively distributed RAFT groups, where chain extension occurred upon monomer addition during photopolymerization (Figure 29d–f). AFM analysis confirmed controlled features in the third dimension, showing a linear correlation between corona height and initial monomer concentration. Additionally, heterogeneous monomers enabled additional chain extensions, enhancing structural complexity and functionality further.

### 6.2 Stimuli-Responsive Fragmentation

Improving the stability of CDSA-derived nanoparticles during storage and under operational conditions remains a significant challenge in the field. This is particularly true for 1D cylindrical micelles composed solely of block copolymers, which are inherently fragile and prone to fragmentation. Such fragmentation can be stochastically triggered by dilution,^458^ solvent exchange,^130,207^ or biological processes such as cellular uptake.^227^ These stochastic fragmentation events can alter the morphology and size distribution of the nanoparticles, limiting their practical utility in various applications.

Although fragmentation is largely stochastic and difficult to predict, increasing attention has been directed toward achieving stimuli-responsive fragmentation as a strategy to enable controlled structural reconfiguration.^88,228,476^ This stimuli-responsive behavior allows for controlled tailoring and functionalization of nanoparticles, expanding their potential applications in drug delivery, sensing, and adaptive materials. Moreover, controlled fragmentation plays a crucial role in the fabrication of hierarchical nanostructures via CDSA, enabling the transformation of preformed assemblies into more complex architectures with tunable properties.^88^ By leveraging external triggers such as temperature,^166,476^ sonication,^228^ or chemical interactions,^38^ dynamic and reconfigurable nanomaterials can be designed, paving the way for advancements in nanotechnology and functional materials.

A collaborative study by the O’Reilly, Dove and Manners groups developed a method for preparing hollow polylactone-based platelets through selective hydrolysis of different polylactones.^38^ Initially, segmented platelets with compositionally distinct core chemistries were synthesized via seeded growth and subsequently aged in a 1 M KOH aqueous solution (Figure 30a). Over time, a gradual transformation into hollow structures was observed, with hydrolysis initiating in the P­(VL-*co*-CL) core—the block with the highest ester density—followed by degradation of the PCL region, ultimately forming PHL hollow platelets (Figure 30b). This work presents a versatile approach for selective fragmentation, leveraging polymer chemistry to control structural evolution.

**Figure 30:**
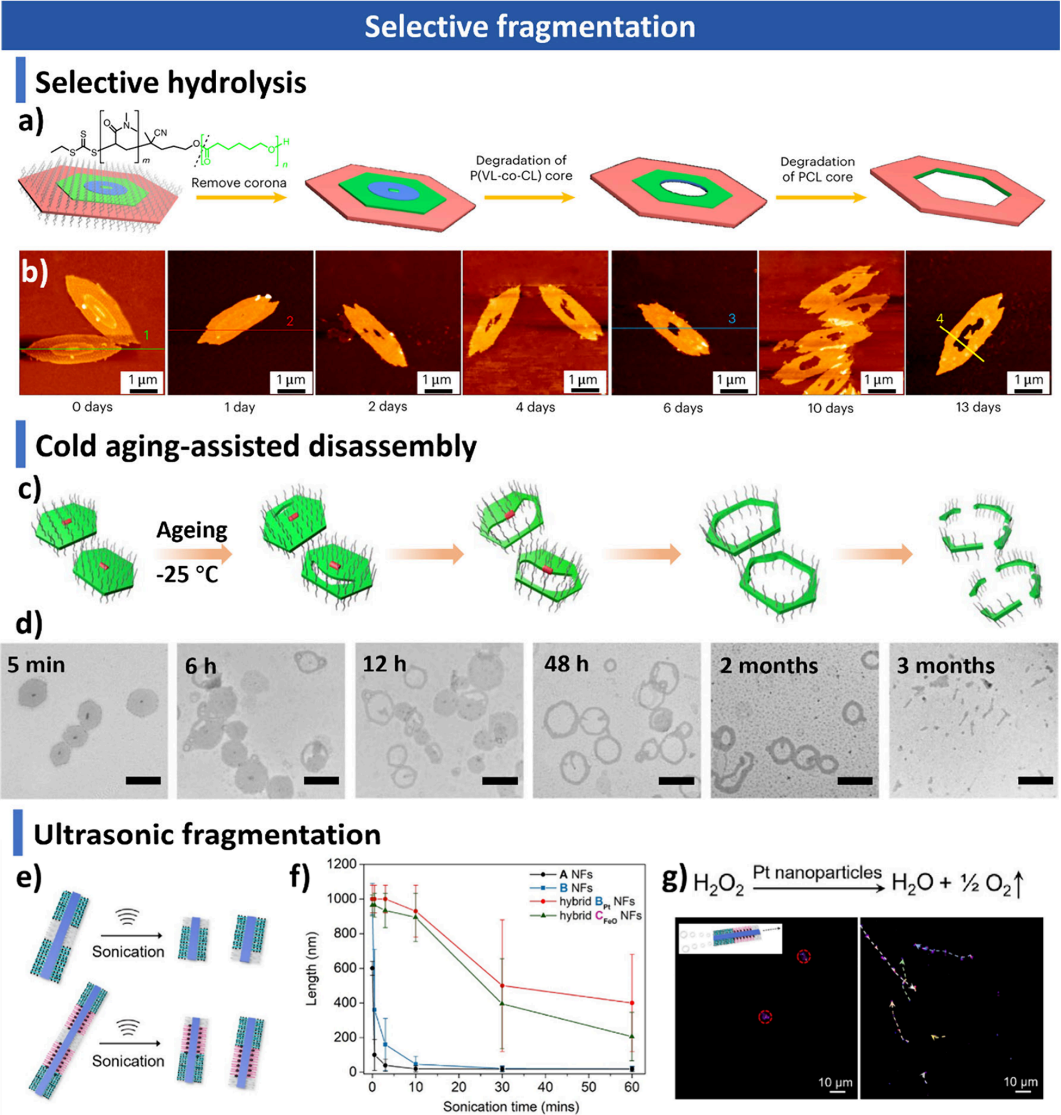
Selective fragmentation of CDSA nanoparticles. (a) Controlled degradation of polylactone platelets. (b) AFM images showing platelet degradation over time. Reproduced with permission from ref 38. Copyright 2023 Springer Nature. (c) Cold-aging assisted platelet degradation scheme. (d) TEM images showing progressive platelet degradation. Reproduced with permission from ref 476. Copyright 2024 Springer Nature. (e) Sonication-induced fragmentation of nanofibers. (f) Nanofiber length as a function of sonication time. (g) Application of fragmented nanofibers as nanomotors. Reproduced with permission from ref 228. Copyright 2024 American Chemical Society.

Alternatively, the Tong group developed a simple approach for preparing hollow platelets by aging preformed platelets at −25 °C (Figure 30c).^476^ This strategy relied on the incorporation of low-molecular-weight homopolymer during living CDSA, resulting in platelets with a less stable inner region that disassembled upon aging (Figure 30d). The disassembly rate was highly dependent on homopolymer content and chemistry, with higher homopolymer content leading to faster degradation. Notably, increasing the homopolymer content from 50 wt % to 80 wt % reduced the aging time required for hollow platelet formation from 48 h to just 30 min. Additionally, segmented platelets composed of distinct PCL, PBA, and PHA regions revealed that disassembly initiated from the PBA region, highlighting the role of polymer composition in controlling structural evolution.

Selective protection followed by removal is a well-established strategy for fabricating hierarchical CDSA nanoparticles.^34,228,344^ Recently, MacKenzie et al. developed π-conjugated nanofibers with segmented polyethylene glycol (PEG), quaternized polyfluorene (QPF), and poly­(2-vinylpyridine) (P2VP) coronas.^228^ The P2VP and QPF segments were selectively cross-linked via coordination reactions with SiO_2_-coated Fe_3_O_4_ nanoparticles and Pt nanoparticles, respectively (Figure 30e). Upon sonication, the unprotected segments with PEG coronas underwent fragmentation, resulting in noncentrosymmetric nanofibers (Figure 30f). Given that Pt nanoparticles catalyze the decomposition of hydrogen peroxide (H_2_O_2_) to generate oxygen, these asymmetric nanofibers function as nanomotors in the presence of H_2_O_2_, demonstrating potential applications in self-propelled nanoscale systems (Figure 30g).

### 6.3 Applications

#### 6.3.1 Biomedical Applications

Controlled self-assembly through CDSA enables the fabrication of uniform and highly stable nanostructures, particularly those based on biocompatible and biodegradable polylactones, polylactides, and polycarbonates.^2,5,23,477^ Control over nanoparticle dimensions and surface properties plays a pivotal role in modulating cellular interactions, circulatory behavior, and therapeutic efficacy. These features make CDSA-derived materials highly suitable for advanced drug delivery systems, imaging agents, and immune-modulating nanoplatforms, offering tunable performance for biomedical applications.^152,316^

##### 6.3.1.1 Cellular Interaction and Uptake

Understanding how CDSA-derived nanostructures interact with biological systems is essential for ensuring biocompatibility and optimizing cellular uptake.^85,227^ Parameters such as morphology, surface chemistry, and rigidity dictate endocytosis pathways, intracellular trafficking, and immune recognition, directly impacting therapeutic efficiency and safety.^165^ The internalization mechanism of CDSA nanostructures dictates their intracellular fate, are strongly influenced by nanoparticle morphology, surface chemistry, and size.^259^ In 2020, the Winnik group investigated the cellular uptake of PFS-based rod-like micelles with lengths ranging from 80 to 2000 nm in two human breast cancer cell lines (MDA-MB-436 and MDA-MB-231).^85^ Using a multicellular tumor spheroid (MCTS) model, they observed that shorter micelles (80 nm) exhibited the deepest tumor penetration. Meanwhile, the penetration depth of the micelles decreased with increasing aspect ratio. In addition to examining the impact of morphology on cellular uptake efficiency, the Chen group investigated the uptake of leaf-like PEO-*b*-PCL sheets across various cell lines, including RAW264.7 macrophages, U937 monocytes, HUVEC endothelial cells, 293T kidney cells, and HeLa cancer cells (Figure 31a,b).^117^ Their findings revealed morphology-dependent and cell-selective internalization. Macrophages exhibited the highest uptake (92% internalization in 24 h), while endothelial and monocyte uptake was moderate (53% and 29%, respectively), and minimal uptake was observed in 293T (5%) and HeLa (3%) cells.

**Figure 31:**
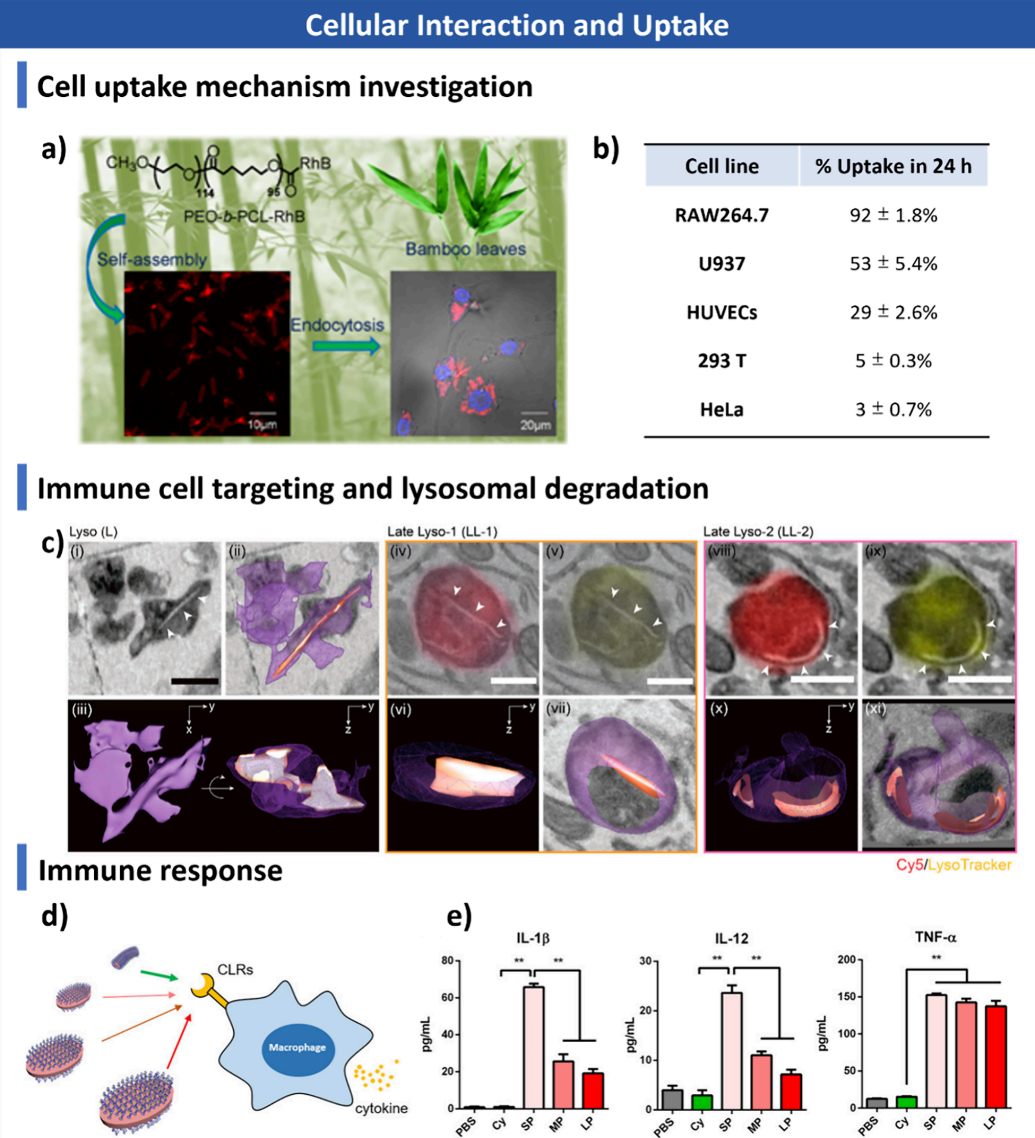
CDSA assemblies for cellular interaction and uptake. (a) Formation and interaction of leaf-like polymer sheets with cells. (b) Cellular uptake of leaf-like sheets by RAW264.7 cells. Reproduced with permission from ref 117. Copyright 2014 WILEY-VCH GmbH. (c) CLEM combined with SEM imaging was used to visualize the interaction between platelet-like particles and lysosomes at different maturation stages. Early lysosomes (magenta) and particles (ivory) were analyzed through overlay and isosurface rendering. Reproduced with permission from ref 134. Copyright 2022 American Chemical Society. (d) Interactions between glyconanoparticles and macrophages. (e) Cytokine secretion (IL-1β, IL-12, TNF-α) by macrophages after 24-h incubation with glyconanoparticles of different sizes and shapes. Reproduced with permission from ref 166. Copyright 2021 American Chemical Society.

Understanding how CDSA assemblies interact with immune cells and undergo degradation is essential. Stenzel et al. demonstrated that platelet-shaped CDSA nanostructures were preferentially internalized by macrophages, independent of size. Once internalized, these platelets were transported from early endosomes to lysosomes, undergoing degradation.^134^ Notably, even though PCL, the core-forming polymer, is typically characterized by slow degradation, complete degradation of the PCL based CDSA platelet structures was observed within 24 h following cellular uptake. This rapid degradation was attributed to a lysosome-associated pathway, as revealed by correlative light and electron microscopy (CLEM) (Figure 31c).

The immunogenicity of CDSA-derived nanostructures plays a vital role in their therapeutic applications, particularly in vaccine design and immune modulation. Li et al. investigated how the shape and size of glyco-functionalized CDSA nanoparticles influenced immune cell interactions.^166^ Their study revealed that 1D glyco-cylinders exhibited higher uptake efficiency than 2D glyco-platelets (Figure 31d,e). However, despite lower uptake, the 2D glyco-platelets across different sizes (SP, MP, LP) induced statistical higher levels of key pro-inflammatory cytokines, including IL-1β, IL-12, and TNF-α, compared to the 1D glyco-cylinders (*p* < 0.05). Besides, they demonstrated that smaller glycoplatelets triggered a more significant immune response than larger platelets, indicating that morphology plays a key role in immune activation.

##### 6.3.1.2 Biodistribution, Circulatory Behavior, and Bio Safety Evaluation

The morphology and composition of nanomaterials significantly influence their biological interactions at both the cellular and tissue levels. At the cellular level, these factors affect internalization pathways and uptake efficiency, while at the tissue level, they govern biodistribution, circulation dynamics, and metabolic fate *in vivo*. Therefore, beyond compositional differences, it is essential to investigate the *in vivo* circulation and distribution of anisotropic structures formed through CDSA to advance their biological and therapeutic applications. The fate of CDSA-based nanoparticles in circulation determines their biomedical utility. Factors such as surface charge, hydrophobicity, and aspect ratio influence circulation half-life, organ-specific accumulation, and clearance mechanisms, affecting both targeted delivery and potential off-target effects.^302,329,425^ Margination refers to the movement of nanoparticles toward the endothelial walls within blood vessels, influencing their ability to interact with target cells and tissues. The Stenzel group investigated the margination behavior of 2D platelet-like particles compared to spherical particles under controlled shear flow conditions (Figure 32a).^425^ Using fluorescence microscopy and SAXS, the study quantified the extent of margination, revealing that platelet-shaped particles exhibited higher margination than spherical particles across all shear rates (Figure 32b). The observed increased wall localization of platelet-shaped particles highlights their potential for targeted vascular drug delivery, as they may enhance endothelial interactions and site-specific therapeutic action (Figure 32c).

**Figure 32:**
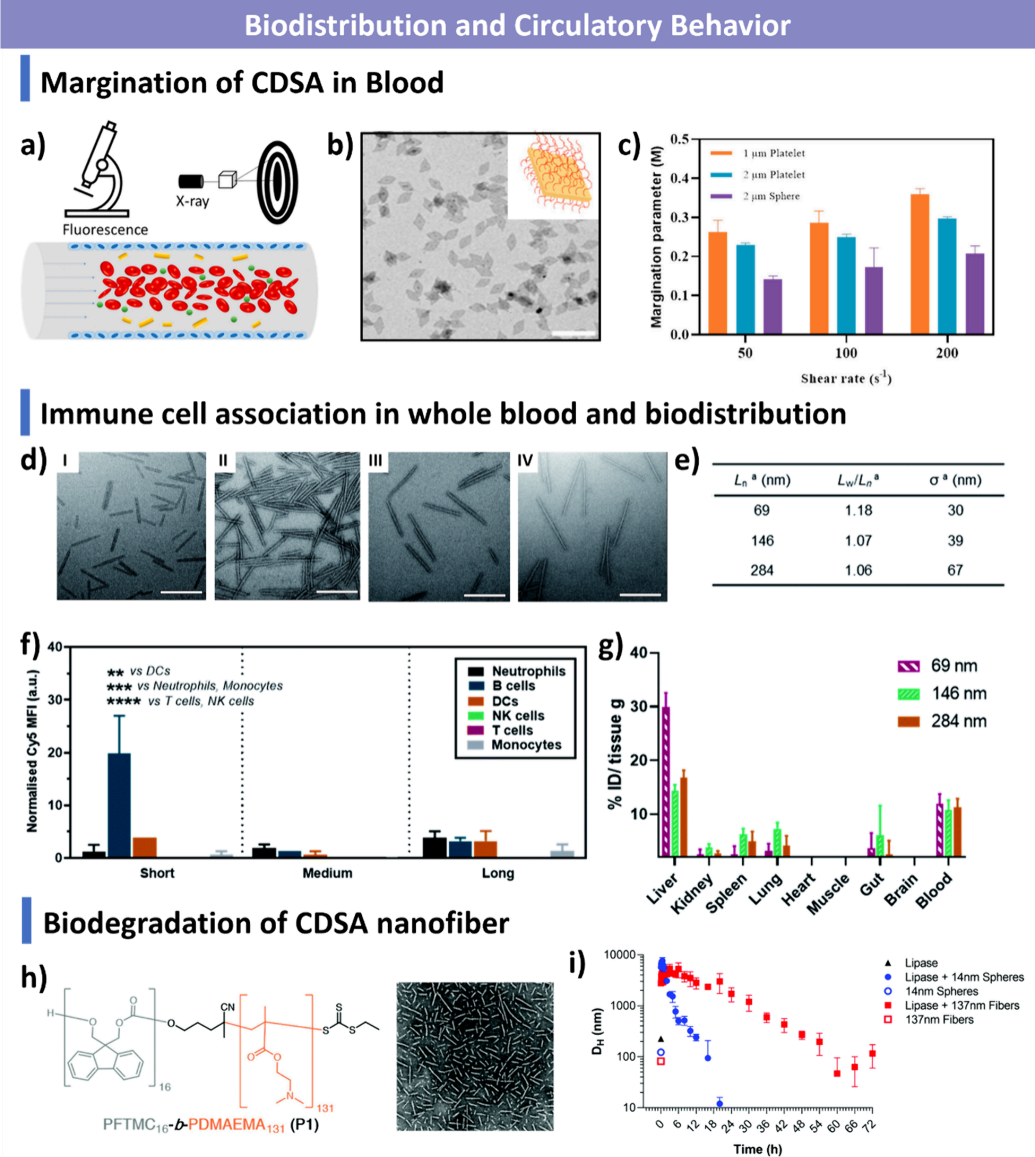
CDSA assemblies for biodistribution and circulatory behavior. (a) Schematic illustration showing the margination behavior of 2D platelet microparticles in blood flow. (b) TEM image of 2D platelets. (c) Comparison of the margination parameter for 1 μm platelets, 2 μm platelets, and 2 μm latex spheres at different apparent shear rates. Reproduced with permission from ref 425. Copyright 2023 American Chemical Society. (d) TEM images of length-controlled POx nanorods prepared by seeded growth of PMeOx_48_-*b*-PiPrOx_47_. (e) Summary of length distributions for Cy5-labeled POx nanorods used in immune cell association studies. (f) Normalized Cy5 mean fluorescence intensity showing association of POx-Cy5 nanorods with different immune cell types in whole blood after 1 h of incubation. (g) Murine biodistribution of nanorods NR1, NR2, and NR3, showing fluorescence signal measured *ex vivo* in major organs 24 h after a single injection. Reproduced with permission from ref 329. Copyright 2021 Royal Society of Chemistry. (h) Schematic illustration of the preparation of PFTMC_16_-*b*-PDMAEMA_131_ nanofibers. (i) Degradation profiles of nanofibers and nanospheres upon exposure to lipase from *Thermomyces lanuginosus*, monitored by DLS. Reproduced with permission from ref 302. Copyright 2022 Royal Society of Chemistry.

The organ distribution of nanoparticles plays a critical role in the *in vivo* performance.^478^ Kempe and co-workers evaluated the stealth properties, immune interactions, and biodistribution of poly­(2-oxazoline) (POx) nanorods, specifically designed for intravenous drug delivery (Figure 32d,e).^329^ By incorporating Cy5-labeled PiPrOx_40_ homopolymers into PMeOx_48_-*b*-PiPrOx_47_ nanorods, fluorescence tracking was performed in human blood and murine models. Flow cytometry analysis revealed that short nanorods (∼63 nm) displayed moderate association with B cells, while longer nanorods (∼216–467 nm) exhibited minimal immune interactions and low phagocytic uptake by neutrophils, monocytes, and dendritic cells, confirming their stealth characteristics (Figure 32f). Biodistribution studies in mice indicated low accumulation in the kidney, spleen, and lung, with the liver as the primary clearance organ (∼30% for short nanorods, ∼15% for longer nanorods). Over 10% of the injected dose remained in circulation after 24 h, suggesting prolonged systemic retention and making POx nanorods promising candidates for long-circulating intravenous nanomedicines (Figure 32g).

The degradation of self-assembled nanofibers in biological environments is essential for designing biodegradable drug carriers and implantable materials. Street et al. investigated the enzymatic degradation of PFTMC-based nanofibers compared to nanospheres, assessing how morphology and crystallinity influence degradation rates (Figure 32h).^302^ Using lipase from *Thermomyces lanuginosus*, DLS revealed that nanofibers degraded three times slower than nanospheres, attributed to higher core crystallinity and lower surface area accessibility. Even at physiological enzyme concentrations, nanofiber degradation followed a surface-erosion mechanism. The degradation occurred at the core–corona interface, where polymer chain folds were located (Figure 32i). These findings highlight that crystalline polycarbonates such as PFTMC offer prolonged *in vivo* stability, making them suitable for sustained drug release, theranostics, and implantable biomedical applications.

##### 6.2.1.3 Drug and Gene Delivery

CDSA-derived nanocarriers offer superior control over cargo loading, release kinetics, and stability, making them highly effective platforms for drug and gene delivery.^105,301,479^ Their modular design allows for tunable payload protection and stimuli-responsive release, improving precision and minimizing systemic toxicity in therapeutic applications.^267^ Tong et al. developed DOX-loaded 2D platelet micelles for controlled drug release.^136^ DOX, a hydrophobic anticancer drug, was encapsulated within the P4VP domains of the platelets via hydrophobic interactions. CLSM imaging confirmed DOX localization in the outer platelet regions, while drug release studies demonstrated minimal release (8%) at pH 7.4, but a significantly higher release (∼60%) at pH 5.6, mimicking the acidic tumor microenvironment (Figure 33a–c). Cellular uptake studies using 4T1 breast cancer cells confirmed efficient pH-triggered intracellular DOX release, positioning 2D platelets as promising tumor-specific nanocarriers.

**Figure 33:**
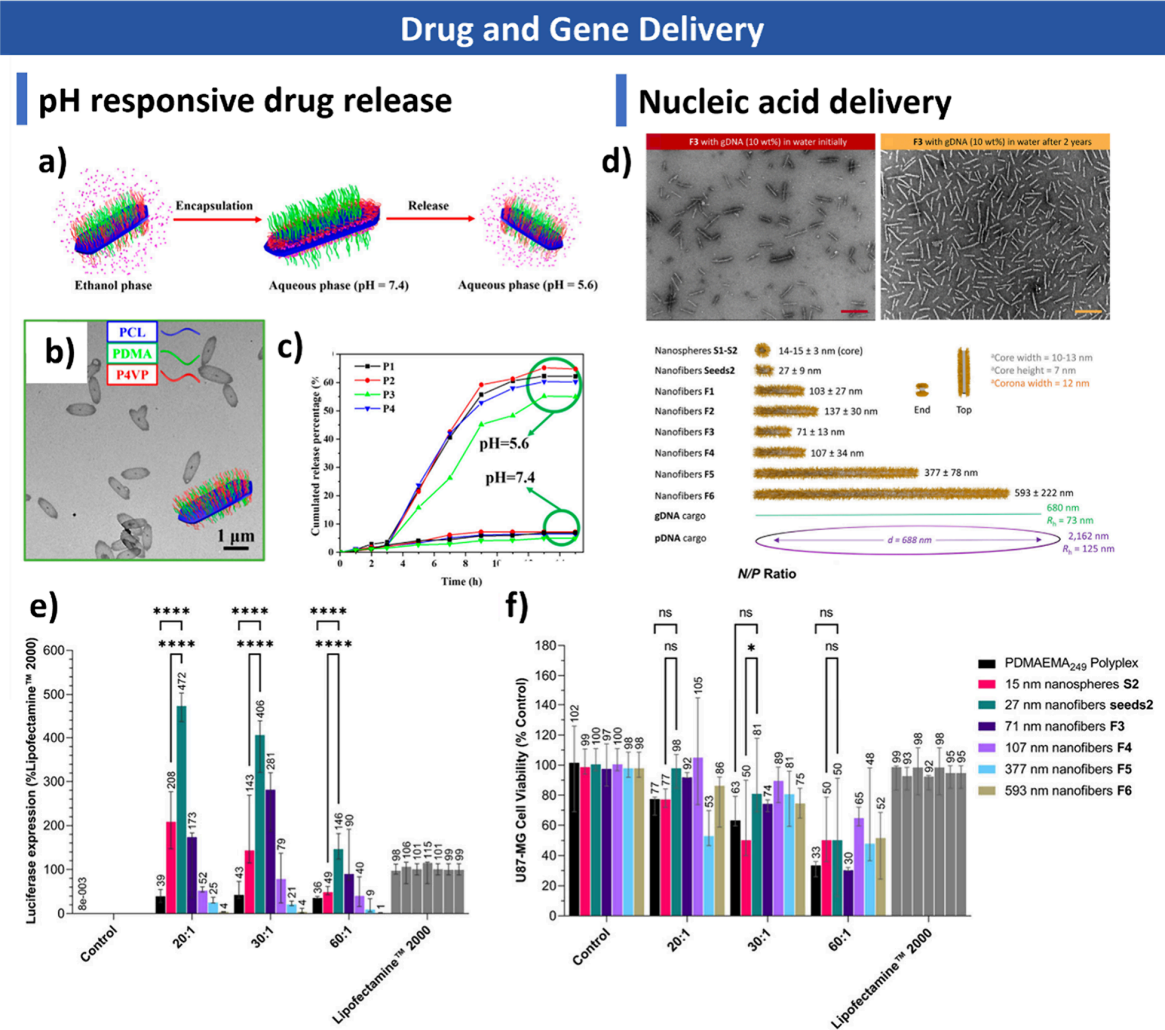
CDSA assemblies for drug and gene delivery. (a) Cargo encapsulation and release from CDSA-derived platelets. (b) TEM image of 2D platelets. (c) Cumulative release of DOX from platelets in PBS at pH 7.4 and pH 5.6. Reproduced with permission from ref 136. Copyright 2023 American Chemical Society. (d) Stability of nanofiber micelleplexes with 10 wt % gDNA over 2 years in water. (e) Transfection efficiency of luciferase plasmid in U-87 MG glioblastoma cells using different CDSA assemblies. (f) Cell viability of U-87 MG cells after transfection with luciferase plasmid using different CDSA assemblies. Reproduced with permission from ref 301. Copyright 2022 American Chemical Society.

The Manners group developed length-tunable polymer nanofibers via living CDSA for DNA delivery.^301^ These nanofiber micelleplexes effectively suppressed aggregation, tolerated high DNA loading, and exhibited superior transfection efficiency. Short nanofibers (<100 nm) achieved the highest gene transfection efficiency in U-87 MG cells, outperforming nanosphere micelleplexes, polyplexes, and commercial Lipofectamine 2000. Twenty-seven nm nanofiber micelleplexes exhibited twice the transfection activity of nanosphere micelleplexes and ten times the activity of polyplexes, while maintaining 98% cell viability (Figure 33d–f), demonstrating their potential for gene therapy applications.

#### 6.3.2 Optical Applications

One of the major challenges in optical data storage lies in achieving precise, large-scale data writing and retrieval in a controllable manner. The advancement of nanotechnology offers new strategies to address this issue.^480^ Currently, several top-down nanofabrication techniques such as photolithography and 3D printing have been employed to realize nanoscale optical storage.^481^ However, these approaches are inherently limited by their spatial resolution and precision at the micro/nanoscale, which constrains further improvements in storage density and fidelity. In contrast, bottom-up nanofabrication methods, based on the self-assembly of small molecules or polymers, offer promising alternatives for nanoscale patterning.^482^ These strategies can significantly overcome the limitations of top-down methods and enable enhanced accuracy and capacity in optical information storage. Materials such as DNA and inorganic nanoparticles have been demonstrated to form nanoscale optical patterns through self-assembly, which greatly enhances the efficiency and resolution of optical storage.^483–486^ Among these methods, CDSA stands out by harnessing crystallization forces to direct the anisotropic growth of nanostructures. This approach enables efficient and controllable one-dimensional or two-dimensional epitaxial growth, providing a robust platform for high-precision writing and reading of nanoscale optical information.^2^ The ability to engineer multicolor emission and fluorescence lifetime tuning expands the potential for next-generation optical materials.^5,17^

CDSA-based assemblies have been widely explored to create 1D fluorescent barcodes, where highly ordered nanofibers serve as optically readable encoded structures. He et al. utilized epitaxial growth to achieve successive unimer addition, generating linear rod-like micelles with multiple fluorescent segments. The resulting multiblock comicelles exhibited a banded, light-emitting barcode structure with alternating fluorescent and nonemissive segments, demonstrating high optical contrast and stability (Figure 34a,b).^74^ Most importantly, the shape and size of CDSA-derived nanoparticles can be tuned through the controlled addition of unimer solutions, following the previously described “living” growth process. The near-quantitative assembly efficiency (∼100%) ensures excellent reproducibility of information encoding.

**Figure 34:**
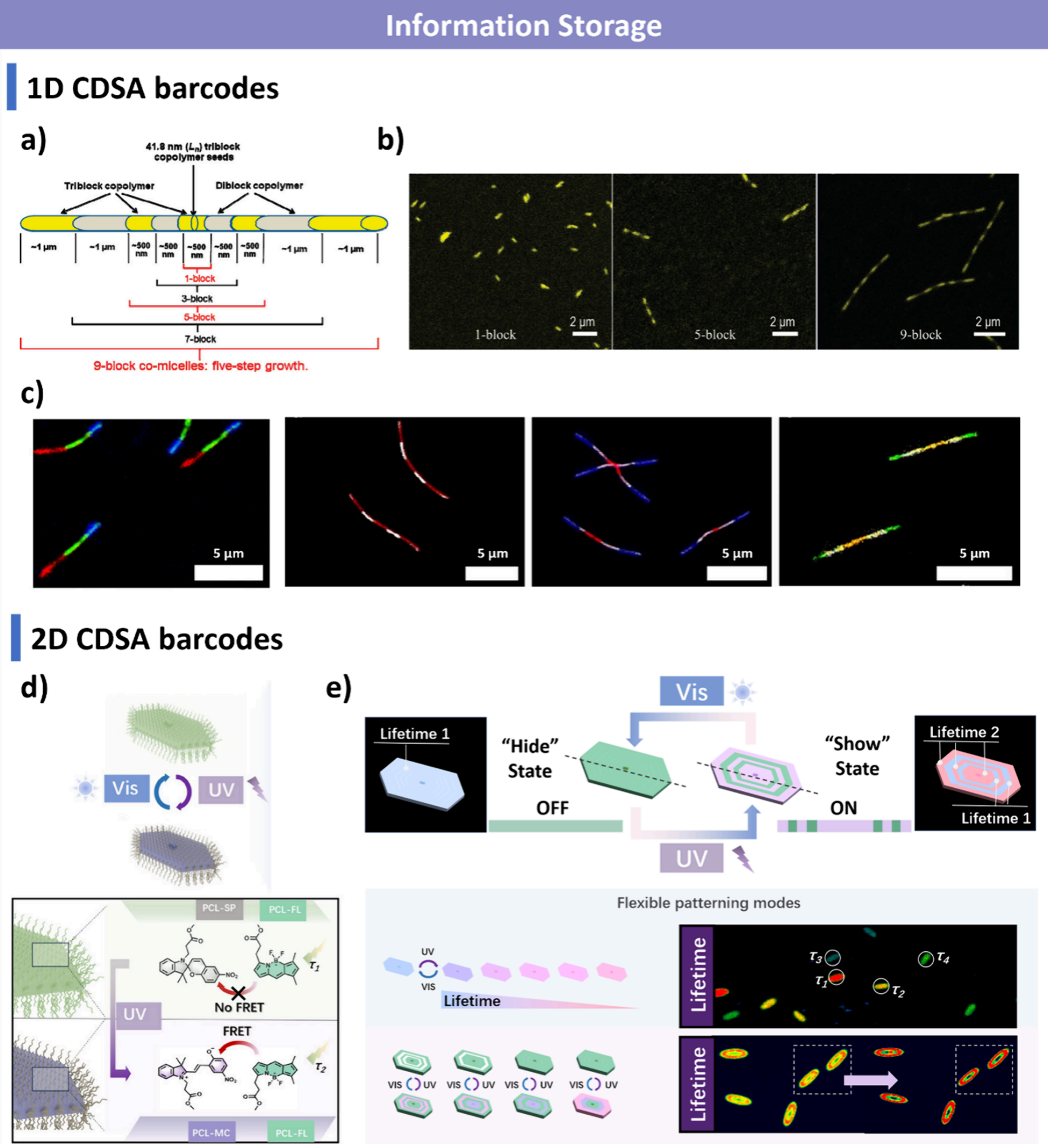
CDSA assemblies for optical applications. (a) Formation of 9-block comicelles with alternating PFS_30_-*b*-P2VP_300_ diblock and PFS_30_-*b*-P2VP_300_-*b*-PDEHPV_13_ triblock segments. (b) Confocal images of 1-block, 5-block, and 9-block comicelles. Reproduced with permission from ref 74. Copyright 2011 American Chemical Society. (c) Confocal images of 7-block RGB nanopixels and pentablock micelles with encoded color patterns. Reproduced with permission from ref 89. Copyright 2014 Springer Nature. (d) Photoresponsive behavior of 2D platelets via reversible SP–MC transformation under UV–vis irradiation. (e) Preparation and fluorescence imaging of 2D platelets, demonstrating dynamic fluorescent lifetime patterning upon photoirradiation. Reproduced with permission from ref 487. Copyright 2025 Wiley-VCH GmbH.

To mimic RGB color systems, Hudson et al. used a series of fluorescent block copolymers and the living CDSA approach to create tunable nanoscale pixels. Monodisperse cylindrical multiblock nanostructures were synthesized with distinct fluorescent domains, each maintaining persistent and tunable color emission (Figure 34c). These RGB nanopixels, assembled via sequential unimer addition, demonstrate the feasibility of automated, high-throughput nanostructure synthesis for complex optical encoding.^89^

Building upon 1D architectures, 2D barcode systems have been developed using CDSA to further enhance optical storage capacity. Xie et al. introduced a living seeded-growth CDSA method to fabricate stable hierarchical 2D microbarcodes with ultrahigh information density in both levels of colors and layers. By programming the sequential addition of polymer layers, distinct fluorescence patterns and spatially controlled geometric information were encoded within each platelet, significantly expanding the capabilities of optical storage.^114^ Furthermore, Tong, Xie, and co-workers developed “Light-Flashable” 2D polymeric fluorescent lifetime microbarcodes using living CDSA seeded growth, enabling controlled multidimensional nanoscale spatial encoding.^487^ By integrating photoswitchable spiropyrans (SP), they achieved dynamic fluorescence lifetime control through energy transfer modulation (Figure 34d). This approach enhances data storage robustness by enabling light-triggered signal output and multidimensional encoding. Such advanced microbarcodes hold promise for high-density data storage by leveraging programmable fluorescence lifetimes and responsive light patterns (Figure 34e).

#### 6.3.3 Energy and Catalysis Applications

The controlled morphological features afforded by CDSA has been leveraged to optimize nanostructures for energy conversion and catalysis,^7,488^ particularly in conjugated polymer systems.^50^ Crystalline order, defined interfaces, and tunable electronic properties enable enhanced charge transport, light harvesting, and catalytic efficiency, making CDSA-based materials highly relevant for photovoltaics, optoelectronics, and chemical transformations.^5^

##### 6.3.3.1 Energy Transfer

Nanostructures generated via CDSA provide well-defined pathways for exciton migration and charge separation, critical for light-harvesting and energy conversion applications. Controlled domain interfaces and hierarchical assembly further enhance energy transfer efficiency.^5,229,456^

Das et al. demonstrated the fabrication of 2D PLLA-based assemblies through CDSA, utilizing PLLA chains end-functionalized with dipolar dyes such as naphthalene monoamide (NMI) or hydrophobic pyrene (PY).^454^ In isopropanol, PLLA chains crystallized into diamond-shaped platelets, where terminal dyes were arranged in a 2D array via dipolar interactions or π-stacking, resulting in tunable emission properties. The dipolar dyes enhanced colloidal stability and structural uniformity, although this effect was less pronounced in PY-functionalized systems. When two distinct π-scaffold-functionalized PLLA homopolymers cocrystallized, the resulting 2D assemblies exhibited Förster resonance energy transfer (FRET), forming coplatelets with ∼80% energy transfer efficiency over short distances (∼10 nm) (Figure 35a,b).

**Figure 35:**
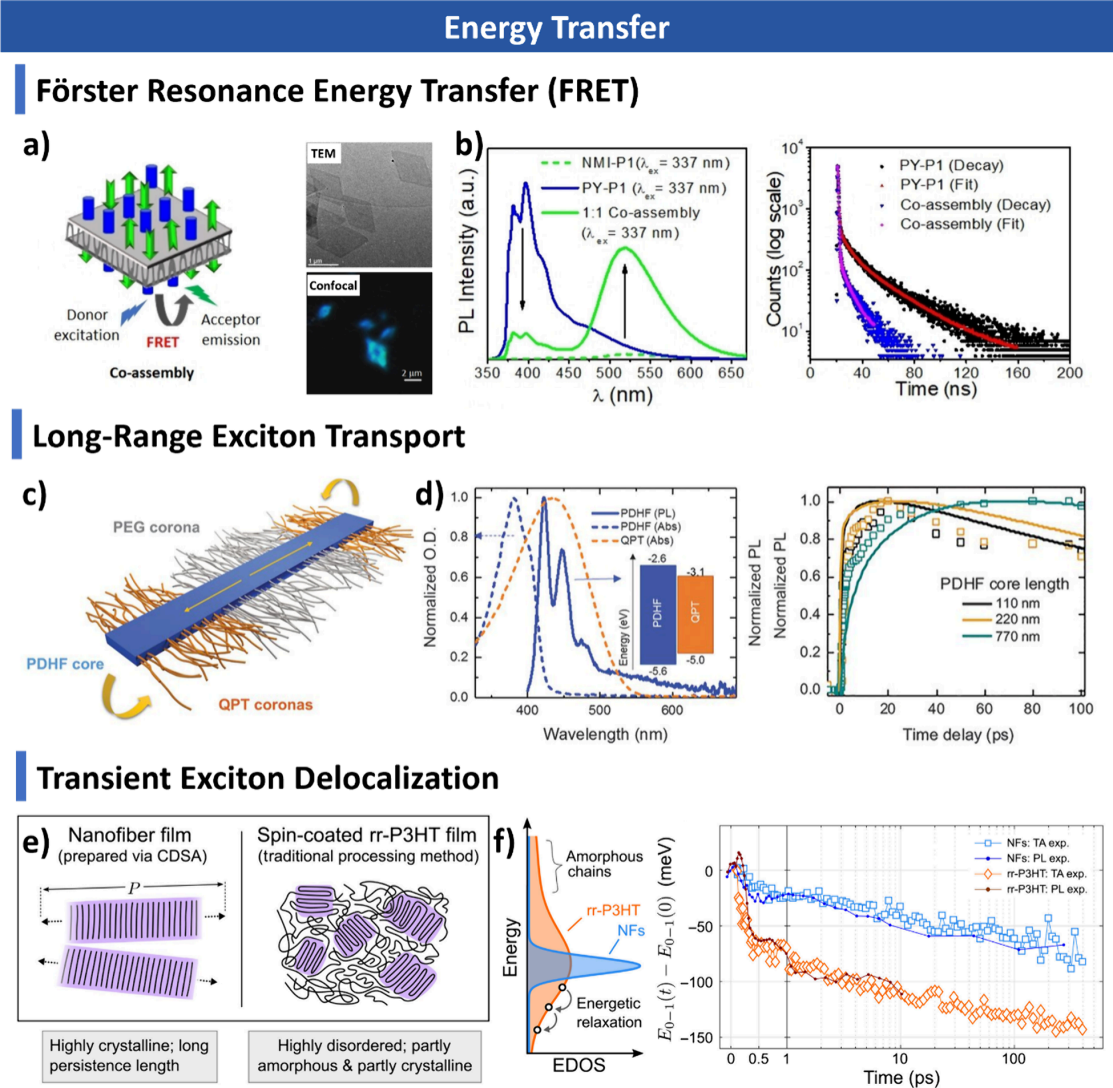
CDSA assemblies for energy transfer. (a) TEM and confocal images of diamond-shaped platelets formed by coassembly of NMI-P1 and PY-P1 (1:1) in iPrOH. (b) Emission spectra and time-resolved fluorescence decay profiles of PY-P1, NMI-P1, and their coassembly. Reproduced with permission from ref 454. Copyright 2022 Wiley-VCH GmbH. (c) Schematic of segmented B-A-B nanofibers with donor and acceptor domains. (d) Absorption and photoluminescence spectra of QPT homopolymer, unsegmented PDHF nanofibers, and photoluminescence kinetics of the QPT signal rise in segmented PDHF B-A-B nanofibers. Reproduced with permission from ref 40. Copyright 2018 American Association for the Advancement of Science. (e) Comparison of nanofiber film microstructure with spin-coated rr-P3HT films. (f) Electronic density of states (EDOS) schematic showing tighter EDOS and lower Urbach energy in nanofibers compared to spin-coated rr-P3HT films. Reproduced with permission from ref 198. Copyright 2021 American Association for the Advancement of Science.

While FRET enables efficient energy transfer over nanometer-scale distances, exciton transport in conjugated polymers is often limited by incoherent hopping. Extending exciton diffusion to longer distances (hundreds of nanometers) requires controlled nanostructure size, morphology, and crystalline order. Manners, Friend, and co-workers synthesized organic semiconducting nanofibers featuring a crystalline poly­(di-*n*-hexylfluorene) (PDHF) core, polyethylene glycol corona, and polythiophene end blocks.^40^ These nanofibers exhibited efficient exciton transfer from the PDHF core to the lower-energy polythiophene end blocks along the interchain π–π stacking direction, achieving exciton diffusion lengths exceeding 200 nm and a diffusion coefficient of 0.5 cm^2^ s^–1^ (Figure 35c,d). In this study, WAXS measurements confirmed pronounced π–π stacking and high crystalline order within the PDHF core, which was directly correlated with the enhanced exciton diffusion, as excitons preferentially migrated within these ordered domains. The combination of structural characterization and photophysical measurements thus provided clear experimental evidence linking improved structural order to enhanced exciton transport in these CDSA-derived nanofibers.

Further advancing this concept, Rao et al. synthesized highly ordered poly­(3-hexylthiophene) (P3HT) nanofiber films via living CDSA, demonstrating a distinct regime of energy transport characterized by transient exciton delocalization.^198^ Through energy exchange with vibrational modes, excitons transiently accessed spatially extended states, yielding an exciton diffusion coefficient of 1.1 ± 0.1 cm^2^ s^–1^ and diffusion lengths of 300 ± 50 nm. Energetic disorder, which typically limits exciton transport, was significantly reduced in these nanofibers. This was evidenced by a 40-fold lower baseline absorption at low energies (<1.7 eV) compared to regioregular P3HT (Figure 35e). Structural characterization using PXRD confirmed crystalline packing and π–π stacking within the nanofibers, while low-dose scanning electron diffraction (SED) directly visualized long-range orientational order, with an average chain alignment persistence length of ∼80 nm (Figure 35f). The living CDSA growth mechanism played a crucial role in this enhancement by promoting slow epitaxial growth from seed micelles, selectively incorporating defect-free polymer chains, suppressing structural distortions, and yielding highly ordered crystalline nanostructures superior to those obtained via conventional spin-coating methods.

##### 6.3.3.2 Optoelectronics

CDSA-based assemblies with well-defined conjugated domains and molecular ordering exhibit tailored optoelectronic properties, supporting applications in organic photovoltaics, light-emitting diodes (LEDs), and field-effect transistors (FETs).^318,489^ Their ability to facilitate charge mobility and control exciton recombination dynamics positions them as promising candidates for next-generation electronic devices.^217,454,490,491^

Li et al. reported the solution-processable fabrication of low-dispersity electroactive fiber-like micelles with controlled lengths using regioregular poly­(3-hexylthiophene) (rr-P3HT)-based π-conjugated diblock copolymers.^447^ Tunneling atomic force microscopy (T-AFM) confirmed that individual fibers exhibited high conductivity, significantly surpassing that of the substrate (Figure 36a,b). When incorporated as the active layer in FETs, charge carrier mobility was strongly dependent on the degree of polymerization of the core-forming block and fiber length, but independent of corona composition. By aligning unfragmented rrP3HT_106_-*b*-rsP3HT_47_ fibers via dip-coating, unoptimized mobility values exceeding 1.5 ± 0.16 × 10^–2^ cm^2^ V^–1^ s^–1^ were achieved.

**Figure 36:**
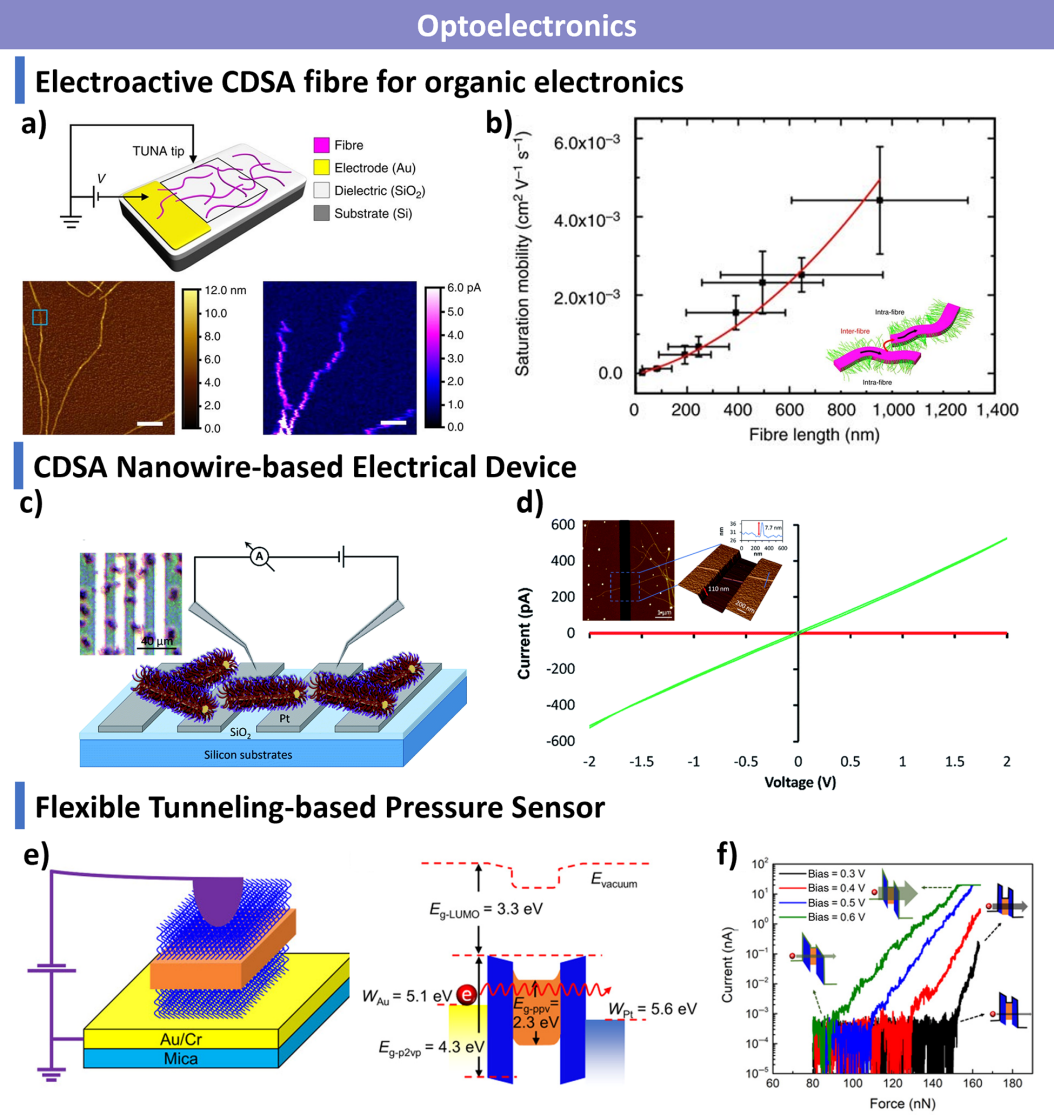
CDSA Assemblies for Optoelectronics. (a) Tunneling atomic force microscopy (TUNA) image of fibers. (b) Field-effect mobility of rrP3HT_106_-*b*-rsP3HT_47_ fiber-based devices. Reproduced with permission from ref 447. Copyright 2017 Springer Nature. (c) Fiber-like micelles bridging the interelectrode gap of an electrical device. (d) Schematic of a mesoscopic electrical circuit formed from fiber-like terpolymer micelles, with *I*–*V* curves before (red) and after (green) NOBF_4_ doping. Reproduced with permission from ref 489. Copyright 2020 Royal Society of Chemistry. (e) Schematic of the C-AFM measurement setup and band alignment in the Probe/PP-PPV_7_-*b*-P2VP_29_/Au tunneling junction. (f) Current–force curves at different biases, with insets showing tunneling barriers under applied voltages. Reproduced with permission from ref 217. Copyright 2020 Chinese Chemical Society.

Houlton and co-workers leveraged CDSA principles to develop an method for constructing electrical circuits using redox-active fiber-like block terpolymer micelles.^489^ These micelles, derived from the Gaussian scission of block terpolymer seeds, were designed to chemisorb onto electrode surfaces, enabling controlled interfacial micelle growth with defined lengths. The fiber-like micelles successfully bridged a 10 μm interelectrode gap and were chemically oxidized to form conductive “wires.″ Electrical measurements on micelle bundles grown on patterned platinum microband electrodes showed a measurable current after doping with nitrosonium tetrafluoroborate. Current–voltage curves post-doping confirmed a substantial increase in conduction, consistent with oxidative doping of the polythiophene core (Figure 36c,d).

The He group demonstrated the formation of uniform, controllable 2D rhombic micelles using poly­(p-phenylenevinylene) (PPV)-based BCPs.^217^ These 2D nanostructures, driven by π–π interactions, enable controlled separation of the semiconducting and insulating components within the BCPs. These nanostructures, driven by π–π interactions, enabled controlled separation of semiconducting and insulating components within the BCPs. Utilizing these rhombic micelles, a vertical tunneling device was fabricated, achieving an on–off current ratio exceeding 10^4^ and a high on-state current density of 6000 A cm^–2^ under conductive AFM. Furthermore, the group developed a tunneling-based pressure sensor, leveraging the compressibility of poly­(2-vinylpyridine) (P2VP) chains in the insulating layer. External pressure modulated the vertical tunneling behavior by altering the insulating layer thickness, resulting in a reversible and stable tunneling current that reached 3 nA under a maximal force of 148 nN. The device exhibited excellent repeatability and durability, with minimal degradation over repeated measurements (Figure 36e,f).

##### 6.3.3.3 Catalysis

The well-ordered architecture of CDSA-derived nanostructures enables controlled spatial distribution of active sites, enhancing catalytic selectivity and efficiency.^492,493^ These assemblies serve as both standalone catalysts and structured supports, optimizing reactant accessibility, stabilizing intermediates, and modulating catalytic pathways for improved performance in chemical transformations.^449,494^

Certain block copolymer assemblies formed through CDSA exhibit inherent catalytic activity, where the polymer itself functions as the catalyst. In these cases, catalytic functionality is typically localized in the corona of the micelles, directly participating in the reaction. For example, Tian et al. developed a high-performance, recyclable photocatalytic core–shell nanofiber system integrating a cobalt catalyst and a BODIPY photosensitizer for visible-light-driven hydrogen production.^449^ Utilizing living CDSA, they achieved controlled nanofiber composition, microstructure, and dimensions, enabling fine-tuning of fiber length, spatial functionality distribution, and colloidal stability—key factors in enhancing catalytic performance. Under optimized conditions, the CDSA-derived nanofibers exhibited a quantum yield of ∼4.0% for solar-to-hydrogen conversion, a turnover number exceeding 7000 over 5 h, and a turnover frequency of over 1400 h^–1^. Notably, with just 1.34 μg of catalytic polymer, the system achieved a hydrogen production rate of 0.327 μmol h^–1^, corresponding to an exceptionally high catalytic efficiency of 244300 μmol h^–1^ g^–1^ (Figure 37a,b). These findings underscore the potential of CDSA for designing efficient and tunable photocatalytic systems.

**Figure 37:**
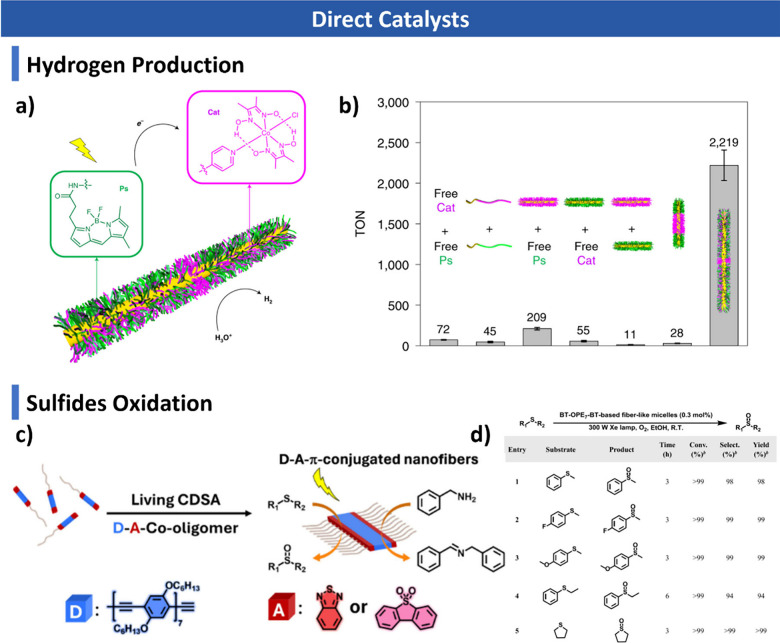
CDSA Assemblies as Direct Catalysts. (a) Schematic illustration of Cat/Ps blend nanofibers for HER, featuring a crystallizable PFS core with corona segments bearing catalysts and photosensitizers. (b) HER performance of Cat/Ps nanofibers after 5 h of irradiation in MeOH, compared to control samples. Reproduced with permission from ref 449. Copyright 2020 Springer Nature. (c) Donor–acceptor nanofibers with tunable length and composition for enhanced photocatalysis. (d) Photocatalytic selective oxidation of sulfides to sulfoxides using BT-OPE_7_-BT-*b*-PNIPAM_36_ fiber-like micelles. Reproduced with permission from ref 209. Copyright 2024 American Chemical Society.

Feng et al. synthesized donor–acceptor (D–A) co-oligomer nanofibers using CDSA.^209^ These oligomers incorporated electron-deficient benzothiadiazole (BT) or dibenzo­[b,d]­thiophene 5,5-dioxide (FSO) units at the termini of an oligo­(p-phenylene ethynylene) heptamer core [BT-OPE_7_-BT, FSO-OPE_7_-FSO], enhancing π–π stacking interactions in an A–D–A π-conjugated structure. In contrast, D–A–D configurations with a single BT unit in the middle disrupted packing efficiency, highlighting the crucial role of terminal electron-deficient groups in forming ordered assemblies. To expand their applicability, terminal alkynes were used to synthesize diblock copolymers, which self-assembled via CDSA in ethanol into crystalline π-conjugated nanofibers (CPNFs). The CDSA process enabled controlled nanofiber length via self-seeding, seeded growth, and block comicelle formation. The A–D–A nanofiber architecture facilitated efficient charge separation at interfaces with distinct band gaps, promoting photogenerated exciton dissociation and subsequent electron–hole transfer for redox reactions (Figure 37c,d). CPNFs with a BT-OPE_7_-BT core exhibited superior photocatalytic activity, catalyzing sulfide oxidation to sulfoxide and benzylamine oxidation to N-benzylidenebenzylamine. Compared to OPE_9_-core nanofibers, BT-OPE_7_-BT-based structures demonstrated enhanced charge generation, separation, and transfer due to their higher crystalline order and optimized electronic properties.

CDSA-derived structures, such as platelets and micelles, can serve as scaffolds for stabilizing catalytic species, including metal nanoparticles.^495–498^ While the catalytic activity originates from these metal nanoparticles, the polymer assemblies provide a structured, tunable environment that enhances stability and efficiency.^230,488,492^

Qiu et al. successfully fabricated multifunctional micellar brushes on silicon surfaces via crystallization-driven growth.^79^ Using PFS-*b*-P2VP diblock copolymer seeds, they immobilized micellar precursors onto silicon wafers through robust hydrogen bonding between the substrate’s silanol groups and the P2VP corona. Subsequent addition of unimers enabled controlled fiber-like micellar brush growth up to ∼1 mm in length. The brush density, length, and coronal composition were tuned during assembly, allowing for postgrowth functionalization. By leveraging coordination interactions, the micellar brushes were selectively decorated with ∼15 nm gold nanoparticles (AuNPs), yielding functional hybrid surfaces. These AuNP-decorated brushes exhibited high catalytic efficiency in the borohydride-mediated reduction of 4-nitrophenol to 4-aminophenol (Figure 38a,b). The hybrid surfaces demonstrated exceptional durability and recyclability, maintaining consistent catalytic performance over 15 cycles.

**Figure 38:**
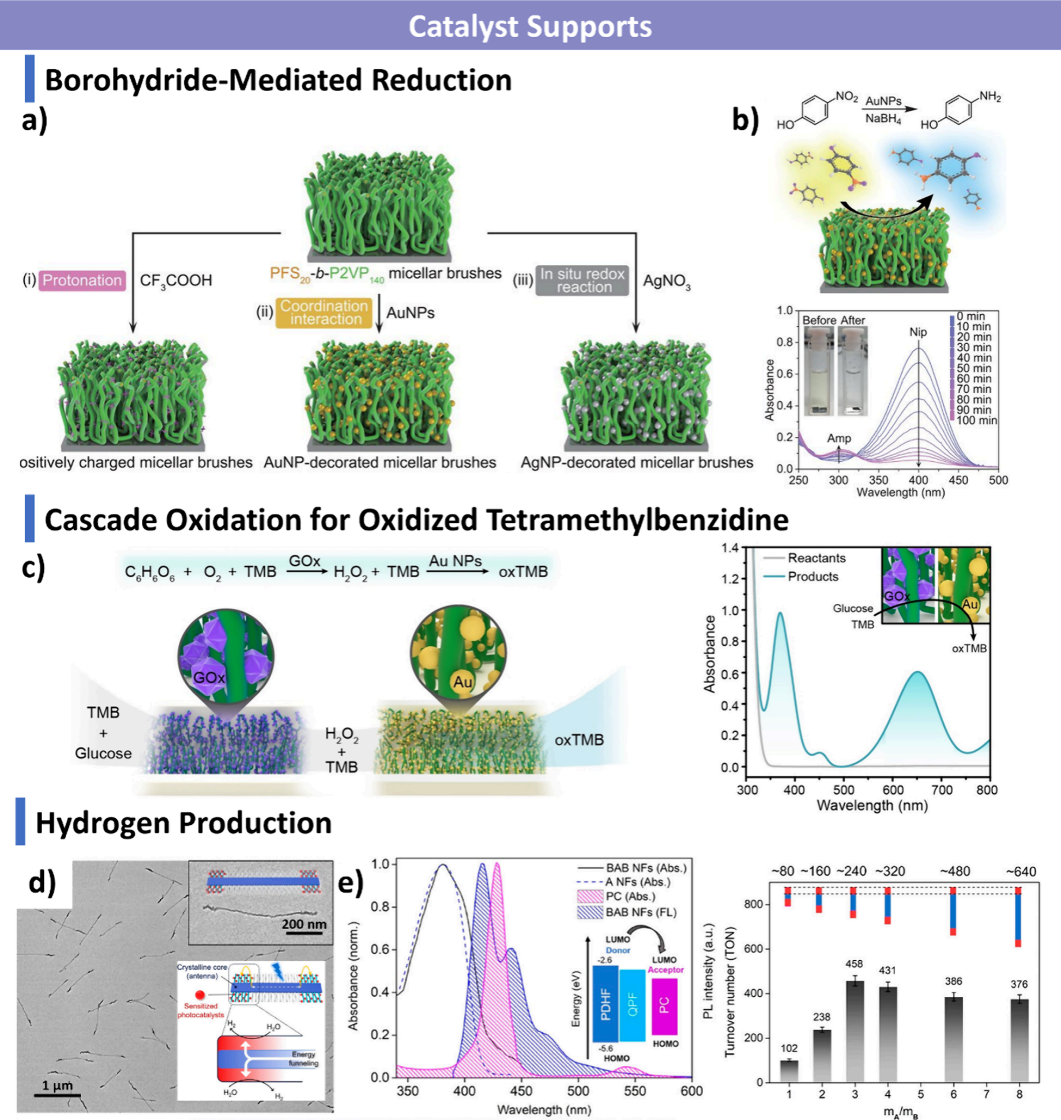
CDSA Assemblies as Catalyst Supports. (a) Functionalization pathways of PFS_20_-*b*-P2VP_140_ micellar brushes via protonation, coordination, and redox chemistry. (b) 4-Nitrophenol reduction catalyzed by AuNP-decorated micellar brushes, with UV–vis monitoring. Reproduced with permission from ref 79. Copyright 2019 American Association for the Advancement of Science. (c) Cascade reaction in PBS catalyzed by GOx- and AuNP-decorated micellar brushes, monitored by UV–vis. Reproduced with permission from ref 493. Copyright 2021 Wiley-VCH GmbH. (d) TEM images of HER nanofibers with 5% photocatalyst loading, and UV–vis spectra of B-A-B nanofibers, A nanofibers, and Co­(II) porphyrin in H_2_O/MeOH (1:1). (e) Fluorescence spectra and turnover numbers (TONs) of HER nanofibers with varying PDHF_14_-*b*-PEG_227_ to PDHF_15_-*b*-QPF_16_ ratios after 8 h of irradiation. Reproduced with permission from ref 231. Copyright 2023 American Chemical Society.

Micellar brushes have also been integrated into flow reactor systems, enabling complex reaction sequences with enhanced efficiency and sustainability. Qiu et al. developed dense cylindrical micelle brush supports within glass capillary flow reactors using a living CDSA process initiated by preimmobilized micelle seeds.^493^ The active corona of these brushes provided a versatile platform for decorating various nanocatalysts through direct capture or *in situ* growth. These micelle-based flow reactors exhibited high catalytic efficiency across diverse reactions, including organic reductions, Suzuki coupling, and photolytic degradation, while maintaining recyclability over multiple cycles. A key advantage of this system was its capability to facilitate multistep cascade reactions in continuous flow. As a demonstration, a sequential reaction was designed, wherein glucose oxidation catalyzed by glucose oxidase (GOx) generated H_2_O_2_, which subsequently oxidized 3,3′,5,5′-tetramethylbenzidine (TMB) via AuNPs with peroxidase-like activity (Figure 38c). This work underscores the potential of micellar brushes as efficient catalytic supports in advanced flow reactors, offering precision, adaptability, and sustainability for complex chemical transformations.

Zhang et al. developed recyclable, high-performance photosynthetic nanofibers featuring a crystalline π-conjugated polyfluorene core as an antenna system.^231^ This system efficiently funneled absorbed solar energy to spatially defined Co­(II) porphyrin photocatalysts, enabling hydrogen evolution reactions (HERs). By tuning nanofiber dimensions, they leveraged extended exciton diffusion lengths (>200 nm) associated with the highly crystalline polyfluorene core, achieved via a living CDSA-seeded growth method. This approach facilitated efficient solar-driven hydrogen production, yielding a turnover number (TON) exceeding 450 after 8 h of irradiation, a hydrogen production rate of ∼65 mmol h^–1^ g^–1^, and an overall quantum yield of 0.4% within the <405 nm wavelength range—beyond the absorption limit of the molecular photocatalyst. The study also highlighted the role of exciton dynamics in enhancing catalytic efficiency. A reduced fluorescence lifetime for the PC segments in HER nanofibers indicated accelerated exciton quenching in the crystalline PDHF core upon Co­(II) porphyrin incorporation. Shortening the terminal PC segment length from ∼500 nm to ∼200 nm further improved exciton transfer efficiency, as evidenced by a shorter exciton lifetime (96.5 ps) and a higher TON of 427 (Figure 38d,e).

By leveraging the precise control enabled by CDSA, highly ordered nanostructures have been developed to optimize energy transport, charge transfer, and catalytic activity. These structures exhibit exceptional tunability in morphology, size, and composition, driving advancements in solar energy harvesting, hydrogen production, organic electronics, and multistep cascade catalysis. The interplay between structural order and functional performance underscores the transformative potential of CDSA in designing tailored materials for diverse energy related applications.

#### 6.3.4 Functional Additives

Use as additives is another important application of self-assembled nanoparticles. Nanoparticles prepared by CDSA were used to improve the mechanical properties of bulk materials. Recently, Li and co-workers toughened commercial PMMA resin using poly­(cholesteryl methacryloyloxy ethyl carbonate) (PCholMA)-based cylinders, and the influence of cylinder length on toughening effect was studied. Results indicated that the optimal length of cylinders that can achieve the maximum toughening effect is 947 nm, which can bridge cracks and increase chain flexibility of the matrix (Figure 39a–c). In addition, 1D cylinders were used to enhance the mechanical strength of hydrogel.^113^ Besides cylinders, 2D platelets have been used to modify hydrogel as well, with the shape of the nanoparticles shown to play a key role in tuning the strength of adhesion and self-healing properties.^499^

**Figure 39:**
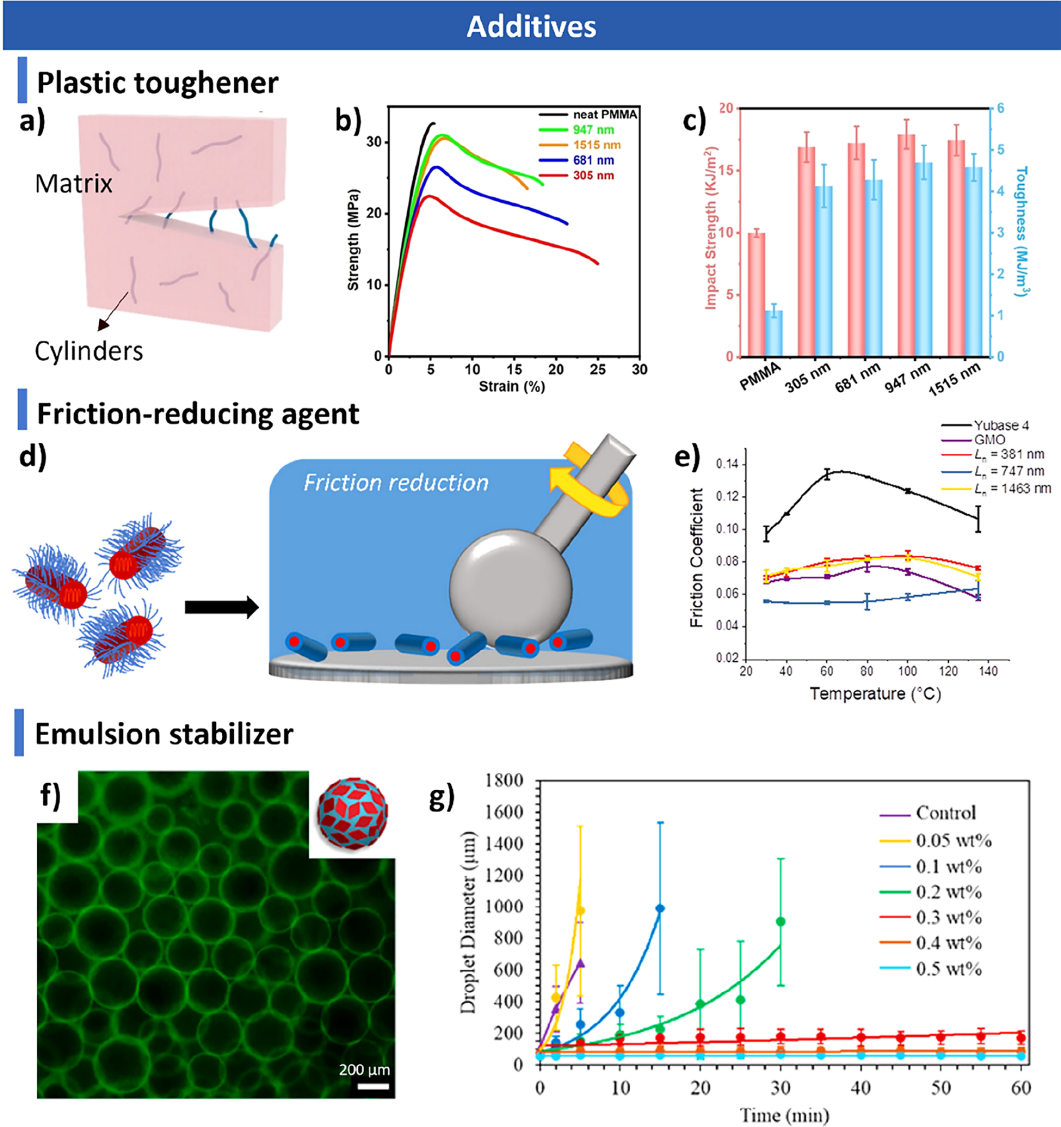
CDSA nanoparticles used as additives. (a) Toughening mechanism of cylinders in PMMA. (b) Stress–strain curves of PMMA loaded with cylinders of different lengths. (c) Comparison of impact strength and toughness of PMMA with various cylinder lengths. Reproduced with permission from ref 245. Copyright 2023 Wiley-VCH GmbH. (d) Friction-reducing mechanism of cylinders in lubricants. (e) Friction coefficient analysis comparing different cylinder lengths in Yubase 4, neat base oil, and glycerol monooleate (GMO). Reproduced with permission from ref 39. Copyright 2023 American Chemical Society. (f) Fluorescence microscopy image of emulsion droplets stabilized by platelets. (g) Emulsion droplet diameter over time at different platelet concentrations. Reproduced with permission from ref 142. Copyright 2017 American Chemical Society.

CDSA nanoparticles have emerged as promising oil friction modifiers in the automotive industry, overcoming the sedimentation issues of conventional inorganic additives caused by their high density relative to oil. Clamor et al. addressed this problem by synthesizing cylindrical nanoparticles in oil with controlled length using living CDSA, which ensured better compatibility with oil and minimizing sedimentation (Figure 39d,e). The ability to tune the cylinder length allowed for a comprehensive structure–activity relationship study, which demonstrated that all synthesized samples effectively reduced the friction coefficient of oil. Notably, the most significant reduction was achieved with a cylinder length of 747 nm, outperforming the widely used commercial additive glycerol monooleate. These findings underscore the potential of CDSA nanomaterials in developing advanced lubrication systems, opening new avenues for optimizing energy efficiency and minimizing wear in automotive applications.

2D particles demonstrate superior performance in emulsion preparation compared to traditional amphiphilic small molecules and block polymers, with particle-stabilized emulsions known as Pickering emulsions.^500–502^ Building on this, the Dove and O’Reilly groups utilized polylactide PLA-based CDSA platelets for water-in-water emulsion formulation, leveraging the controlled size offered by CDSA.^142^ By premodifying the platelet-forming polymers with fluorescent dyes, the distribution of platelets on droplet surfaces was visually confirmed (Figure 39f). Larger platelets exhibited enhanced emulsifying efficiency compared to smaller ones, with emulsion stability directly correlated to platelet concentration. Stable droplets were maintained for 60 min at platelet loadings above 0.3 wt % (Figure 39g). Recently, the same groups expanded the application to oil-in-oil emulsions using PCL-based platelets, achieving emulsion stability for over 4 weeks.^125^ Given the growing demand for effective emulsifiers in pharmaceutical and cosmetic industries, these biodegradable and biocompatible platelets hold significant promise for future applications.

## 7 Challenges and Outlook

Nowadays, CDSA has emerged as a powerful strategy for fabricating nanostructures with controlled size, morphology, and hierarchical organization. Its versatility has led to advancements in biomedical applications, catalysis, optoelectronics, and nanomaterial design.^5,7,57,152,316,488^ However, several challenges remain, including improving precision, deepening mechanistic understanding, enhancing scalability for industrial applications, and broadening functional utility beyond laboratory settings. Addressing these challenges requires innovation in polymer synthesis, self-assembly methodologies, computational modeling, real-time characterization, and large-scale production strategies.

### 7.1 Enhancing Controllability and Precision

Despite significant progress, CDSA still exhibits size deviations of 10-15%, limiting the fabrication of truly monodisperse assemblies. Achieving size deviations below 10 nm, akin to protein assemblies, remains challenging. Greater precision requires advancements in polymer synthesis to achieve extremely low molecular weight dispersity. While controlled polymerization techniques such as living anionic polymerization, controlled radical polymerization, and ring-opening polymerization have improved monodispersity, further refinements are needed to achieve absolute uniformity. This can allow access to precisely regulate polymer chain lengths, dispersities, and controlled block ratios, in turn directly impacting the reproducibility and fidelity of CDSA assemblies.

Beyond polymer synthesis, improvements in self-assembly techniques are critical. Strategies such as external stimuli-driven assembly, template-guided approaches, and bioinspired hierarchical self-assembly could enhance control over nanostructure formation.^367^ High-throughput screening of CDSA conditions, including solvent selection, temperature control, and concentration optimization, is proposed to further improve reproducibility. Integrating real-time monitoring techniques would allow early detection and correction of deviations, ensuring the production of uniform, monodisperse nanoparticles.

### 7.2 Clarifying Polymer Chain Arrangement

While control over CDSA morphology has advanced considerably, understanding the molecular-level arrangement of polymer chains within assembled structures remains a major challenge. Two primary packing motifs have been widely proposed in CDSA: chain folding and π–π stacking. Chain folding is typical of semicrystalline polymers, where flexible chains fold back and stack along a defined crystallographic direction, forming ordered lamellar structures. In CDSA, such arrangements may give rise to both plate-like and elongated micelles, depending on polymer architecture and crystallization kinetics. This model, rooted in classical polymer crystallization, has been extended to explain lamellar thickness and internal ordering in polymer assemblies. By contrast, π–π stacking governs the self-assembly of conjugated or aromatic polymers, such as polythiophenes and polyfluorenes. These polymers tend to align via face-to-face interactions between planar segments, resulting in nanoribbons or rod-like micelles. Here, internal order arises from segmental stacking rather than chain folding, often with well-defined π–π distances. Critical structural parameters—such as folding thickness, stacking distance, and chain packing dynamics—remain under debate. Observed lamellar thicknesses often deviate from predictions based on idealized folding models, suggesting the presence of partial folding, chain loops, or structural defects. Similarly, π–π stacking distances are highly sensitive to backbone planarity, torsional flexibility, and side-chain packing, complicating cross-system comparisons. A comprehensive understanding of polymer packing is further limited by the spatial and temporal resolution of current analytical techniques. Methods such as cryoTEM, scanning tunnelling microscopy, and molecular simulations are increasingly employed to probe chain arrangements. Nevertheless, a unified and predictive model for molecular packing in CDSA has yet to be established.

### 7.3 Deepening Understanding of the Assembly Mechanism

The growing integration of artificial intelligence (AI) and machine learning (ML) is revolutionizing CDSA by enabling data-driven optimization. AI-assisted modeling can predict and refine self-assembly conditions, reducing experimental trial-and-error. By analyzing CDSA data sets, ML algorithms can identify key correlations and predict how variations in temperature, solvent, and polymer architecture influence final morphologies, enabling rational experimental design. Computational simulations, including molecular dynamics, Brownian dynamics, and Monte Carlo simulations, provide deeper insight into the nucleation and growth dynamics of crystalline domains. These models reveal how polymer chain orientation, crystallization rates, and interfacial interactions affect nanostructure morphology. Combining AI-driven predictions with experimental observations will transition CDSA from empirical methodologies to a predictive, mechanism-driven approach for nanomaterial fabrication.

A comprehensive understanding of CDSA requires integrating multiple characterization techniques to capture self-assembly dynamics, crystallization behavior, and nanoscale structure formation. Single-method analysis often provides an incomplete picture due to the complex interplay of thermodynamic and kinetic factors governing CDSA. Combining AFM with vibrational spectroscopy techniques such as infrared (AFM-IR) or Raman spectroscopy enables nanoscale chemical mapping alongside high-resolution morphological imaging. AFM-IR detects molecular vibrations specific to different polymer domains, revealing molecular orientation, phase separation, and crystallization heterogeneity.^403^ Liquid-TEM combined with SAXS allows real-time visualization of self-assembly dynamics, while SAXS provides quantitative insights into structural evolution. These interdisciplinary techniques refine theoretical models, optimize CDSA conditions, and provide a bridge between nanoscale interactions and macroscopic properties.

### 7.4 Enabling Scalable and Industrially Relevant CDSA Processes

A key limitation of CDSA is the reliance on dilute solution conditions, prolonged seed preparation times (typically around 1 week), and challenges in batch-to-batch reproducibility, all of which hinder scalability. In particular, the requirement for low polymer concentrations reduces production efficiency and further complicates industrial translation.^32^ Addressing these constraints necessitates strategies that enable morphological precision at higher concentrations. Continuous-flow reactor systems provide a viable solution by offering controlled reaction kinetics, temperature gradients, and solvent mixing, facilitating self-assembly under high-concentration conditions while preserving uniformity.^33,503,504^ Compared to batch processing, flow-based CDSA improves throughput, reproducibility, and consistency, making large-scale production more practical. Recently, the O’Reilly and Dove groups developed a new seed preparation method that uses flash-freezing in a flow system to improve the reproducibility, stability, and scalability of seed micelles.^33^ Further integration of AI-assisted real-time monitoring could enhance process optimization, accelerating the industrial adoption of CDSA-derived materials.

### 7.5 Exploring Potential Applications

CDSA offers a unique platform for the design and fabrication of nanostructures with precisely tunable size, shape, and internal architecture. These characteristics confer distinctive comparative advantages over other self-assembly strategies, such as micellization of amphiphilic polymers or traditional phase separation techniques. Unlike dynamic, equilibrium-based self-assemblies that often result in polydisperse or metastable structures, CDSA proceeds via a living epitaxial growth process, enabling programmable, reproducible, and highly uniform nanostructures. This level of structural control, particularly the ability to modulate dimensionality, integrate segmentation, and encode hierarchical features, positions CDSA as a useful tool for creating functional materials with tailored performance across a broad spectrum of applications including biomedicine, catalysis, optoelectronics, and smart materials.

Conventional polymer micelles often suffer from broad size distributions and limited kinetic stability, resulting in variable pharmacokinetics and reduced therapeutic reliability. In contrast, CDSA offers a unique platform for fabricating size-monodisperse nanocarriers with tunable aspect ratios and surface chemistries, which are critical determinants of *in vivo* circulation, biodistribution, and cellular uptake. Notably, CDSA assemblies possess the distinctive advantage of forming anisotropic nanostructures, which may elicit unique biological interaction profiles compared to conventional spherical assemblies. For example, anisotropic structures have been reported to exhibit differential behaviors in terms of cell surface binding, uptake pathways, and intracellular trafficking, as well as distinct metabolic processing. Furthermore, during systemic circulation, the structural anisotropy of CDSA-derived assemblies could confer tissue- and organ-specific distribution patterns, an aspect that merits further in-depth investigation to harness their full therapeutic potential. Looking ahead, future development prospects for CDSA in nanomedicine include the creation of stimuli-responsive or biodegradable assemblies capable of responding to pH changes, redox gradients, or enzymatic triggers within the tumor microenvironment, enabling controlled release and site-specific activation. Additionally, hybrid CDSA systems incorporating bioactive components such as peptides, oligonucleotides, or polysaccharides could facilitate active targeting, enhanced cellular interactions, or immunomodulatory functions, thereby expanding their utility in precision oncology and regenerative medicine.

In catalysis, structural precision and uniformity at the nanoscale or even molecular level are critical parameters, as they directly influence active site accessibility, selectivity, and overall catalytic efficiency. CDSA-derived micelles offer significant advantages in this regard by providing hierarchically ordered nanostructures with tailored surface chemistries and high surface-to-volume ratios, alongside the unique ability to spatially organize catalytic moieties in a controlled manner on the assembly surface. Functionalization of the CDSA corona with catalytic metals or organocatalysts can lead to the development of modular nanoreactors capable of enabling confined-site catalysis or facilitating cascade reactions, where substrate access and product release are finely regulated by nanoscale architecture. Importantly, the molecular-level control inherent in the CDSA process paves the way for further enhancements in catalytic efficiency, as precise positioning and uniform distribution of catalytic sites are key to achieving high turnover rates and selectivity. Moreover, incorporating stimuli-responsive blocks into the CDSA corona could enable switchable catalysis, wherein the accessibility or reactivity of active sites is modulated in response to external stimuli such as pH, temperature, or light. Such dynamic and programmable catalytic systems represent a promising direction for the design of next-generation smart catalytic materials with adaptable functionality and improved process control.

In electronic and photonic materials, CDSA offers molecular-level control critical for charge and light management. While solution-processed conjugated polymers often form poorly ordered aggregates, CDSA of π-conjugated block copolymers facilitates oriented π–π stacking and uniform nanoscale ordering, which enhances charge mobility and minimizes energetic disorder in optoelectronic devices. Besides, CDSA enables predictable, reproducible nanostructures with integrated functional domains, offering better control over ion/electron pathways and structural robustness. Future work may involve CDSA of semiconducting or donor–acceptor polymers to engineer anisotropic structures for organic photovoltaics, field-effect transistors, and photodetectors. Besides, CDSA-derived nanomaterials offer programmable architectures with chemically addressable domains, allowing selective interaction with analytes or pollutants. Their crystalline cores confer chemical robustness, while corona engineering enables tunable surface interactions. CDSA could underpin smart sensoring carriers with defined nanochannels, chemical sensors with shape-encoded selectivity, or responsive functionalities that their optical output can be altered in response to environmental stimuli (e.g., humidity, pH, heavy metals). Beyond these areas, CDSA also holds potential for the development of adaptive, stimuli-responsive materials suitable for smart coatings, nanocomposites, and functional membranes, expanding the technological impact of CDSA into dynamic and environmentally responsive systems.

While CDSA continues to demonstrate exceptional control over nanostructure fabrication, addressing challenges related to precision, mechanistic understanding, real-time characterization, scalability, and practical implementation is essential for fully unlocking its potential. The integration of AI-assisted methodologies, multimodal characterization techniques, and scalable production strategies could further accelerate the evolution of CDSA, enabling the creation of new functionalities and facilitating its widespread adoption in both scientific research and industrial applications. Realizing these opportunities will require continued cross-disciplinary synergy. Innovations at the chemistry level (e.g., new crystallizable block designs or “living” assembly mechanisms) must go hand-in-hand with advanced characterization techniques and theoretical modeling to understand and control the assembly process. Equally, input from industry stakeholders can ensure that CDSA materials meet practical requirements such as developing flow-chemistry production methods for kilogram-scale manufacturing, or ensuring compliance with biomedical regulatory standards for clinical use. Through such collaborative efforts, CDSA is poised to reshape the landscape of nanomaterials science. With its unique combination of precision, versatility, and the ability to “program” nanoparticle size and architecture, CDSA stands to become a cornerstone in the era of programmable nanomanufacturing.
